# Supplementary material for: Investigation of occupational risk factors for the development of non-Hodgkin’s lymphoma in adults: A hospital-based case-control study
Source: PLoS One. 2024 Feb 26;19(2):e0297140. doi: 10.1371/journal.pone.0297140 (PMC10896545; doi:10.1371/journal.pone.0297140)
Supplement: Attachment — Submitted filename: PONE-D-22-29162_R2_suggested corrections and changes.pdf [file pone.0297140.s004.pdf]

PLOS ONE

INVESTIGATION OF OCCUPATIONAL RISK FACTORS FOR THE DEVELOPMENT OF NON-HODGKIN LYMPHOMA IN ADULTS: A HOSPITAL-BASED CASE-CONTROL STUDY

--Manuscript Draft--

Manuscript Number:	PONE-D-22-29162R2
Article Type:	Research Article
Full Title:	INVESTIGATION OF OCCUPATIONAL RISK FACTORS FOR THE DEVELOPMENT OF NON-HODGKIN LYMPHOMA IN ADULTS: A HOSPITAL-BASED CASE-CONTROL STUDY
Short Title:	OCCUPATIONAL RISK FACTORS AND NON-HODGKIN LYMPHOMA IN ADULTS: A HOSPITAL-BASED CASE-CONTROL STUDY
Corresponding Author:	Marcia Sarpa, Ph.D<br>National Cancer Institute<br>Rio de Janeiro, RJ BRAZIL
Keywords:	non-Hodgkin lymphoma; occupational risk factors; lymphoid lineage; pesticides; solvents
Abstract:	Non-Hodgkin's Lymphoma (NHL) is a malignancy of the lymphoid lineage of the hematopoietic system and has increased worldwide, especially in developed countries. Better diagnostic and recording techniques, longer life expectancy, and greater exposure to risk factors are hypotheses for this growing incidence curve. Occupational exposures to chemical, biological, and physical agents have also been associated with NHL development, but the results are still controversial. We investigated the association between occupational exposures and risky lifestyle for NHL through a case-control study design with 214 adult patients and 452 population controls. Socio-demographic, clinical, and occupational exposure data were obtained through individual interviews with a standardized questionnaire. Clinical, laboratory, and histopathological data were obtained through medical records. Risk of NHL (any subtype), B-cell lymphoma, DLBCL, Follicular lymphoma and T-cell lymphoma was elevated among the ever exposed to any solvents [OR=2.12 (95% CI: 1.30 to 3.48); OR=1.92 (95% CI: 1.15 to 3.21); OR=3.03 (95% CI: 1.25 to 7.31); OR=3.58 (95% CI: 1.22 to 10.48)], hydrocarbon solvents [OR=1.91 (95% CI: 1.14 to 3.21); OR=1.73 (95% CI: 1.01 to 2.96); OR=2.70 (95% CI: 1.12 to 6.48)], pesticides [OR=2.70 (95% CI: 1.01 to 7.21); OR=10.65 (95% CI: 2.35 to 48.24)], meat and meat products [OR=4.19 (95% CI: 1.22 to 13.85); OR=3.29 (95% CI: 1.06 to 10.21; OR=4.73 (95% CI: 1.37 to 16.73)], and sunlight [OR=2.47 (95% CI: 1.21 to 5.04); OR=4.49 (95% CI: 1.40 to 14.36)]. A significant upward trend with years of exposure was detected for any solvents and hydrocarbon solvents (NHL (any subtype) p-value for trend<0.001), B-cell lymphoma (p-value for trend<0.001), and T-cell lymphoma (p-value for trend<0.023), pesticides (NHL (any subtype), p for trend<0.001) and T-cell lymphoma (p for trend<0.002), meat and meat products (NHL (any subtype) (p for trend<0.001) and DLBCL (p for trend<0.001), and sunlight (B-cell lymphoma (p for trend<0.001). The results of this study are in agreement with other international studies, can be extrapolated to other countries that have the same socio-demographic and occupational characteristics as Brazil and support strategies for surveillance and control of work-related cancer in the world.
Order of Authors:	Marcia Sarpa, Ph.D
	Vanessa Indio do Brasil da Costa, Ph.D
	Sâmila Natiane Ferreira, MSc
	Carolina Avila de Almeida, Doctor Degree
	Paula Gabriela Sousa de Oliveira, Doctor Degree
	Leticia Vargas de Mesquita, Doctor Degree
	Arthur OC Schilithz, Ph.D

Powered by Editorial Manager® and ProduXion Manager® from Aries Systems Corporation

	Claudio Gustavo Stefanoff, Ph.D
	Rocio Hassan, Ph.D
	Ubirani Barros Otero, Ph.D
**Response to Reviewers:**	Reply to reviewer – PLOS ONE – Second round - April 2023 – June 2023<br><br>This reviewer appreciates the authors' effort to revise the previous version. However, this new version still requires some additional effort. The first and major point is the inclusion of blood donors among the controls to balance the gender structure with the cases. Blood donors, by definition, are healthier than the general population and, therefore, their inclusion makes any attempt of exploring the association with the health history severely and irreparably biased. Nonetheless, the paper has some good points too, which can be saved provided that the authors keep complying with the reviewers' comments and suggestions.<br>Answer:<br>The controls were selected from the companions and visitors of patients diagnosed with solid cancers and also from the waiting room of the Hospital's blood bank, which also receives mostly visitors and patients' companions. This procedure was adopted to minimize the effect of a possible control selection bias. In this way, the controls selected at the Blood Bank did not differ from the controls collected in the Hospital wards, as they are also visitors or companions of the patients. It should also be noted that INCA is not a blood center. In order to not cause problems in the interpretation, the term "biological material donors" was replaced by "blood bank controls".<br>One suggestion would be to omit the analysis of the past health history and focus on the analysis of occupational risk factors. The adjustments by alcohol and smoking can at least partially fix the selection bias related to the blood donors' healthier status. If the authors wish to maintain the analyses on past illnesses, they should limit them to the 309 controls recruited among the patients' companions and visitors.<br>In any case, the selection bias introduced by using blood donors as controls needs to be openly acknowledged and discussed among the limitations of this study.<br>The discussion should start with the main results, which are those related to the occupational risk factors. Lifestyle variables were only evaluated in the univariate analysis and smoking, and alcohol only considered as covariates in the regression models. Therefore, a short comment would be enough.<br>Ok.<br>Consistently with the bias generated by using blood donors as controls, all the results and discussion on diabetes, HTLV and HCV should be omitted. Those on diabetes and perhaps HCV might be briefly commented upon in the case the authors repeated the analysis after excluding the 143 blood donors. Also, rerun the logistic regressions that included diabetes and HHV8 infection as covariates.<br>Parts of the diabetes, HTLV, and HCV discussions were omitted as directed.<br>The results on metals seem also contradictory; a short comparison with the literature findings would be enough.<br>Ok<br>From Table 2, it seems that the cases had a lower socio-economic status, but the list of covariates in the footnotes of table 3 do not include any specific adjustment. This might have contributed to generate some possibly spurious excess, such as that with exposure to sunlight. This reviewer suggests including education in the regression models, as listed on line 193 under the subheading on statistical methods as a surrogate for socio-economic status. Also, include occupation as a farmer as a covariate in the logistic regression predicting risk of NHL & subtypes associated with exposure to sunlight<br>All potential confounding factors of the theoretical model were tested, as well as the level of education was used in the first part of the analysis with the selection criterion (Wald p-value < 0.025). In the multivariate analysis, other socioeconomic variables quantified the variability of response variables, such as place of residence and family income. Only three cases self-declared one of the occupations as a farmer and two as cattle ranchers, therefore, it is not possible to carry out this specific analysis.<br>At the end of the discussion, the authors should insert a sub-section on the study limitations: selection bias, multiple comparisons, recall bias due to the self-report of the occupational exposure, years of exposure as a poor surrogate of cumulative exposure, lack of exposure assessment by experts to define other exposure metrics, such as intensity and frequency of exposure, the generic definition of the exposure categories,

Powered by Editorial Manager® and ProduXion Manager® from Aries Systems Corporation

possible confounders unaccounted for (socioeconomic status, agricultural work and exposure to sunlight). The effects of all the above on the risk estimates needs a thorough discussion.
Done.
The text requires an in-depth revision of the style, grammar, and typos. This reviewer went through this type of editing as well for the abstract, introduction, methods and results, but the list in the attached document, though long, might not be exhaustive.
The final version of the article after all the revisions suggested by the reviewers will be sent to the translator.
PLOS One
pone-d-22-29162r1

Investigation of occupational risk factors for the development of non-Hodgkin lymphoma in adults: a hospital-based case-control study.
Additional list of editings.
Abstract
The abstract is too long, includes unnecessary information, and omits important results. For instance, the long sentence in lines 38-45 can be replaced with "We explored the association of NHL risk occupational and lifestyle exposures [and health history, if the authors wish to pursue their analysis using only companion or visitor controls] with a case-control study design in 214 adult patients and 452 population controls in Brazil."
Ok.
Remove the sentence about ethical approval in lines 47-49 and paste it at the end of the Methods section.
Ok. Removed
The abstract should report the odds ratio and 95% confidence interval associated with ever exposure to the occupational exposures showing a significant association, and the respective p-value of the test for trend. The p-value of the test for trend should also be reported in Table 3. The lines 49-56 in the abstract should be replaced with the following: "risk of NHL (any subtype), B-cell lymphoma, and T-cell lymphoma was elevated among the ever exposed to solvents [give the Odds ratio and 95% confidence interval associated with ever exposure (=all the exposed)], pesticides [Odds ratio and 95% CI for the ever exposed], meat and meat products [instead of animal and visceral materials] [Odds ratio and 95% CI for the ever exposed], and sunlight [instead of solar radiation] [Odds ratio and 95% CI for ever exposed]. A significant upward trend with years of exposure was detected for solvents and hydrocarbon solvents (NHL (any subtype) p-value for trend), B-cell lymphoma (p for trend), and T-cell lymphoma (p for trend)), pesticides (NHL (any subtype), p for trend) , B-cell lymphoma (p for trend), DLBCL (p for trend), and T-cell lymphoma (p for trend), raw meat (NHL (any subtype) (p for trend) and DLBCL (p for trend), and sunlight (B-cell lymphoma (p for trend), DLBCL [? Not sure] (p for trend), and follicular lymphoma [if still significant after adjustment for agricultural work] (p for trend)".
Ok. It was done.
The results for metals are not worth reporting in the abstract as they are non-significant and show an inverse trend with years of exposure.
Ok.
Drop the sentence in lines 57-58 from "…can be extrapolated to other countries" through "…Brazil" and, in the following sentence, on line 58, delete "…will".
Ok
Introduction
On line 67, replace "which" with "and".
Ok
Lines 71-72. In all case series this reviewer is aware of, CLL/SLL is among the most frequent mature B-cell subtypes, sometimes the most frequent. It might be less so in South America,or Asia, but certainly is not definable as a "less common" subtype". A lesser frequency might be due to underdiagnosis because of the silent clinical course of most cases. Just omit mentioning CLL here.
Ok
Lines 96 – 98; include the two citations reported here with the name of the first author and year in the list of references, renumber the references progressively in order of citation, and replace name and year with the corresponding numbers throughout the manuscript.
Ok

	Line 98: replace “not yet consistent” with “inconsistent”.<br>Ok<br>Line 99-101: Marant Micallef only summarized the IARC Working Groups’ conclusions, without making any further elaboration. So, this reviewer would suggest replacing the whole sentence with the following “The International Agency for Research on Cancer classifies 87 occupational exposures in groups 1 and 2A [13]”.<br>Ok<br>Line 102: replace “were attributed to non-Hodgkin’s lymphomas” with “were specifically associated with non-Hodgkin’s lymphoma”.<br>Ok<br>Methods<br>Line 113: replace “that reside” with “residing”.<br>Ok<br>Line 114; delete “…and were…”.<br>Ok<br>Line 117: replace “despite” with “although”.<br>Ok<br>Lines 118-121: delete from “…is a reference hospital…” through “…Government of Brazil,”<br>Ok<br>Line 123: delete the sentence “…and INCA is also responsible…”; insert the following after “…(CANCON)”: “INCA is a national institute, part of the Ministry of Health of the Federal Government of Brazil, responsible for providing care to around 7.000 new patients per year and…“.<br>Ok<br>Line 128: delete “…it was decided to calculate”.<br>Ok<br>Line 128: insert “…was determined using…” between “…sample size” and “…with the following”.<br>Ok<br>Line 129: insert “case-control” between “2:1” and “ratio”.<br>Ok<br>Line 130: delete the closing bracket.<br>Ok<br>Lines 130-131: replace “…for a statistical power of 80%, it will be necessary 215 cases and 430 controls” with “…215 cases and 430 controls would be required to achieve a statistical power of 80%”.<br>Ok<br>Lines 134-138: replace the sentence currently in lines 134-138 with the following: “Eligible controls were visitors or companions of patients at INCA/HCI, aged between 25 and 75 years and free from any type of cancer at the time of participating in the study”.<br>Ok<br>Line 139: insert the following after “…557 controls”: “matched to cases by gender and age-group (± 10 years)”<br>Ok<br>Lines 141: delete “…previously established…”. Replace “Regarding controls, 105 subjects (18.9%)…” with “…One -hundred-five candidates as controls (18.9%)…”.<br>Ok<br>Line 142: In case the authors wish to pursue in their analysis of health history, they should explain here the reason for selecting blood donors and indicate their number (N = 143). This reviewer would suggest including a sentence such as: “As most visitors of patients diagnosed with non-haematological neoplasms at the hospital's outpatient clinics or wards were female, it was necessary to recruit male blood donors (N = 143) at the hospital Haemotherapy Service.” If the authors decide of omitting the analysis of health history, just delete the whole sentence.<br>Ok<br>Lines 149-151: report here the number of cases for each NHL subgroup.<br>Ok<br>Lines 152-159: delete the whole paragraph.<br>Ok<br>Lines 160-162: move the Ethics note to the end of the Methods section.<br>Ok<br>Line 167: delete “…individual”.

Powered by Editorial Manager® and ProduXion Manager® from Aries Systems Corporation

	Ok<br>Lines 178-183. Delete these sentences [see note about line 142].<br>Ok<br>Lines 184-188: Delete the whole sentence as it is repetitive.<br>Ok<br>Line 189: delete “Thus, in order…”<br>Ok<br>Line 190: replace “…collected on the subject’s” with “…abstracted for analysis”.<br>Ok<br>Line 192: delete “race (white, non white)”. [following my previous review, race was excluded as a covariate]<br>Ok<br>Line 193: replace “time of residence” with “years at the current residence”.<br>Ok<br>Line 195: replace “not employed” with “unemployed”.<br>Ok<br>Line 196-197: Table 1 does not show what was the unit for average income.<br>Ok<br>Line 198 and the below paragraph: See the comments on line 142. Also replace “In relation to habits and past illness were included..” with “Among the lifestyle variables, we considered the following:…”.<br>Ok<br>Lines 201-204: delete from “…past illnesses…” through the end of the sentence.<br>Ok<br>Line 205: replace “…their…” with “…the…”<br>Ok<br>Line 206: replace “working time greater than 1 year” with “lasting one year or more”.<br>Ok<br>Line 206-209: delete the unnecessary explanation for cutting short-term employments.<br>Ok<br>Line 212: “biological material, animal and viscera material”. Please, explain what the biological materials would be or whether the authors meant “infectious agents”. Also, the animal and viscera material, as this reviewer understand, would be the meat aerosol in abattoirs and butcheries. If so, the definition should be raw meat droplets and aerosols. Some information on what occupation were related to such exposures should be included in the Table 3 footnote.<br>The question asked through the questionnaire is very broad. You are asked about exposure to biological materials in the work environment (eg such as blood, urine or other exposures).<br>Line 213: replace “...collected…” with “…self-reported…”.<br>Ok<br>Lines 232-235: replace “By unconditional logistic regression, multivariate models were estimated to analyse exposure to chemical, physical, and biological agents as well as the risk of NHL according to the cases “Total NHL”, “B-cell lymphoma”, “DLBCL”, “Follicular lymphom, and “T-cell lymphoma”. The covariates used in the study were:” with the following: “Risk of NHL (any subtype), B-cell lymphoma, DLBCL, Follicular lymphoma, and T-cell lymphoma associated with exposure to chemical, physical, and biological agents was analysed by unconditional regression. The multivariate regression model included the following covariates:”<br>Ok<br>Line 236: delete “past illnesses” and “also”.<br>Ok<br>Lines 238-245: Replace the whole sentence with the following: “To select the covariates to be included in the final regression model, we followed a stepwise forward procedure testing first the covariates with a p<0.25 association in the univariate analyses of the covariates with NHL (any subtype)[15].”<br>Ok<br>Drop diabetes and HHV8 infection from the covariates in the logistic regression model.<br>Parts of the diabetes, HTLV, and HCV discussions were omitted as directed.<br>Lines 245-247 “replace the whole sentence with the following: “The Odds Ratio and its 95% confidence interval were calculated as the measure of association; the null hypothesis was rejected if the 95% confidence interval did not include unity.”<br>Ok

	Results<br>The data reported in the first sentence should be moved to the Methods: the number of participants by gender would fit in line 160, before the flow diagram in figure 1; the number of cases of each NHL subtype would fit in lines 234-235. No supplementary material is needed.<br>Ok<br>Line 257-259: delete this sentence.<br>Ok<br>Table 1. Replace “time of residence” with “years of residence”. Please show what was the unit for family income.<br>Ok<br>Lines 280-282: Delete the sentence on past illnesses and the corresponding results from table 2.<br>Excluded.<br>Lines 291-293: delete from “…of occupational…” through “…histological subtypes.”<br>Ok<br>Line 294: insert ”any of” between “exposed to” and “those substances..”.<br>Ok<br>Line 297: Risk increased also for several other subtypes and other exposures. Please. report here the p-value for the tests for trend.<br>Trend p-value values are described in table 3.<br><br>Lines 298-310. Move these sentences from line 296, before the sentence on tests for trend, and report in the text the risks associated with ever exposure (i.e. independent on years of exposure).<br>Ok<br><br>Lines 317-321: move the result on T-cell lymphoma and pesticides before reporting about the tests for trend (line 315). Also, make it shorter as the small number of cases and controls suggests caution. Something like: “the strongest association was observed with risk of T-cell lymphoma, although the small number of subjects suggests caution in the interpretation”.<br>Ok<br><br>The same suggestion is valid in reporting the results for raw meat aerosols and exposure to sunlight.<br>Ok<br><br>Table 3: Insert risk for ever exposure (all the exposed one or more years independent on years of exposure) for each occupational risk factor. Calculate and report the p-value for each test for trend. It is unclear why some results are missing and others with smaller number and presumably more unstable estimates are shown instead (see for instance B-cell lymphoma and dyes, metals, biological agents, and ionizing [and not industrial] radiation or T-cell lymphoma and sanitizing products vs T-cell lymphoma and metals. In the footnote, insert what occupations were more frequently represented among the exposto biological materials and sunlight.<br>Occupations that most represent exposure to biological materials: Nurse, nursing technician.<br>Occupations that represent the most exposure to solar radiation: tradesman, housemaid, housewife, electrician, driver, mason, painter, military police, security, salesman.
**Additional Information:**	
**Question**	**Response**
**Financial Disclosure**<br><br>Enter a financial disclosure statement that describes the sources of funding for the work included in this submission. Review the submission guidelines for detailed requirements. View published research articles from *PLOS ONE* for specific	Vanessa Indio do Brasil da Costa received an institutional development scholarship from Brazilian National Cancer Institute (INCA);<br>Sâmila Natiane Ferreira and Letícia Vargas de Mesquita received a scholarship from CNPQ/INCA;<br>Carolina Ávila de Almeida and Paula Gabriela Sousa de Oliveira received a scholarship from FAPERJ;<br><br>The Pan-American Health Organization (PAHO) financed the study through the

examples.<br><br>This statement is required for submission and **will appear in the published article** if the submission is accepted. Please make sure it is accurate.<br><br>**Unfunded studies**<br>Enter: *The author(s) received no specific funding for this work.*<br><br>**Funded studies**<br>Enter a statement with the following details:<br>• Initials of the authors who received each award<br>• Grant numbers awarded to each author<br>• The full name of each funder<br>• URL of each funder website<br>• Did the sponsors or funders play any role in the study design, data collection and analysis, decision to publish, or preparation of the manuscript?<br>• **NO** - Include this sentence at the end of your statement: *The funders had no role in study design, data collection and analysis, decision to publish, or preparation of the manuscript.*<br>• **YES** - Specify the role(s) played.<br><br>* typeset	INCA/PAHO TC 43 agreement.<br>The funders had no role in study design, data collection and analysis, decision to publish, or preparation of the manuscript.
**Competing Interests**<br><br>Use the instructions below to enter a competing interest statement for this submission. On behalf of all authors, disclose any competing interests that could be perceived to bias this work—acknowledging all financial support and any other relevant financial or non-financial competing interests.<br><br>This statement is required for submission and **will appear in the published article** if the submission is accepted. Please make sure it is accurate and that any funding sources listed in your Funding Information later in the submission form are also declared in your Financial Disclosure statement.	The authors have declared that no competing interests exist.

View published research articles from *PLOS ONE* for specific examples.<br><br>**NO authors have competing interests**<br><br>Enter: *The authors have declared that no competing interests exist.*<br><br>**Authors with competing interests**<br><br>Enter competing interest details beginning with this statement:<br><br>*I have read the journal's policy and the authors of this manuscript have the following competing interests: [insert competing interests here]*<br><br>* typeset	
**Ethics Statement**<br><br>Enter an ethics statement for this submission. This statement is required if the study involved:<br><br>• Human participants<br>• Human specimens or tissue<br>• Vertebrate animals or cephalopods<br>• Vertebrate embryos or tissues<br>• Field research<br><br>Write "N/A" if the submission does not require an ethics statement.<br><br>General guidance is provided below. Consult the submission guidelines for detailed instructions. **Make sure that all information entered here is included in the Methods section of the manuscript.**	The letters of informed consent, questionnaires, and all other correspondence with study participants were approved by the INCA Research Ethics Committee under registration number 135/11.

**Format for specific study types**<br><br>**Human Subject Research (involving human participants and/or tissue)**<br>• Give the name of the institutional review board or ethics committee that approved the study<br>• Include the approval number and/or a statement indicating approval of this research<br>• Indicate the form of consent obtained (written/oral) or the reason that consent was not obtained (e.g. the data were analyzed anonymously)<br><br>**Animal Research (involving vertebrate animals, embryos or tissues)**<br>• Provide the name of the Institutional Animal Care and Use Committee (IACUC) or other relevant ethics board that reviewed the study protocol, and indicate whether they approved this research or granted a formal waiver of ethical approval<br>• Include an approval number if one was obtained<br>• If the study involved *non-human primates*, add *additional details* about animal welfare and steps taken to ameliorate suffering<br>• If anesthesia, euthanasia, or any kind of animal sacrifice is part of the study, include briefly which substances and/or methods were applied<br><br>**Field Research**<br><br>Include the following details if this study involves the collection of plant, animal, or other materials from a natural setting:<br>• Field permit number<br>• Name of the institution or relevant body that granted permission	
**Data Availability**<br><br>Authors are required to make all data underlying the findings described fully available, without restriction, and from the time of publication. PLOS allows rare exceptions to address legal and ethical concerns. See the PLOS Data Policy and FAQ for detailed information.	Yes - all data are fully available without restriction

A Data Availability Statement describing where the data can be found is required at submission. Your answers to this question constitute the Data Availability Statement and **will be published in the article**, if accepted.<br><br>**Important:** Stating ‘data available on request from the author’ is not sufficient. If your data are only available upon request, select ‘No’ for the first question and explain your exceptional situation in the text box.<br><br>Do the authors confirm that all data underlying the findings described in their manuscript are fully available without restriction?	
**Describe where the data may be found in full sentences. If you are copying our sample text, replace any instances of XXX with the appropriate details.**<br><br>• If the data are **held or will be held in a public repository**, include URLs, accession numbers or DOIs. If this information will only be available after acceptance, indicate this by ticking the box below. For example: *All XXX files are available from the XXX database (accession number(s) XXX, XXX.).*<br>• If the data are all contained **within the manuscript and/or Supporting Information files**, enter the following: *All relevant data are within the manuscript and its Supporting Information files.*<br>• If neither of these applies but you are able to provide **details of access elsewhere**, with or without limitations, please do so. For example:<br><br>*Data cannot be shared publicly because of [XXX]. Data are available from the XXX Institutional Data Access / Ethics Committee (contact via XXX) for researchers who meet the criteria for access to confidential data.*<br><br>*The data underlying the results presented in the study are available from (include the name of the third party*	All relevant data are within the manuscript.

Powered by Editorial Manager® and ProduXion Manager® from Aries Systems Corporation

and contact information or URL).<br>• This text is appropriate if the data are owned by a third party and authors do not have permission to share the data.<br><br>* typeset	
Additional data availability information:	Tick here if the URLs/accession numbers/DOIs will be available only after acceptance of the manuscript for publication so that we can ensure their inclusion before publication.

Powered by Editorial Manager® and ProduXion Manager® from Aries Systems Corporation

Manuscript Click here to access/download;Manuscript;VF_Manuscript_LNH_30_06_202

INVESTIGATION OF OCCUPATIONAL RISK FACTORS FOR THE DEVELOPMENT OF NON-HODGKIN LYMPHOMA IN ADULTS: A HOSPITAL-BASED CASE-CONTROL STUDY

Marcia Sarpa[1,2,*], Vanessa Índio do Brasil da Costa[1], Sâmila Natiane Ferreira[1,2], Carolina Ávila de Almeida[1,2], Paula Gabriela Sousa de Oliveira[1,2], Letícia Vargas de Mesquita[1,2], Arthur OC Schilithz[1], Claudio Gustavo Stefanoff[3], Rocio Hassan[4#], Ubirani Barros Otero[1].

[1]Coordination of Prevention and Surveillance, Brazilian National Cancer Institute. Rua Marquês de Pombal, 125 – Centro, Rio de Janeiro – Brazil. CEP: 20230-240. Phone: +55 (21) 3207-6089.

[2]Environmental Mutagenesis Laboratory, Federal University of Rio de Janeiro State (UNIRIO). Rua Frei Caneca, 94 – Centro, Rio de Janeiro – Brazil. CEP 20211-040. Phone: +55 (21) 2531-7998.

[3]Coordination of Clinical Research and Technological Incorporation, Brazilian National Cancer Institute. Rua André Cavalcanti 37, 2° andar – Bairro de Fátima, Rio de Janeiro, RJ – Brazil. CEP: 20231-050. Phone: +55 (21) 3207-6541.

[4]Oncovirology Laboratory. Bone Marrow Transplantation Center. Brazilian National Cancer Institute (INCA). Praça da Cruz Vermelha, 25 – Centro, Rio de Janeiro – Brazil. CEP: 20230-240. Phone: +55 (21) 3207-1000

[#]in memorium

***Corresponding Author:** Marcia Sarpa.

[1]Institution: Coordination of Prevention and Surveillance. Brazilian National Cancer Institute. Adress: Rua Marquês de Pombal, 125, 5°andar – Centro, Rio de Janeiro – Brazil. Zipcode: 20230-240. Phone: +55(21)3207-6092. E-mails: marcia.sarpa@inca.gov.br; marciasarpa@gmail.com

ABSTRACT

Non-Hodgkin's Lymphoma (NHL) is a malignancy of the lymphoid lineage of the hematopoietic system and has increased worldwide, especially in developed countries. Better diagnostic and recording techniques, longer life expectancy, and greater exposure to risk factors are hypotheses for this growing incidence curve. Occupational exposures to chemical, biological, and physical agents have also been associated with NHL development, but the results are still controversial. We investigated the association between occupational exposures and risky lifestyle for NHL through a case-control study design with 214 adult patients and 452 population controls. Socio-demographic, clinical, and occupational exposure data were obtained through individual interviews with a standardized questionnaire. Clinical, laboratory, and histopathological data were obtained through medical records. Risk of NHL (any subtype), B-cell lymphoma, DLBCL, Follicular lymphoma and T-cell lymphoma was elevated among the ever exposed to ***any solvents*** [OR=2.12 (95% CI: 1.30 to 3.48); OR=1.92 (95% CI: 1.15 to 3.21); OR=3.03 (95% CI: 1.25 to 7.31); OR=3.58 (95% CI: 1.22 to 10.48)], ***hydrocarbon solvents*** [OR=1.91 (95% CI: 1.14 to 3.21); OR=1.73 (95% CI: 1.01 to 2.96); OR=2.70 (95% CI: 1.12 to 6.48)], ***pesticides*** [OR=2.70 (95% CI: 1.01 to 7.21); OR=10.65 (95% CI: 2.35 to 48.24)], ***meat and meat products*** [OR=4.19 (95% CI: 1.22 to 13.85); OR=3.29 (95% CI: 1.06 to 10.21; OR=4.73 (95% CI: 1.37 to 16.73)], and ***sunlight*** [OR=2.47 (95% CI: 1.21 to 5.04); OR=4.49 (95% CI: 1.40 to 14.36)]. A significant upward trend with years of exposure was detected for ***any solvents*** and ***hydrocarbon solvents*** (NHL (any subtype) p-value for trend<0.001), B-cell lymphoma (p-value for trend<0.001), and T-cell lymphoma (p-value for trend<0.023), ***pesticides*** (NHL (any subtype), p for trend<0.001) and T-cell lymphoma (p for trend<0.002), ***meat and meat products*** (NHL (any subtype) (p for

trend<0.001) and DLBCL (p for trend<0.001), and ***sunlight*** (B-cell lymphoma (p for trend<0.001). The results of this study are in agreement with other international studies, can be extrapolated to other countries that have the same socio-demographic and occupational characteristics as Brazil and support strategies for surveillance and control of work-related cancer in the world.

Keywords: Non-Hodgkin Lymphoma; occupational risk factors; lymphoid lineage; pesticides; solvents.

INTRODUCTION

Non-Hodgkin's lymphomas (NHLs) are a group of malignant diseases with more than 40 main subtypes, and which have distinct genetic, morphological, and clinical characteristics [1]. They originate from progenitor cells and mature B cells, from progenitor and mature T-cells or from natural killer cells (rarely), which vary in pathophysiology, histological appearance, clinical course, and response to therapy [2]. The most common subtypes are diffuse large B-cell and follicular lymphomas, together accounting for approximately 30 and 20% of all NHL cases, respectively [3,4]. Other less common subtypes are Burkitt lymphoma (1–5% of adult lymphoma cases); lymphoplasmacytic lymphoma (rare and indolent); chronic lymphocytic leukemia/small cell lymphocytic lymphoma; marginal zone lymphoma (5–10% of NHL cases); mantle cell lymphoma (2–4% of NHL cases); peripheral T-cell lymphoma, which the WHO defines as "natural killer" and mature T-cell neoplasms, and Mycoses fungoides/Sezary syndrome [1].

The highest incidence rates of NHL are found in industrialized countries in North America and Europe, in addition to Australia and New Zealand (> 7.0/100,000 thousand, in

both men and women) [5]. ~~Each year, an estimated 509,590 new cases of NHL (2.7% of total cancer) and around 250,000 deaths (2.4% of total deaths) occur worldwide.~~ NHL is among the top ten most common types of cancer in Brazil~~, each year.~~ For the triennium 2023-2025, an estimated 6,420 and 5,620 new cases will occur annually in Brazilian men and women, respectively. This corresponds to an estimated rate of 6.08 new cases per 100,000 men and 5.08 per 100,000 women. The highest incidence rates of NHL are observed in men in the Southeastern region (8.2/100 thousand) and in women in the Southern Region (7.3/100 thousand) [6].

The best-established risk factors for NHL are immunosuppression; infections, including HIV/AIDS virus, hepatitis C, Epstein Bar; and autoimmune diseases [7]. ~~The proportion of NHL cases related to celiac disease, systemic lupus erythematosus (SLE), Sjogren's syndrome, and rheumatoid arthritis (RA), for example, is considered small, due to the low prevalence of these diseases in the general population [4,8,9].~~ Hereditary factors are also cited, but they are responsible for only a small portion of NHL cases [9]. Other factors for the development of NHL include smoking (follicular lymphoma) and occupation, especially occupational exposure to specific chemical, physical, and biological agents.

~~The chemical agents~~ most consistently associated with NHL~~, mentioned in the literature, are~~ ~~solvents and pesticides~~ [7,10,11]. Other occupational factors related to NHL include sunlight [12]; ionizing radiation [11]; organic dust (grain, leather, fabric, and wood dusts) [13,14]; however, evidence of the association of these factors with NHL is in not consistent [7].

The International Agency for Research on Cancer (IARC), classifies ~~87 occupational exposures in groups 1 and 2A [13] (68 from group 1 and 19 from group 2A). Nine of these~~

exposures were attributed were specifically associated with non-Hodgkin's lymphoma, with only two considered to have sufficient evidence in humans.

Thus, the aim of the present study was to describe the socio-demographic and clinical data of patients treated at the Brazilian National Cancer Institute, in addition to assess whether there is an association between occupational exposures to chemical, physical and biological agents and the chance of developing NHL in these patients. Also, it is expected that the results of this study support the implementation of actions to prevent and control exposure to carcinogens in the work environment.

MATERIALS AND METHODS

Study design and population

This is a hospital-based case-control study, with patients residing in the state of Rio de Janeiro (RJ, Brazil) and were seen at the Brazilian National Cancer Institute (INCA). The project was approved by the Research Ethics Committee of the INCA under registration number 135/11.

It is noteworthy that 17.5 million people live in the 92 municipalities in the state of Rio de Janeiro (RJ, Brazil). Therefore, despite the case-control study was carried out in a single institute in the country, this institute is a reference hospital for the care of cancer patients in Brazil, being a High Complexity Assistance Centers in Oncology (CACON). INCA is a national institute, part of the Ministry of Health of the Federal Government of Brazil, responsible for providing care to around 7.000 new patients per year and is also responsible for formulating public policies on cancer in Brazil.

The sample size calculation was performed using the EpiInfo software version 3.3.2~~,~~ ~~using the calculation basis for case-control studies,~~ for a confidence interval of 95%. Considering human exposure to the main carcinogens described in the literature associated with lymphomas, it was decided to calculate the sample size using the following parameters: α = 0.05; case-control 2:1 ratio; prevalence of occupational hazards among controls of 5% (adopting a more conservative measure; OR = 2.5). Thus, 215 cases and 430 controls would be required to achieve a statistical power of 80%.

Inclusion criteria for the cases were: age between 25 and 75 years; residents of the state of Rio de Janeiro; Brazilian nationality; with a primary diagnosis of NHL confirmed by laboratory and histopathological tests. ~~Exclusion criteria for the cases were:~~ patients ~~diagnosed with HIV~~ were ~~not included~~.

Eligible controls were visitors or companions of patients at INCA/HCI, aged between 25 and 75 years ~~and~~ free from any ~~type of~~ cancer at the time of participating in the study. ~~Inclusion criteria for the control group were: visitors or companions of patients at INCA/HCI, aged between 25 and 75 years, without previous or current diagnosis of any type of cancer~~ and not accompanying or visiting patients diagnosed with hematological cancer.

During the study, 274 cases and 557 controls matched to cases by gender and age-group (± 10 years) were invited to participate in the study. Of these, 10 cases (3.6%) refused to participate in the study and 50 cases (18.2%) did not meet the previously established eligibility criteria. Regarding controls, 105 (18.9%) did not meet the eligibility criteria or refused to participate in the study. As most visitors of patients diagnosed with non-haematological neoplasms at the hospital's outpatient clinics or wards were female, it was necessary to recruit male blood donors (N = 143) ~~at the hospital Haemotherapy Service.~~ A

total of 214 NHL cases (112 men and 102 women) and 452 controls (207 men and 245 women) were included in the study. The complete diagram with the initial and final number of study participants, including refusals, can be seen in Figure 1.

Therefore, this study includes 214 incident patients between the ages of 25 and 75 years with NHL (International Classification of Disease - ICD10; C82-85) diagnosed between 2013 and 2016. Hospital-based cancer registries of cases were identified, and histopathological reports were examined to confirm the diagnosis of NHL, based on the WHO classification: Follicular non-Hodgkin's lymphoma (C82) N cases; Diffuse non-Hodgkin lymphoma (C83) N cases; Cutaneous and peripheral T-cell lymphomas (C84) N cases; Non-Hodgkin lymphoma of other and unspecified types (C85) N cases) [15].

Fig 1. Research participants flow diagram

Data collection

The individual interviews of the research participants were carried out by qualified and trained professionals from the Technical Area of Environment, Work and Cancer of the National Cancer Institute (INCA; Ministry of Health; Brazil). On the day of the medical appointment or some routine procedure at the hospital, the patients were invited to participate in the research by professionals from the INCA research group. Those patients who agreed to participate in the research signed the Free and Informed Consent Form (TCLE). Data collection was carried out through a standardized and pre-tested questionnaire with questions aimed at obtaining sociodemographic, clinical and occupational information.

In the same period, companions and visitors of patients diagnosed with non-hematological neoplasms undergoing medical care at the outpatient clinics or wards of the Cancer Hospital I (HCI) of the National Cancer Institute (INCA) were invited to participate in the research to compose the control group. In addition, there was a need to recruit donors of biological materials in the Hemotherapy Service (HC1/INCA), since most of the companions and visitors of patients diagnosed with non-hematological neoplasms undergoing medical care at the hospital's outpatient clinics or wards INCA HCI were female. Therefore, on recruitment days, donors of biological materials at the Hemotherapy Service (HC1/INCA) were also invited to participate in the research.

The main outcome of the study was total NHL, and secondary outcomes, such as subtypes B-cell lymphoma, DLBCL, T-cell lymphoma were also included in the study. Occupational exposures were considered as explanatory variables. In addition to these exposures, the literature mentions age, gender, race/ethnicity, family history, autoimmune diseases and some infections as risk factors for NHL.

In order to characterize the study population and control possible confounding factors, the following information was collected: demographic and clinic variables, including "year of interview" (2013, 2014, 2015 or 2016), "sex" (male, female), "age" (20-39; 40-59; >=60), "race" (white, no white), "region within the state of Rio de Janeiro" (metropolitan, others), "*years at the current residence* time of residence" (≤ 20, >20), "education" (no degree of education or primary school, high school, university degree), "employment actual": yes (private institution, retired, self-employed, public institution, not employed, dependent, pensioner, sick leave), "marital status (no companion, with companion, widowed), "family income - minimum salaries" (mean/SD).

Among the lifestyle variables, we considered the following: “smoking habit” (never smoked, current smoker, past smoker: ≤ 10 years, past smoker: 10+ years), ~~“smoke load: pack years”~~ (never, daily: <10years, 10+years, others), “cellular phone use” (never, daily: ≤ 10 years, daily: 10+years, others)., “past illnesses” (dengue, pneumonia, ~~HHV8 infection:~~ herpesvirus-8, asthma, *H. pylori* infection, diabetes, tuberculosis, HCV infection: hepatitis C virus, malaria, HTLV infection, ~~EBV infection:~~ Epstein-Barr virus). For this last variable (“past illnesses”), more than one response was considered.

The occupational history was limited to their main occupations in the last 20 working years ~~and all occupations with working time greater than~~ 1 year ~~were considered~~. ~~It was decided to establish a cut-off point lasting one year or greater than 1 year for the exposure time, due to the difficulty of making a causal link between the short exposure time and the development of non-Hodgkin's lymphoma. Inform~~ Information on occupational exposure to chemical ~~substances,~~ physical and biological agents possibly associated with the risk of ~~developing~~ NHL such as solvents, paints, metals, pesticides, cleaning products, biological material, meat and meat products, industrial radiation and sunlight (never, <= 10 years, 10+ years) were self-reported ~~collected. The participant responded about which agents he was exposed to and for how long, in the last 20 years,~~ for each ~~reported~~ occupation. ~~Although the questionnaire contains this information, it was decided to present the results keeping the exposures grouped (any solvents, hydrocarbons solvents, pesticides, metals, meat and meat products, and sunlight), without specifying which solvents, metals or pesticides were reported.~~ ~~The description of the exposures reported by the study participants, which agents were contained in each group, can be seen at the bottom of Table 3~~

Statistical analysis

~~A descriptive analysis was performed to verify the profile of the studied population. The variables were categorized according to the cutoff points pre-established in the literature~~. To assess differences in the distributions of categorical variables between ~~the comparison groups (~~cases and controls~~)~~, Pearson's chi-square tests or Fisher's exact test (for cases where the variable was dichotomous and there were less than five individuals in a cell) were performed. Missing values were excluded from the analysis and the information was reported in tables 1 and 2. For continuous variables, normality was tested (Kolmogorov test). When the variable had a normal distribution, Student's t-test was used to compare the means; otherwise, the Mann-Whitney non-parametric test was used.

~~By unconditional logistic regression, multivariate models were estimated to analyze exposure variables to chemical, physical, and biological agents as well as the risk of NHL according to the cases~~ "Total NHL", "B-cell lymphoma", "DLBCL", "Follicular lymphoma", and "T-cell lymphoma"~~. The covariates used in the study were:~~ gender, age, skin color, family income, place of residence, level of education, smoking, alcoholism, past illnesses, and ~~also~~ family history of hematological cancer, including ~~the history of~~ NHL (described in the "Data collection" section and in the ~~footer~~ of table 3). ~~The first step was select the covariates to be included in the final regression model, to each outcome and exposure, in the univariate analyses with NHL at p-value < 0.250 [15]. After, to the multivariate analysis, we followed a manual stepwise forward procedure (most significant variables entered first), where the occupational exposure of interest was tested and the adjustment of each model was evaluated by inserting each of the previously selected covariates~~..

At the end, a 5% significance level was considered for the control of possible confounding assessed using the odds ratio (OR) and its respective 95% confidence interval (95% CI). The software used was SPSS 25.0.

The letters of informed consent, questionnaires, and all other correspondence with study participants were approved by the INCA Research Ethics Committee under registration number 135/11.

RESULTS

Regarding the cases of NHL, the predominant histological type was B-cell lymphoma (185 cases; 86.0%) and T-cell lymphoma were 30 cases (14.0%).

There was no statistical difference in age (p = 0.063) between the two groups. Most individuals were older than 40 years, with mean age of cases (56 years; standard deviation, SD = 12.7 years) and controls (52.0 years; SD = 12.2). As can be seen, the broad age categorization, the sex, race and marital status did not affect the comparability of cases and controls. An overview of the demographic characteristics of the NHL case–control sets is in Table 1.

Table 1: Sociodemographic characteristics of the participants in a case-control study, RJ, Brazil, 2013–2016.

**Characteristic**	**Controls (n = 452)**		**Cases (n = 214)**		**p-value**
	n	%	n	%	
**Year of the interview**					0.058
2013	116	25.7	58	27.1	
2014	90	19.9	56	26.2	
2015	163	36.1	76	35.5	
2016	83	18.34	24	11.2	
**Sex**					0.135
Male	207	45.8	112	52.3	
Female	245	54.2	102	47.7	
**Age**					0.063
20 – 39	85	18.8	33	15.3	
40 – 59	235	52.0	99	46.1	
≥ 60	132	29.2	82	38.6	
**Region within the state of Rio de Janeiro**					**< 0.001**
Metropolitan	426	94.2	171	79.9	
Others	26	5.8	43	20.1	
**Time of residence (Rio de Janeiro State)**					0.671
≤ 20	267	59.5	103	57.8	
> 20	182	40.5	90	42.2	
~~No information~~	3		1		
**Education**					**< 0.001**
No degree of education or primary school	124	27.5	101	47.1	
High school	194	43.0	81	37.9	
University degree	133	29.5	32	15.0	
~~No information~~	1				
**Employment (actual)**					
Private institution (yes)	111	24.5	25	11.7	**< 0.001**
Retired (yes)	97	21.5	61	28.6	**0.042**
Self-employed (yes)	96	21.2	21	9.9	**0.001**
Public Institution (yes)	71	15.7	14	6.6	**0.001**
Not employed (yes)	63	13.9	25	11.6	0,422
Dependent (yes)	16	3.5	11	5.1	0,322
Pensioner (yes)	13	2.8	14	6.5	0.023
Sick leave (yes)	7	1.5	59	27.4	**< 0.001**
**Marital status**					0.075
No companion	125	27.7	73	34.2	
With companion	309	68.5	129	60.2	
Widowed	17	3.8	12	5.6	
~~No information~~	1		0		
**Family income (Minimum salaries)**					**< 0.001**
Mean (Std. Dev)		~~4.1 (5.9)~~		~~2.7 (3.6)~~	

Values correspond to the absolute frequencies of the variables.
The p-value is related to the comparison of variable proportions between NHL cases and controls, using the chi-square or Fisher test (for strata with absolute number < 5).
The p-value corresponds to the comparison of variable means between NHL cases and controls using the Mann-Whitney test
In bold, values with a statistically significant result (< 0.05). 1 minimum salary in Brazil in 2016 was equal to 880 reais (220 USD).
* Missing values cases = 1

Table 2 elucidates that there was no statistical difference in smoking status ($p = 0.074$) between the two groups, although the smoking burden was higher in the cases than in the controls (> 20 pack years: 26.3% vs 12.3%, $p < 0.001$). Current alcohol consumption (1 to 5 drinks per week) was lower in the cases than in the controls (26.8% vs 40.0%, $p = 0.003$). Use of cell phones and wireless phones was more frequent and more prolonged among the controls than the cases ($p < 0.001$). Regarding previous diseases, the cases of NHL presented a higher percentage of diabetes (15.1% vs 5.7%, $p < 0.001$) and diagnosis of Human T-lymphotropic virus infection (2.9% vs 0.2%, $p = 0.005$) than the controls.

Table 2: Habits and past illnesses of the participants in a case-control study, RJ, Brazil, 2013-2016.

Characteristic	Controls (n = 452) n	%	Cases (n = 214) n	%	p-value
**Smoking status**					0.074
Never smoked	283	65.5	117	57.1	
Current smoker	46	10.6	20	9.8	
Past smoker (≤ 10 years)	39	9.0	30	14.6	
Past smoker (10+ years)	64	14.9	38	18.5	
No information	20		9		
**Smoke load (Pack years)**					**< 0.001**
~~Never~~	~~283~~	~~65.7~~	~~117~~	~~57.1~~	
≤ 20	93	21.5	34	16.6	
> 20	53	12.3	54	26.3	
~~No information~~	23		9		
**Alcohol consumption (current)**					**0.003**
Non-drinker	204	46.9	120	60.6	
1 to 5 drinks per week	174	40.0	53	26.8	
> 5 drinks per week	57	13.1	25	12.6	
~~No information~~	17		16		
**~~Cellular~~ use**					**< 0.001**
Never	22	4.9	27	13.5	
Daily (≤ 10 years)	144	32.1	50	24.0	
Daily (10+years)	224	49.9	86	41.3	
Others	59	13.3	44	21.2	
~~No information~~	3		7		
**Wireless Phone use**					**< 0.001**
Never	152	34.0	106	52.2	
Daily (< 10 years)	61	13.6	25	12.2	
Daily (10+years)	94	21.1	30	14.6	
Others	140	31.3	43	21.0	
~~No information~~	5		10		
**Past Illnesses**					
Dengue	145	32.2	68	33.0	0.841
Pneumonia	57	12.8	34	16.7	0.187
HHV8 infection (Herpesvirus-8)	53	11.8	34	16.7	0.097
Asma	45	10.1	14	6.8	0.183
H. pylori infection	38	8.4	15	7.3	0.610
Diabetes	26	5.7	31	15.2	**< 0.001**
Tuberculosis	11	2.7	2	1.0	0.246
HVC infection (Hepatitis C virus)	9	2.0	5	2.5	0.773
Malaria	1	0.2	3	1.4	0.094
HTLV infection	1	0.2	6	2.9	**0.005**
EBV infection (Epstein-Barr virus)	1	0.2	2	1.0	0.233

Values correspond to the absolute frequencies of the variables.
The p-value is related to the comparison of variable proportions between NHL cases and controls, using the chi-square or Fisher test (for strata with absolute number < 5).
The p-value corresponds to the comparison of variable means between NHL cases and controls using the Mann-Whitney test
In bold, values with a statistically significant result (< 0.05).
*Missing values: controls = 2; cases = 8

Table 3 provides the results from multivariate unconditional logistic regression of occupational exposure to chemical, biological, and physical agents as well as NHL development and histological subtypes. The reference group consisted of individuals who were not occupationally exposed to these substances/agents.

Exposure to any solvents and pesticides was significantly associated with an increased risk of B- and T-cell lymphoma. We observed a positive dose-response relationship between years of exposure to these substances and increased risk for total NHL.

For occupational exposure to any solvents, hydrocarbons solvents and pesticides, the chance of developing total NHL was 2.12, 1.91 and 2.70 times greater, respectively, in the cases exposed for more than 10 years, compared to the controls (OR = 2.12; 95%CI 1.30–3.48; OR = 1.91; 95%CI 1.14–3.21; OR = 2.70; 95%CI 1.01–7.21).

B-cell lymphoma was significantly associated with occupational exposure to any of those solvents (> 10 years: OR = 1.92; 95%CI 1.15–3.21) and hydrocarbon solvents (> 10 years: OR = 1.73; 95%CI 1.01–2.96).

Risk estimates were stronger for T-cell lymphomas, but wide confidence intervals suggest caution in interpreting them. The chance of developing T-cell lymphomas was 3.58 times greater in the cases exposed for more than 10 years compared to the controls (any solvents > 10 years: OR = 3.58; 95%CI 1.22–10.48).

Occupational exposure to pesticides was significantly associated with an increased risk of total NHL, follicular lymphoma and T-cell lymphoma. We observed a dose-response association between years of exposure to these substances and increased risk of total NHL (pesticides>10 years: OR = 2.70; 95%CI 1.01–7.21). Strong associations were found between the development of T-cell lymphoma and exposure to pesticides in the workplace. The

chance of developing T-cell lymphomas was 10.65 times greater in the cases exposed to pesticides for more than 10 years, compared to the controls (pesticides>10 years: OR=10.65; 95%CI 2.35-48.24), The strongest association was observed with risk of T-cell lymphoma, although the small number of subjects suggests caution in the interpretation, due a large confidence interval.

Work with meat and meat products increased 4-fold the risk of total NHL, B-cell lymphoma, and Diffuse large B-cell lymphoma (DLBCL) subtypes (> 10 years: OR=4.19 95%CI 1.22–13.85; OR=3.29 95%CI 1.06–10.21; OR=4.73 95%CI 1.37–16.73, respectively). The small number of subjects also suggests caution in the interpretation.

We observed that the chance of developing B-cell lymphoma was 2.47 times higher in the cases exposed to sunlight for more than 10 years, compared to the controls (OR = 2.47 95%CI 1.21–5.04). Overall, the chance that those occupationally exposed for at least 10 years to sunlight had NHL was more than double compared to the controls. Follicular lymphoma was significantly associated with occupational exposure to sunlight (> 10 years: OR = 4.49 95%CI 1.40–14.36).

No statistically significant associations were found between some exposures at work (dyes, sanitizing products, biological material, and industrial radiation) and NHL.

Table 3: Occupational risk factors and development of Total NHL, B-cell lymphoma, DLBCL, follicular lymphoma and T-cell lymphoma in the participants in a hospital-based case-control study, RJ, Brazil, 2013-2016.

Risk Factors	Total NHL		B-cell lymphoma		DLBCL		Follicular lymphoma		T-cell lymphoma	
	Controls/ Cases	OR adjusted 95% CI	Controls/ Cases	OR adjusted 95% CI	Controls/ Cases	OR adjusted 95% CI	Controls/ Cases	OR adjusted 95% CI	Controls/ Cases	OR adjusted 95% CI
Chemical										
**Any solvents**										
Never	275/93	1.0	275/83	1.0	275/38	1.0	275/15	1.0	275/10	1.0
≤ 10 years	79/51	**1.70 1.04 to 2.79**[a]	79/43	1.51 0.9 to 2.55[e]	79/18	**1.94 1.01 to 3.73**[g]	79/12	**3.43 1.40 to 8.39**[j]	79/8	**3.42 1.17 to 9.99**[j]
10^{+}years	77/54	**2.12 1.30 to 3.48**[a]	77/47	**1.92 1.15 to 3.21**[e]	77/19	1.64 0.88 to 3.09[g]	77/11	**3.03 1.25 to 7.31**[j]	77/7	**3.58 1.22 to 10.48**[j]
**p trend**		< 0.001		< 0.001		0.031		0.028		0.023
**Dyes**										
Never	402/179	1.0	402/157	-	402/69	-	402/34	-	402/22	-
≤ 10 years	14/9	1.26 0.45 to 3.48[a]	14/7	-	14/3	-	14/1	-	14/2	-
10^{+}years	15/10	1.60 0.61 to 4.21[a]	15/9	-	15/3	-	15/3	-	15/1	-
**p trend**		0.208								
**Hydrocarbon solvents**										
Never	297/107	1.0	297/93	1.0	297/43	1.0	297/18	1.0	297/14	1.0
≤ 10 years	66/43	1.65 0.99 to 2.76[a]	66/38	1.57 0.91 to 2.71[e]	66/16	1.87 0.96 to 3.68[g]	66/10	**2.84 1.15 to 7.00**[k]	66/5	1.66 0.54 to 5.08[j]
10^{+}years	68/49	**1.91 1.14 to 3.21**[a]	68/42	**1.73 1.01 to 2.96**[e]	68/16	1.40 0.73 to 2.70[g]	68/10	**2.70 1.12 to 6.48**[k]	68/6	2.47 0.86 to 7.08[j]
**p trend**		< 0.001		< 0.001		0.056		0.053		0.169
**Metals**										
Never	403/179	1.0	403/158	-	403/69	-	403/35	-	403/21	1.0
≤ 10 years	13/10	1.92 0.65 to 5.72[b]	13/7	-	13/2	-	13/2	-	13/3	3.36 0.77 to 14.71[n]
10^{+}years	15/9	1.22 0.42 to 3.52[b]	15/8	-	15/4	-	15/1	-	15/1	1.47 0.17 to 12.54[n]
**p trend**		0.176								0.082

**Pesticides**										
Never	390/156	1.0	390/137	1.0	390/59	1.0	390/28	1.0	390/19	1.0
≤ 10 years	20/19	**2.41 1.13 to 5.13**[a]	20/16	2.15 1.0.96 to 4.84[f]	20/5	1.76 0.60 to 5.14[g]	20/8	**4.38 1.61 to 11.93**[j]	20/3	**4.04 1.04 to 15.69**[i]
10^{+}years	10/13	**2.70 1.01 to 7.21**[a]	10/10	2.38 0.84 to 6.77[f]	10/5	2.82 0.86 to 9.19[g]	10/2	3.05 0.56 to 16.78[j]	10/3	**10.65 2.35 to 48.24**[i]
**p trend**		**< 0.001**		0.001		0.035		< 0.001		**0.002**

**Risk Factors**	**Total NHL**		**B-cell lymphoma**		**DLBCL**		**Follicular lymphoma**		**T-cell lymphoma**	
	**Controls/ Cases**	**OR adjusted 95% CI**	**Controls/ Cases**	**OR adjusted 95% CI**	**Controls/ Cases**	**OR adjusted 95% CI**	**Controls/ Cases**	**OR adjusted 95% CI**	**Controls/ Cases**	**OR adjusted 95% CI**
**Sanitizing products**										
Never	242/95	1.0	242/82	1.0	242/37	1.0	242/16	1.0	242/13	-
≤ 10 years	66/40	1.15 0.65 to 2.02[c]	66/34	1.17 0.65 to 2.14[f]	66/15	1.73 0.86 to 3.52[i]	66/9	2.00 0.80 to 5.02[l]	66/6	-
10^{+}years	62/49	1.41 0.80 to 2.49[c]	62/43	1.47 0.81 to 2.66[f]	62/17	1.76 0.88 to 3.53[i]	62/11	2.21 0.90 to 5.41[l]	62/6	-
**p trend**		0.001		0.001		0.053		0.052		
Biological										
**Biological material**										
Never	403/187	1.0	403/164	-	403/71	-	403/37	-	403/23	-
≤ 10 years	19/7	0.35 0.10 to 1.25[b]	19/6	-	19/3	-	19/1	-	19/1	-
10^{+}years	9/4	0.78 0.20 to 2.96[b]	9/3	-	9/1	-	9/0	-	9/1	-
**p trend**		0.656								
**Meat and meat products**										
Never	417/175	1.0	417/152	1.0	417/66	1.0	417/33	**1.0**	417/23	1.0
≤ 10 years	6/11	**4.16 1.33 to 13.06**[b]	6/10	**4.35 1.38 to 13.67**[e]	6/3	3.12 0.69 to 14.19[h]	6/4	**13.07 2.79 to 61.12**[j]	6/1	3.64 0.39 to 33.99[j]
10^{+}years	8/12	**4.19 1.22 to 13.85**[b]	8/10	**3.29 1.06 to 10.21**[e]	8/6	**4.73 1.37 to 16.73**[h]	8/1	4.27 0.47 to 38.48[j]	8/1	2.92 0.33 to 25.77[j]
**p trend**		**< 0.001**		**< 0.001**		**0.001**		0.156		0.212

Risk Factors	Total NHL		B-cell lymphoma		DLBCL		Follicular lymphoma		T-cell lymphoma	
	**Controls/ Cases**	**OR adjusted 95% CI**	**Controls/ Cases**	**OR adjusted 95% CI**	**Controls/ Cases**	**OR adjusted 95% CI**	**Controls/ Cases**	**OR adjusted 95% CI**	**Controls/ Cases**	**OR adjusted 95% CI**
Physical										
**Industrial radiation**										
Never	411/191	1.0	411/167	-	411/72	-	411/37	-	411/24	-
≤ 10 years	11/4	1.04 0.29 to 3.70[d]	11/4	-	11/2	-	11/1	-	11/0	-
10^{+}years	9/3	0.28 0.05 to 1.52[d]	9/2	-	9/1	-	9/0	-	9/1	-
**p trend**		0.525								
**Sunlight (occupational)**										
Never	354/136	1.0	353/116	1.0	353/51	1.0	353/24	1.0	353/20	-
≤ 10 years	56/36	1.51 0.86 to 2.63[a]	56/32	**1.80 1.02 to 3.22[e]**	56/15	**2.13 1.02 to 4.47[h]**	56/9	**4.14 1.64 to 10.45[m]**	56/4	-
10^{+}years	21/26	1.91 0.93 to 3.90[a]	21/25	**2.47 1.21 to 5.04**[e]	21/9	2.23 0.90 to 5.58[h]	21/5	**4.49 1.40 to 14.36[m]**	21/1	**-**
**p trend**		< 0.001		**< 0.001**		0.003		**0.085**		

Total NHL: Total Non-Hodgkin's Lymphoma; DLBCL: Diffuse large B-cell lymphoma; OR: odds ratio; CI: confidence interval.
The data shows missing data.
Any solvents: turpentine, tar, asphalt, benzene, benzine, cleaners or degreaser, synthetic dye hardener, formaldehyde, gasoline, used motor oil, diesel oil, lubricating oil, crude oil, tar, kerosene, reducing agent (not specific), remover (not specs), thinner.
Hydrocarbon solvents: tar, benzene, gasoline, kerosene, thinner (not spec), remover (not spec), thinner.
Metals: chrome, iron, and lead
Pesticides: Pesticides, insecticide, rodenticide, ant killer
[a] OR adjusted for sex, age, place of residence, household income, birthplace, retired, diabetes, cell phone use, wireless phone use, alcohol consumption, HHV8 infection.
[b] OR adjusted for sex, age, place of residence, household income, birthplace, retired, diabetes, cell phone use, wireless phone use, alcohol consumption, HHV8 infection, smoking.
[c] OR adjusted for sex, age, place of residence, household income, birthplace, retired, diabetes, cell phone use, wireless phone use, alcohol consumption.
[d] OR adjusted for sex, age, place of residence, household income, birthplace, retired, diabetes, cell phone use, wireless phone use, HHV8 infection.
[e] OR adjusted for sex, age, place of residence, household income, birthplace, retired, diabetes, cell phone use.

[f] OR adjusted for sex, age, place of residence, household income, birthplace, retired, diabetes, cell phone use, wireless phone use.
[g] OR adjusted for sex, age, place of residence, place of birth.
[h] OR adjusted for sex, age, place of residence, household income, place of birth.
[i] OR adjusted for sex, age, place of birth.
[j] OR adjusted for sex, age, place of residence, diabetes, year of the interview, HHV8 infection.
[k] OR adjusted for sex, age, place of residence, diabetes, year of the interview.
[l] OR adjusted for sex, age, place of residence, year of the interview.
[m] OR adjusted for sex, age, place of residence, diabetes.
[n] OR adjusted for sex, age, retired, cell phone use, alcohol consumption, HHV8 infection.
The categories with models not estimated in the table refer to bivariate analyzes with $p>0.25$.

Ocupações que mais representam a exposição à exposição a materiais biológicos (Enfermeiro, técnico de enfermagem).

Ocupações que mais representam a exposição à radiação solar (comerciante, doméstica, do lar, eletricista, motorista, pedreiro, pintor, policial militar, segurança, vendedor).

DISCUSSION

In this study, we found an association between NHL and the following occupational exposures; any solvents, hydrocarbons, pesticides, and sunlight, when compared to those not occupationally exposed to these factors. These associations were maintained even after adjusting for variables that could confound them (described at the bottom of Table 3).

A significant upward trend with years of exposure was detected for solvents and hydrocarbon solvents (NHL (any subtype), B-cell lymphoma, and T-cell lymphoma), pesticides (NHL (any subtype), B-cell lymphoma, DLBCL, and T-cell lymphoma, sunlight (NHL (any subtype) and DLBCL), and sunlight (B-cell lymphoma, DLBCL and follicular lymphoma. No associations were found between NHL and exposure to paints, metals, cleaning products, infectious agents, industrial radiation (any sub-types).

Most of the studied NHL cases were diagnosed as B-cell lymphoma, with a higher incidence of diffuse large B-cell lymphoma (DLBCL) (82; 38.1%) and follicular lymphoma (40; 18.6%). This corresponds to the incidences found in the literature for the different histological subtypes of NHL, which are the high-grade and aggressive subtypes [1,16].

Like the acquired human immunodeficiency virus (HIV), the Human T-cell lymphotropic virus (HTLV) is a lymphotropic retrovirus, with a predilection for CD4+ T lymphocytes. Transmission occurs through biological fluids such as blood, vaginal fluid, breast milk, and semen . An estimated 5 to 200 million people have been infected with HTLV worldwide [29], and the Brazilian Ministry of Health estimates that between 700,000 and 2 million are infected there, making Brazil the country with the highest number of cases [30] . In our study, 6 (2.9%) of the cases reported being HTLV virus carriers and only 1 (0.2%) of the controls was infected. Statistical analysis demonstrated a predisposition to

develop NHL in individuals infected with HTLV (p-value = 0.005). Most HTLV carriers are asymptomatic and only about 5% of those infected will develop some type of complication. Several diseases associated with HTLV, including neurological, psychiatric, ophthalmological, dermatological, and lymph proliferative manifestations [30] . HTLV-1 is the first oncogenic retrovirus in humans [31] and is considered the etiologic agent of adult T-cell leukemia/lymphoma (ATLL), which can have a broad clinical presentation, ranging from indolent to aggressive. Although the genetic material of HTLV-2 is 70% identical to HTLV-1, it has not yet been related to lymph proliferative diseases. A limitation of the study related to this variable is that laboratory tests were not performed on controls to confirm the presence or absence of HTLV.

Hepatitis C virus (HCV) is a hepatotropic RNA virus transmitted through blood, either by transfusions, sharing needles or biological accidents. Clinical manifestations include acute hepatitis, chronic hepatitis, liver cirrhosis, and autoimmune diseases. A recent meta-analysis performed in USA has shown that HCV is a significant risk factor for NHL, with an odds ratio of 2.6 compared to the non-HCV population [32]. Despite the results found, our study did not find a positive association between contamination by HCV and the risk of developing NHL, with only 5 (2.5%) of the patients being infected, in contrast to 9 (2%) of the controls.

Among the categories of solvents present in the occupational exposure questionnaire questions, benzene was one of the chemical agents most reported by study participants. In Brazil, according to Corrêa et al (2016) [27], 7.376,761 (8.5%) workers belong to occupational groups potentially exposed to benzene, while 770,212 workers are considered definitively exposed to benzene. Occupational exposure to benzene and other aromatic

hydrocarbons and the possibility of developing NHL have been investigated in other works. An Italian study by Miligi et al. [28], which evaluated 1428 cases of NHL and 1530 controls in eight areas of Italy, through individual interviews and questionnaires, found a positive association for exposure to benzene (OR: 1.60; 95%CI 1.0–2.4) and aromatic hydrocarbons (OR: 1.20; 95%CI 0.9–1.7) between the cases. A meta-analysis found in 22 studies of benzene exposure a 22% increased risk of NHL (OR: 1.22; 95%CI 1.02–1.47). When the estimate was calculated excluding the based studies only in the self-reported occupational history, with 6 studies remaining, the risk was approximately 2 times higher for NHL (OR: 2.12, 95%CI 1.11–4.02) [29]. Other aromatic hydrocarbon solvents (styrene, BTX, toluene, xylene, and others) have been associated with NHL [30–32]. The main mechanism of carcinogenicity is reported to be the genotoxic effect. It is worth mentioning that workers are rarely exposed to a single agent, since workplaces containing benzene, also expose them to a complex mixture of other aromatic hydrocarbons, such as toluene, xylene, ethylbenzene; as well as other hydrocarbons derived from petroleum during the complex processes arising from petroleum refining, the production of coke, oils and naphtha [33].

The wide presence of heavy metals in occupational, industrial, and agricultural environments leads to increased occupational exposure and concern about their health effects [34]. In the present study, no association was found between metal exposure and overall NHL or its subtypes, although a significant odds ratio was observed for overall NHL (OR= 1.92 CI: 0.65-5.72 for those exposed <= 10 years) and for follicular lymphoma (OR= 1.22 CI: 0.42-3.52 for those exposed >= 10 years), albeit without statistical significance. The same was observed for follicular lymphoma (OR= 3.36 CI: 0.77-14.71 for those exposed <=10 years and OR= 1.47 CI: 0.17-12.54 for those exposed >= 10 years), also without statistical

significance. This result is consistent with a study conducted in Australia that similarly assessed the risk of developing NHL in individuals with occupational exposure to metal dust, which did not show a positive association[35], but Kelly et al [36] conducted a study in Italy and Sweden that observed a positive association in women between heavy metal levels in the blood and the risk of developing NHL. Further studies are needed to clarify this association found in this study.

The wide presence of heavy metals in occupational, industrial, and agricultural environments leads to increased occupational exposure and concern about their health effects [40]. In the present study, exposure to heavy metals increased five-fold the risk of developing T-cell lymphoma. This result is in line with a multicenter study by Kelly et al [41] conducted in Italy and Sweden that observed a positive association in women between heavy metal levels in the blood and the risk of developing NHL. Another study in Australia that assessed the risk of developing NHL in individuals with occupational exposure to metal dust did not demonstrate a positive association [42]. Several metals, such as lead and cadmium, are classified as carcinogens, and the hematopoietic system is one of the targets, as alterations in immune function were found [40]. Metal-induced carcinogenesis depends on the level and duration of exposure, in addition to the individual variation in the response of the organism [41]. Data in the literature are still inconsistent and scarce; nevertheless, the results of this study found a positive association between exposure to metals and the development of certain types of NHL. Subsequent studies are needed to clarify this association.

Case-control, cohort, and meta-analysis studies have explored the association between exposure to active ingredients of specific pesticides and the NHL development [37]. The IARC classified some pesticides as exhibiting carcinogenic potential after observing a

positive association between pesticides exposure and NHL development. DDT, diazinon, glyphosate, and malathion have been classified as probably carcinogenic to humans (group 2A) [24]. Furthermore, 2,4-D, chlordane, heptachlor, hexachlorobenzene, lindane, mirex, and pentachlorophenol were classified as possibly carcinogens to humans (group 2B) [24]. Glyphosate and 2,4-D are among the most used pesticides in Brazilian crops. Leon et al. [38] identified an association between NHL and occupational exposure to pesticides in cohorts from France (Agriculture and Cancer – AGRICAN); Norway (Cancer in the Norwegian Agricultural Population – CNAP); and the USA (Agriculture Health Study – AHS). For most of the chemical groups in the pesticides evaluated, no associations were observed in 33 active ingredients. However, increased total NHL risk was observed in farmers who used terbufos; CCL/SLL among those who used deltamethrin; and DLBCL among those who used glyphosate. It should be clarified that terbufos is an extremely toxic organophosphate insecticide; deltramethrin is an insecticide of the pyrethroid group, and glyphosate is an herbicide. All three are widely used in Brazil, including in unauthorized cultures.

The results of this study also showed the association of exposure to meat and meat products with an increased chance of developing NHL. These results corroborate the results found in the case-control study conducted in eight Canadian provinces by Fritschi et al. [39], who reported an increased risk between exposure to beef cattle and NHL; as well as by Tranah et al [40] who observed an increased risk of NHL in individuals who lived or worked with cattle (1.6, CI=1.0–2.5) and pigs (OR = 1.5; CI=1.1–2.1). With exposure to pigs for ≥ 5 years, the increased risk also occurred for NHL (OR = 1.8; CI=1.2–2.6) and for diffuse large-cell and immunoblastic large-cell lymphoma (DLCL) (OR = 2.0, CI=1.2-3.4). According to the authors, contact with infectious agents as a result of work with livestock or contact and

handling of pork and beef increases the risk of NHL, as they can carry lymphomagenic infectious agents. Freeman et al. [41] analyzing data from the Agricultural Health Study, observed increased risk of NHL (RR = 1.6, 95%CI1.0–2.4) among those who raised poultry and those who were veterinarians on poultry farms (RR = 12.2; 95%CI:1.6–96.3) [40].

In New Zealand, Mannetje et al. [7] observed a 2-fold higher risk of NHL in workers who handled meat, especially those who worked at meat processing plants, when compared to controls. Although the studies described found some association between exposure and NHL, this association was not for all investigated animals or for all NHL subtypes. Freeman et al. [41] for example found an association for raising poultry but not for sheep; Fritschi et al. [39] found an association between NHL and DLCL for occupational exposure to beef cattle, but not to any other animal.

Our study indicated that occupational exposure to sunlight for at least 10 years doubled the chance of total NHL, B-cell lymphomas and DLBCL, compared to the controls. Lu et al. [12] in a meta-analysis with 10 case-control studies and one cohort, in the period between 1997 and 2012, observed a positive association between occupational sun exposure and NHL (OR = 1.15; 95%CI 1.05–1.23), but not for the common subtypes of non-Hodgkin lymphoma (T-cell and B-cell lymphoma, and chronic lymphocytic leukemia). Similarly, Karipidis et al. [42] observed that the group with cumulative occupational exposure and minimum exposure time of 5 years before diagnosis had a 1.46-fold higher chance of having NHL (95%CI 1.06–2.02) than the unexposed. Although the association between occupational sunlight exposure and NHL was mentioned in the studies described here, other studies have not found this association [43,44]; therefore, more research is needed.

The authors considered it important to evaluate the association between subtypes of non-Hodgkin lymphomas and occupational exposures, since few articles carried out this investigation. However, for some types of occupational exposures, such as biological material, paints, metals and industrial radiation, as the number of observations was very small, it was not possible to perform statistical analysis by subtypes of non-Hodgkin lymphoma. Therefore, only the results of the main outcome, total NHL, were evaluated. Furthermore, no signs of effect modification were observed when measures of association were compared for the strata of each variable for total NHL and for the different subtypes of non-Hodgkin's lymphoma.

Men exhibited a higher frequency of NHL cases. This demographic profile agrees with estimates of the Brazilian National Cancer Institute [6] and with the profile of cases described in the literature. The mean age of cases was 56 years old (SD = 12.7 years), with the largest portion of individuals over 45, in accordance with the InterLymph consortium [17–20]. It is noteworthy that the broad age categorization did not affect the comparability of cases and controls by gender and case-control status.

Both cases and controls reside in the metropolitan mesoregion of the Rio de Janeiro state. This data has also a limitation. As INCA is a reference hospital in cancer and one of the only ones to offer comprehensive and free treatment for cancer (public health), patients from all over the state of Rio de Janeiro want to be treated at INCA. Therefore, some individuals, from others regions, outside the metropolitan region, came to INCA. In addition, it is important to emphasize that most individuals stop working, retire, or take a leave of absence after discovering the disease, which lowers the family income. This could be one of the explanations for the lower income of the cases compared to the controls [21].

For follicular and marginal zone lymphoma, smoking is a risk factor [20,22]. The immunological effects observed in smokers, such as immunosuppression and increased leukocyte production, may be associated with the development of lymphomas [23]. In addition, cigarette smoke contains substances known to be carcinogens, including benzene and formaldehyde [24]. In a cohort study conducted in the United States with 1,926 cases of NHL, smoking (former smokers) was associated with the occurrence of NHL (histological type of T-cells) [25]. The present study found no statistical differences in smoking between the two groups, although the smoke load (pack years) was higher among the cases than the controls. It is noteworthy that many patients stop smoking after being diagnosed with cancer. Therefore, the answers given by patients when asked about being current smokers may reflect the condition of no longer smoking after the diagnosis of NHL.

No increase in alcoholic beverages consumption was observed among the cases after analyzing the information on the number of doses of alcoholic beverages consumed per week; therefore, the frequency found in the present study (61%) was lower than in other studies [26].

Limitations of the study

The authors consider the presented results to be of utmost relevance considering the history of intensive use of carcinogenic agents classified in Group 1 by IARC in the country, some of which have already been banned in other countries. It should also be noted that there is a scarcity of analytical case-control studies in Brazil that include occupational risk factors for cancer. Another important issue is the possibility of these results providing tools for occupational health surveillance in states and municipalities regarding workers' exposure to

the agents studied here. However, we acknowledge that these results should be viewed with caution, considering some problems inherent to the study design itself.

The controls were selected from the companions and visitors of patients diagnosed with solid cancers, as well as from the waiting room of the hospital's Blood Bank, which also mostly receives visitors and companions of patients. This procedure was adopted to minimize the potential selection bias of controls. Thus, the controls selected at the Blood Bank did not differ significantly from the controls recruited in the hospital wards.

The authors attempted to minimize the potential selection bias of controls by recruiting companions of hospitalized patients. As this group was predominantly composed of women, another recruitment strategy was used. During the visits of relatives, friends, and neighbors of hospitalized patients, they were routinely invited to become blood donors. Upon acceptance, they were invited to participate in the research. Only blood donors who went to visit hospitalized patients were recruited. Thus, the blood donors did not differ significantly from the controls recruited in the hospital wards. It is evident that those who agreed to donate blood may differ slightly from those who did not donate, in terms of their health status, considering that the donors are likely to be healthier individuals who are still relatives, neighbors, and/or friends. It should also be noted that INCA is not a conventional blood collection institution like a blood center.

Another issue that should be addressed as a potential limitation for the findings is the multiple comparisons involved in this study. This is an exploratory study that sought to evaluate various exposures in a single study. For this reason, important details such as occupation, economic activities, and exposures could not be assessed in a more disaggregated

manner, limiting the observation of effects in a more specific way. It was also not possible to investigate multiple simultaneous exposures.

It should also be considered as a limitation the inherent memory bias in the study design. In this case, it was further accentuated by the administration of a questionnaire to capture the recall of occupational history, which was necessary for analyzing the association between work and NHL in this study. Each patient was asked to report all occupations held in the last 20 years. Cases may have made more effort to remember possible exposures compared to controls.

It is also worth noting that the study did not include any environmental assessment or clinical evaluation of cases and controls. The only tool used was a data collection instrument, and the information was obtained through interviews. In other words, we only have self-reported exposures mentioned by the interviewees. In an attempt to observe cumulative exposure, only years of exposure to certain carcinogenic agents were considered. There was no way to measure the intensity of each exposure.

It is important to emphasize that as the study was carried out in a National Cancer Institute of the Ministry of Health of Brazil, which is a reference for cancer, and is located in the state of Rio de Janeiro, which is a very populous state in Brazil, it is assumed that the sample included in the study is representative of the general population and its results can be extrapolated to Brazil and other countries with the same socio-demographic and occupational characteristics.

CONCLUSIONS

The data from the present study support the hypothesis that occupational exposure to solvents, hydrocarbons, pesticides, metals, meat and meat products, and sunlight is related to an increased chance of developing Non-Hodgkin's Lymphoma in adults. However, occupational exposure to paints, cleaning products, human biological materials, metals and industrial radiation was not statistically associated with this type of neoplasm. The results of this study corroborate other international studies that evaluated and found an association between some occupational exposures and NHL.

However, these results must be viewed with caution, since the results were presented both by the total number of cases and by subtypes of lymphomas, reducing the number of observations in some cells during the crossing with exposure groups. This form of analysis generated measurements of great magnitude for some subtypes, but with wide confidence intervals. Therefore some without statistical significance, requiring is careful in the evaluations and conclusions. It should also be noted that the study was of an exploratory nature and that it allowed assessing which occupational risks were associated with NHL in Brazil. From this preliminary study we will be able to advance in specific exposures. In several developed countries or in countries with the same socio-demographic and occupational characteristics as Brazil, some agents evaluated here are used intensely, requiring constant monitoring and surveillance of these exposures, exposed workers, and diseases, including cancer, such as NHL. Thus, the results of the present study can contribute to and support strategies in public health and surveillance programs for workers, also will help the various institutions in the world involved in public health actions to design strategies for the prevention and control of work-related cancer.

Acknowledgments

Adriana Scheliga from the Oncology Service of the Brazilian National Cancer Institute (INCA); Employees of Hematology Service and National Tumor Bank (BNT) of the Brazilian National Cancer Institute (INCA).

Authors` contributions

MS and UO designed of the work (project); VIBC, SNF, CAA, PGO, LMV did the acquisition and analysis; VIBC and AOCS performed the statistical analyzes; MS, VIBC, UBO and RH did the interpretation of data; MS, VIBC, SNF, CAA, UBO and CGS have drafted the work or substantively revised it; MS, VIBC, SNF, CAA, PGO, LMV, AOCS, CGS and UBO have approved the submitted version.

Competing interests

The authors declare that they have no competing interests.

REFERENCES

1. Chihara D, Nastoupil LJ, Williams JN, Lee P, Koff JL, Flowers CR. New insights into the epidemiology of non-Hodgkin lymphoma and implications for therapy. Expert Rev Anticancer Ther. 2015;15: 531–544. doi:10.1586/14737140.2015.1023712
2. Vincent T. DeVita, Samuel Hellman SAR. Cancer: Principles and Practice of Oncology. 6th editio. Clinical Trials. 2018.
3. Morton LM, Wang SS, Devesa SS, Hartge P, Weisenburger DD, Linet MS. Lymphoma incidence patterns by WHO subtype in the United States, 1992-2001. Blood. 2006;107: 265–276. doi:10.1182/blood-2005-06-2508
4. Zhang Y, Dai Y, Zheng T, Ma S. Risk factors of non-Hodgkin's lymphoma. Expert Opin Med Diagn. 2011;5: 539–550. doi:10.1517/17530059.2011.618185

5. GLOBOCAN 2018. GLOBOCAN 2018: counting the toll of cancer. The Lancet. Elsevier Ltd; 2018. doi:10.1016/S0140-6736(18)32252-9

6. INCA. Instituto Nacional de Cancer José Alencar Gomes da Silva. Estimativa 2016 - Incidência de Câncer no Brasil. Ministério da Saúde Inst. Nac. Cancer José Alencar Gomes da Silva. 2016. Available: https://www.inca.gov.br/campanhas/dia-nacional-de-combate-ao-cancer/2015/estimativa-2016-incidencia-de-cancer-no-brasil

7. t Mannetje A, De Roos AJ, Boffetta P, Vermeulen R, Benke G, Fritschi L, et al. Occupation and risk of non-hodgkin lymphoma and its subtypes: A pooled analysis from the interlymph consortium. Environ Health Perspect. 2016;124: 396–405. doi:10.1289/ehp.1409294

8. Morton LM, Slager SL, Cerhan JR, Wang SS, Vajdic CM, Skibola CF, et al. Etiologic heterogeneity among non-hodgkin lymphoma subtypes: The interLymph non-hodgkin lymphoma subtypes project. J Natl Cancer Inst Monogr. 2014; 130–144. doi:10.1093/jncimonographs/lgu013

9. Boffetta P. I. Epidemiology of adult non-Hodgkin lymphoma. Annals of Oncology. 2011;22: iv27–iv31. doi:10.1093/annonc/mdr167

10. Eriksson M, Hardell L, Carlberg M, Åkerman M. Pesticide exposure as risk factor for non-Hodgkin lymphoma including histopathological subgroup analysis. Int J Cancer. 2008;123: 1657–1663. doi:10.1002/ijc.23589

11. Karunanayake CP, Dosman JA, Pahwa P. Non-hodgkin's lymphoma and work in agriculture: Results of a two case-control studies in Saskatchewan, Canada. Indian J Occup Environ Med. 2013;17: 114–121. doi:10.4103/0019-5278.130860

12. Lu D, Xu F, Hu K, Yin L, Duan H, Zhang J, et al. Occupational ultraviolet exposure and risk of non-Hodgkin's lymphomas: A meta-analysis. Oncotarget. 2017;8: 62358–62370. doi:10.18632/oncotarget.18140

13. Fritschi L, Benke G, Hughes AM, Kricker A, Vajdic CM, Grulich A, et al. Risk of non-Hodgkin lymphoma associated with occupational exposure to solvents, metals, organic dusts and PCBs (Australia). Cancer Causes Control. 2005;16: 599–607. doi:10.1007/s10552-004-7845-0

14. Cocco P, Satta G, Meloni F, Pilia I, Ahmed F, Becker N, et al. Occupational exposure to organic dust and risk of lymphoma subtypes in the EPILYMPH case–control study. Scand J Work Environ Health. 2021;47: 42–51. doi:10.5271/sjweh.3925

15. Boyle P, Levin B. World Cancer Report 2008. Cancer Control. 2008. doi:10.1016/j.cma.2010.02.010

16. Araújo LH de L, Victorino APO de S, Melo AC de, Assad DX, Lima DS, Alencar DR de, et al. Linfoma Não-Hodgkin de Alto Grau - Revisão da Literatura. Revista Brasileira de Cancerologia. 2008;54: 175–183. doi:10.32635/2176-9745.rbc.2008v54n2.1747

17. Cerhan JR, Kricker A, Paltiel O, Flowers CR, Wang SS, Monnereau A, et al. Medical history, lifestyle, family history, and occupational risk factors for Diffuse Large B-cell Lymphoma: The

interLymph non-Hodgkin lymphoma subtypes project. J Natl Cancer Inst Monogr. 2014; 15–25. doi:10.1093/jncimonographs/lgu010

18. Linet MS, Vajdic CM, Morton LM, De Roos AJ, Skibola CF, Boffetta P, et al. Medical history, lifestyle, family history, and occupational risk factors for follicular lymphoma: The interlymph non-hodgkin lymphoma subtypes project. J Natl Cancer Inst Monogr. 2014; 26–40. doi:10.1093/jncimonographs/lgu006

19. Smedby KE, Sampson JN, Turner JJ, Slager SL, Maynadié M, Roman E, et al. Medical history, lifestyle, family history, and occupational risk factors for mantle cell lymphoma: The interlymph non-hodgkin lymphoma subtypes project. J Natl Cancer Inst Monogr. 2014; 76–86. doi:10.1093/jncimonographs/lgu007

20. Wang SS, Flowers CR, Kadin ME, Chang ET, Hughes AM, Ansell SM, et al. Medical history, lifestyle, family history, and occupational risk factors for peripheral T-cell lymphomas: The interlymph non-hodgkin lymphoma subtypes project. J Natl Cancer Inst Monogr. 2014; 66–75. doi:10.1093/jncimonographs/lgu012

21. INCA IN de CJAG da S. Direitos sociais da pessoa com câncer. 2020; 20.

22. Bracci PM, Benavente Y, Turner JJ, Paltiel O, Slager SL, Vajdic CM, et al. Medical history, lifestyle, family history, and occupational risk factors for marginal zone lymphoma: The interLymph non-Hodgkin lymphoma subtypes project. J Natl Cancer Inst Monogr. 2014; 52–65. doi:10.1093/jncimonographs/lgu011

23. Stämpfli MR, Anderson GP. How cigarette smoke skews immune responses to promote infection, lung disease and cancer. Nat Rev Immunol. 2009;9: 377–384. doi:10.1038/nri2530

24. Pearce N, Blair A, Vineis P, Ahrens W, Andersen A, Anto JM, et al. IARC monographs: 40 years of evaluating carcinogenic hazards to humans. Environ Health Perspect. 2015;123: 507–514. doi:10.1289/ehp.1409149

25. Diver WR, Patel A V., Thun MJ, Teras LR, Gapstur SM. The association between cigarette smoking and non-Hodgkin lymphoid neoplasms in a large US cohort study. Cancer Causes and Control. 2012;23: 1231–1240. doi:10.1007/s10552-012-0001-3

26. Luz LL. Non-Hodgkin's Lymphoma: Five-year Survival in a Historical Cohort of Patients who Underwent Stem Cell Transplantation in Brazil. Cancerologia Revista Brasileira de Cancerologia. 2014;60: 35–42.

27. Corrêa MJM, Santana VS. Exposição ocupacional ao benzeno no Brasil: Estimativas baseadas em uma matriz de exposição ocupacional. Cad Saude Publica. 2016;32: 1–11. doi:10.1590/0102-311X00129415

28. Miligi L, Costantini AS, Benvenuti A, Kriebel D, Bolejack V, Tumino R, et al. Occupational exposure to solvents and the risk of lymphomas. Epidemiology. 2006;17: 552–561. doi:10.1097/01.ede.0000231279.30988.4d

29. Steinmaus C, Smith AH, Jones RM, Smith MT. Meta-analysis of benzene exposure and non-Hodgkin lymphoma: Biases could mask an important association. Occup Environ Med. 2008;65: 371–378. doi:10.1136/oem.2007.036913

30. Bassig BA, Friesen MC, Vermeulen R, Shu XO, Purdue MP, Stewart PA, et al. Occupational exposure to benzene and non-hodgkin lymphoma in a population-based cohort: The Shanghai women's health study. Environ Health Perspect. 2015;123: 971–977. doi:10.1289/ehp.1408307

31. Cocco P, T'Mannetje A, Fadda D, Melis M, Becker N, De Sanjosé S, et al. Occupational exposure to solvents and risk of lymphoma subtypes: Results from the Epilymph case-control study. Occup Environ Med. 2010;67: 341–347. doi:10.1136/oem.2009.046839

32. Mehlman MA. Causal relationship between non-Hodgkin's lymphoma and exposure to benzene and benzene-containing solvents. Ann N Y Acad Sci. 2006;1076: 120–128. doi:10.1196/annals.1371.004

33. SEFAZ S da F do E da B. MANUAL DE TRIBUTAÇÃO: PETRÓLEO, GÁS NATURAL, NAFTA PETROQUÍMICA, COMBUSTÍVEIS E LUBRIFICANTES. 2016.

34. Deubler EL, Gapstur SM, Diver WR, Gaudet MM, Hodge JM, Stevens VL, et al. Erythrocyte levels of cadmium and lead and risk of B-cell non-Hodgkin lymphoma and multiple myeloma. Int J Cancer. 2020;147: 3110–3118. doi:10.1002/ijc.33136

35. Fritschi L, Benke G, Hughes AM, Kricker A, Vajdic CM, Grulich A, et al. Risk of non-Hodgkin lymphoma associated with occupational exposure to solvents, metals, organic dusts and PCBs (Australia). Cancer Causes and Control. 2005;16: 599–607. doi:10.1007/s10552-004-7845-0

36. Kelly RS, Lundh T, Porta M, Bergdahl IA, Palli D, Johansson AS, et al. Blood erythrocyte concentrations of cadmium and lead and the risk of B-cell non-Hodgkin's lymphoma and multiple myeloma: A nested case-control study. PLoS One. 2013;8. doi:10.1371/journal.pone.0081892

37. Schinasi L, Leon ME. Non-hodgkin lymphoma and occupational exposure to agricultural pesticide chemical groups and active ingredients: A systematic review and meta-analysis. Int J Environ Res Public Health. 2014;11: 4449–4527. doi:10.3390/ijerph110404449

38. Leon ME, Schinasi LH, Lebailly P, Beane Freeman LE, Nordby KC, Ferro G, et al. Pesticide use and risk of non-Hodgkin lymphoid malignancies in agricultural cohorts from France, Norway and the USA: A pooled analysis from the AGRICOH consortium. Int J Epidemiol. 2019;48: 1519–1535. doi:10.1093/ije/dyz017

39. Fritschi L, Johnson KC, Kliewer E V., Fry R. Animal-related occupations and the risk of leukemia, myeloma, and non-Hodgkin's lymphoma in Canada. Cancer Causes and Control. 2002;13: 563–571. doi:10.1023/A:1016331128897

40. Tranah GJ, Bracci PM, Holly EA. Domestic and farm-animal exposures and risk of non-Hodgkin's lymphoma in a population-based study in the San Francisco Bay Area. Cancer

Epidemiology Biomarkers and Prevention. 2008;17: 2382–2387. doi:10.1158/1055-9965.EPI-08-0373

41. Beane Freeman LE, DeRoos AJ, Koutros S, Blair A, Ward MH, Alavanja M, et al. Poultry and livestock exposure and cancer risk among farmers in the agricultural health study. Cancer Causes and Control. 2012;23: 663–670. doi:10.1007/s10552-012-9921-1

42. Karipidis KK, Benke G, Sim MR, Kauppinen T, Kricker A, Hughes AM, et al. Occupational exposure to ionizing and non-ionizing radiation and risk of non-Hodgkin lymphoma. Int Arch Occup Environ Health. 2007;80: 663–670. doi:10.1007/s00420-007-0177-0

43. Tavani A, Bosetti C, Franceschi S, Talamini R, Negri E, La Vecchia C. Occupational exposure to ultraviolet radiation and risk of non-Hodgkin lymphoma. European Journal of Cancer Prevention. 2006;15: 453–457. doi:10.1097/00008469-200610000-00011

44. Boffetta P, van der Hel O, Kricker A, Nieters A, fe Sanjosé S, Maynadié M, et al. Exposure to ultraviolet radiation and risk of malignant lymphoma and multiple myeloma - A multicentre European case - Control study. Int J Epidemiol. 2008;37: 1080–1094. doi:10.1093/ije/dyn092

Figure

Click here to access/download;Figure;March_17_2023_vF Fig 1. Flow Diagram_Research Participants.docx

Fig 1. Research participants flow diagram

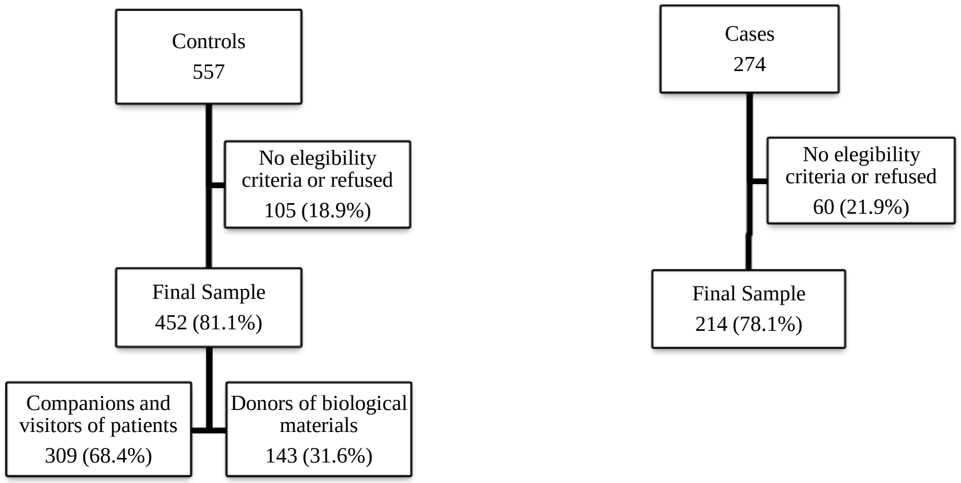

INVESTIGATION OF OCCUPATIONAL RISK FACTORS FOR THE DEVELOPMENT OF NON-HODGKIN LYMPHOMA IN ADULTS: A HOSPITAL-BASED CASE-CONTROL STUDY

Marcia Sarpa[1,2,*], Vanessa Índio do Brasil da Costa[1], Sâmila Natiane Ferreira[1,2], Carolina Ávila de Almeida[1,2], Paula Gabriela Sousa de Oliveira[1,2], Letícia Vargas de Mesquita[1,2], Arthur OC Schilithz[1], Claudio Gustavo Stefanoff[3], Rocio Hassan[4#], Ubirani Barros Otero[1].

[1]Coordination of Prevention and Surveillance, Brazilian National Cancer Institute. Rua Marquês de Pombal, 125 – Centro, Rio de Janeiro – Brazil. CEP: 20230-240. Phone: +55 (21) 3207-6089.

[2]Environmental Mutagenesis Laboratory, Federal University of Rio de Janeiro State (UNIRIO). Rua Frei Caneca, 94 – Centro, Rio de Janeiro – Brazil. CEP 20211-040. Phone: +55 (21) 2531-7998.

[3]Coordination of Clinical Research and Technological Incorporation, Brazilian National Cancer Institute. Rua André Cavalcanti 37, 2° andar – Bairro de Fátima, Rio de Janeiro, RJ – Brazil. CEP: 20231-050. Phone: +55 (21) 3207-6541.

[4]Oncovirology Laboratory. Bone Marrow Transplantation Center. Brazilian National Cancer Institute (INCA). Praça da Cruz Vermelha, 25 – Centro, Rio de Janeiro – Brazil. CEP: 20230-240. Phone: +55 (21) 3207-1000

[#]in memorium

***Corresponding Author:** Marcia Sarpa.

[1]Institution: Coordination of Prevention and Surveillance. Brazilian National Cancer Institute José Alencar Gomes da Silva. Adress: Rua Marquês de Pombal, 125, 5°andar – Centro, Rio de Janeiro – Brazil. Zipcode: 20230-240. Phone: +55(21)3207-6092. E-mails: mmello@inca.gov.br; marciasarpa@gmail.com

ABSTRACT

Non-Hodgkin's Lymphoma (NHL) is a malignancy of the lymphoid lineage of the hematopoietic system and has increased worldwide, especially in developed countries. Better diagnostic and recording techniques, longer life expectancy, and greater exposure to risk factors are hypotheses for this growing incidence curve. Occupational exposures to chemical, biological, and physical agents have also been associated with NHL development, but the results are still controversial. ~~This study described the profile and the past illnesses of the target population, and estimated the association between chemical, biological, and physical risk factors from occupational exposure and the occurrence of NHL in Brazil. This hospital-based observational case-control study was developed at the Cancer Hospital I (HCI), Brazilian National Cancer Institute (INCA), Brazil, between 2013 and 2016. The 214 adult patients were diagnosed as incident cases of NHL through histopathological analysis. The control group included 452 visitors or companions of patients with non-hematologic neoplasia from the HCI/INCA~~. Socio-demographic, clinical, and occupational exposure data were obtained through individual interviews with a standardized questionnaire. Clinical, laboratory, and histopathological data were obtained through medical records. Associations were found between risk of NHL *(any subtype), B-cell lymphoma, and T-cell lymphoma was elevated among the ever exposed to solvents* [give the Odds ratio and 95% confidence interval associated with ever exposure (=all the exposed)], *pesticides* [Odds ratio and 95% CI for the ever exposed], *meat and meat products* [instead of animal and visceral materials] [Odds ratio and 95% CI for the ever exposed], *and sunlight* [instead of solar radiation] [Odds ratio and 95% CI for ever exposed]. *A significant upward trend with years of exposure was detected for solvents and hydrocarbon solvents (NHL (any subtype) p-value for trend), B-cell lymphoma (p for trend), and T-*

Commented [UBO1]: We investigated the association between occupational exposures and risky lifestyle for NHL through a case-control study design with 214 adult patients and 452 population controls.

cell lymphoma (p for trend)), pesticides (NHL (any subtype), p for trend) , B-cell lymphoma (p for trend), DLBCL (p for trend), and T-cell lymphoma (p for trend), raw meat (NHL (any subtype) (p for trend) and DLBCL (p for trend), and sunlight (B-cell lymphoma (p for trend), DLBCL [? Not sure] *(p for trend), and follicular lymphoma* [if still significant after adjustment for agricultural work] *(p for trend).* ~~The project was approved by the Research Ethics Committee of the INCA under registration number 135/11.~~ Associations were found between risk of total NHL any subtypesand, any solvents (OR = 2.38; 95% CI: 1.42–4.00), hydrocarbon solvents (OR = 2.02; 95% CI: 1.17–3.49), pesticides (OR = 3.20; 95% CI: 1.15–8.90), animal and visceral material (OR = 4.67; 95% CI: 1.36–16.05) and solar radiation (OR = 2.22; 95% CI: 1.07–4.62). Furthermore, exposure to solvents and hydrocarbons solvents for more than 10 years increased the chance of developing B-cell and T-cell lymphoma. Exposure to ~~metals~~, pesticides, animal and visceral material, and solar radiation increased the chance of ~~T-cell lymphoma~~, B-cell and T-cell lymphoma, ~~B-cell lymphoma and DLBCL~~, and B-cell lymphoma, respectively. The results of this study are in agreement with other international studies, can be extrapolated to other countries that have the same socio-demographic and occupational characteristics as Brazil and will support strategies for surveillance and control of work-related cancer in the world.

Keywords: Non-Hodgkin Lymphoma; occupational risk factors; lymphoid lineage; pesticides; solvents.

INTRODUCTION

Non-Hodgkin's lymphomas (NHLs) are a group of malignant diseases with more than 40 main subtypes, andwhich have distinct genetic, morphological, and clinical characteristics [1]. They originate from progenitor cells and mature B cells, from progenitor and mature T-cells or from natural killer cells (rarely), which vary in pathophysiology, histological appearance, clinical course, and response to therapy [2]. The most common subtypes are diffuse large B-cell and follicular lymphomas, together accounting for approximately 30 and 20% of all NHL cases, respectively [3,4]. Other less common subtypes are Burkitt lymphoma (1–5% of adult lymphoma cases); lymphoplasmacytic lymphoma (rare and indolent); chronic lymphocytic leukemia/small cell lymphocytic-lymphoma; marginal zone lymphoma (5–10% of NHL cases); mantle cell lymphoma (2–4% of NHL cases); peripheral T-cell lymphoma, which the WHO defines as "natural killer" and mature T-cell neoplasms, and Mycoses fungoides/Sezary syndrome [1].

The highest incidence rates of NHL are found in industrialized countries in North America and Europe, in addition to Australia and New Zealand (> 7.0/100,000 thousand, in both men and women) [5]. Each year, an estimated 509,590 new cases of NHL (2.7% of total cancer) and around 250,000 deaths (2.4% of total deaths) occur worldwide. NHL is among the top ten most common types of cancer in Brazil, each year. For the triennium 2023-2025, an estimated 6,420 and 5,620 new cases will occur annually in Brazilian men and women, respectively. This corresponds to an estimated rate of 6.08 new cases per 100,000 men and 5.08 per 100,000 women. The highest incidence rates of NHL are observed in men in the Southeastern region (8.2/100 thousand) and in women in the Southern Region (7.3/100 thousand) [6].

The best-established risk factors for NHL are immunosuppression; infections, including HIV/AIDS virus, hepatitis C, Epstein Bar; and autoimmune diseases [7]. The proportion of NHL cases related to celiac disease, systemic lupus erythematosus (SLE), Sjogren's syndrome, and rheumatoid arthritis (RA), for example, is considered small, due to the low prevalence of these diseases in the general population [4,8,9]. Hereditary factors are also cited, but they are responsible for only a small portion of NHL cases [9]. Other factors for the development of NHL include smoking (follicular lymphoma) and occupation, especially occupational exposure to specific chemical, physical, and biological agents.

The chemical agents most consistently associated with NHL, mentioned in the literature, are solvents and pesticides [7,10,11]. Other occupational factors related to NHL include solar radiation [12]; ionizing radiation [11]; organic dust (grain, leather, fabric, and wood dusts) [13,14]; however, evidence of the association of these factors with NHL is innot yet consistent [7].

In a recent review, Micallef et al. [13], using the classification criteria of the The International Agency for Research on Cancer (IARC), classifies 87 occupational exposures in groups 1 and 2A [13]".listed 87 agents from groups 1 and 2A as occupational exposure (68 from group 1 and 19 from group 2A). Nine of these exposures were attributed were specifically associated with non-Hodgkin's lymphoma.to non-Hodgkin lymphomas, with only two considered to have sufficient evidence in humans.

Thus, the aim of the present study was to describe the socio-demographic and clinical data of patients treated at the Brazilian National Cancer Institute, in addition to assess whether there is an association between occupational exposures to chemical, physical and biological agents and the chance of developing NHL in these patients. Also, it is expected

that the results of this study support the implementation of actions to prevent and control exposure to carcinogens in the work environment.

MATERIALS AND METHODS

Study design and population

This is a hospital-based case-control study, with patients residingthat reside in the state of Rio de Janeiro (RJ, Brazil) and were seen at the Brazilian National Cancer Institute (INCA).

Commented [UBO2]: The project was approved by the Research Ethics Committee of the INCA under registration number 135/11.

It is noteworthy that 17.5 million people live in the 92 municipalities in the state of Rio de Janeiro (RJ, Brazil). Therefore, althoughdespite the case-control study was carried out in a single institute in the country, this institute is a reference hospital and is responsible for providing care to around 7.000 new enrollments patients per year. Also, it is important to emphasize that INCA is a national institute, which belongs to the Ministry of Health of the Federal Government of Brazil, is considered a reference hospital for the care of cancer patients in Brazil, being a High Complexity Assistance Centers in Oncology (CACON). INCA is a national institute, part of the Ministry of Health of the Federal Government of Brazil, responsible for providing care to around 7.000 new patients per year and and INCA is also responsible for formulating public policies on cancer in Brazil.

The sample size calculation was performed using the EpiInfo software version 3.3.2, using the calculation basis for case-control studies, for a confidence interval of 95%. Considering human exposure to the main carcinogens described in the literature associated with lymphomas, it was decided to calculate the sample size was determined using with the following parameters: $\alpha = 0.05$; case-control 2:1 ratio; prevalence of occupational hazards

among controls of 5% (adopting a more conservative measure; OR = 2.5). Thus, 215 cases and 430 controls would be required to achieve a statistical power of 80%”.

for a statistical power of 80%, it will be necessary 215 cases and 430 controls.

Inclusion criteria for the cases were: age between 25 and 75 years; residents of the state of Rio de Janeiro; Brazilian nationality; with a primary diagnosis of NHL confirmed by laboratory and histopathological tests. Exclusion criteria for the cases were: patients diagnosed with HIV were not included. Eligible controls were visitors or companions of patients at INCA/HCI, aged between 25 and 75 years and free from any type of cancer at the time of participating in the study”.

Inclusion criteria for the control group were: visitors or companions of patients at INCA/HCI, aged between 25 and 75 years, without previous or current diagnosis of any type of cancer and not accompanying or visiting patients diagnosed with hematological cancer.

During the study, 274 cases and 557 controls matched to cases by gender and age-group (± 10 years) were invited to participate in the study. Of these, 10 cases (3.6%) refused to participate in the study and 50 cases (18.2%) did not meet the previously established eligibility criteria. Regarding controls, 105Regarding controls, 105 subjects (18.9%) did not meet the eligibility criteria or refused to participate in the study. As most visitors of patients diagnosed with non-haematological neoplasms at the hospital's outpatient clinics or wards were female, it was necessary to recruit male blood donors (N = 143) at the hospital Haemotherapy Service. A total of 214 NHL cases (112 men and 102 women) and 452 controls (207 men and 245 women) were included in the study. The The complete diagram with the initial and final number of study participants, including refusals, can be seen in Figure 1.

Therefore, this study includes 214 incident patients between the ages of 25 and 75 years with NHL (International Classification of Disease - ICD10; C82-85) diagnosed between 2013 and 2016. Hospital-based cancer registries of cases were identified, and histopathological reports were examined to confirm the diagnosis of NHL, based on the WHO classification: Follicular non-Hodgkin's lymphoma (C82) N cases; Diffuse non-Hodgkin lymphoma (C83) N cases; Cutaneous and peripheral T-cell lymphomas (C84) N cases; Non-Hodgkin lymphoma of other and unspecified types (C85) N cases) [15]. Regarding the cases of NHL, the predominant histological type was B-cell lymphoma (185 cases; 86.0%) and T-cell lymphoma were 30 cases (14.0%).

~~The studied cases were approximately 2:1 matched, paired by sex and age with the control group. 452 controls without cancer history were selected from the companions and visitors of patients undergoing treatment at HCI/INCA for non-hematological neoplasms and among donors of biological materials with the same sociodemographic profile as the NHL cases. In this study, cases and controls were prospectively accumulated, that is, for each new case diagnosed and included in the study, two new controls were chosen and also included in the study (2:1). Thus avoiding the inclusion of prevalent cases, which would be a potential source of bias.~~

~~The letters of informed consent, questionnaires, and all other correspondence with study participants were approved by the INCA Research Ethics Committee under registration number 135/11.~~

Fig 1. Research participants flow diagram

Data collection

The individual interviews of the research participants were carried out by qualified and trained professionals from the Technical Area Environment, Work and Cancer of the

National Cancer Institute (INCA; Ministry of Health; Brazil). On the day of the medical appointment or some routine procedure at the hospital, the patients were invited to participate in the research by professionals from the INCA research group. Those patients who agreed to participate in the research signed the Free and Informed Consent Form (TCLE). Data collection was carried out through a standardized and pre-tested questionnaire with questions aimed at obtaining sociodemographic, clinical and occupational information.

In the same period, companions and visitors of patients diagnosed with non-hematological neoplasms undergoing medical care at the outpatient clinics or wards of the Cancer Hospital I (HCI) of the National Cancer Institute (INCA) were invited to participate in the research to compose the control group. In addition, there was a need to recruit donors of biological materials in the Hemotherapy Service (HC1/INCA), since most of the companions and visitors of patients diagnosed with non-hematological neoplasms undergoing medical care at the hospital's outpatient clinics or wards INCA HCI were female. Therefore, on recruitment days, donors of biological materials at the Hemotherapy Service (HC1/INCA) were also invited to participate in the research.

The main outcome of the study was total NHL, and secondary outcomes, such as subtypes B-cell lymphoma, DLBCL, T-cell lymphoma were also included in the study. Occupational exposures were considered as explanatory variables. In addition to these exposures, the literature mentions age, gender, race/ethnicity, family history, autoimmune diseases and some infections as risk factors for NHL.

Thus, in orderTo to characterize the study population and control possible confounding factors, the following information was collected on the*abstracted for analysis* subject’s: demographic and clinic variables, including “year of interview” (2013, 2014, 2015

or 2016), "sex" (male, female), "age" (20-39; 40-59; >=60), "race" (white, no white), "region within the state of Rio de Janeiro" (metropolitan, others), "*years at the current residence*time of residence" (≤ 20, >20), "education" (no degree of education or primary school, high school, university degree), "employment actual": yes (private institution, retired, self-employed, public institution, unnot employed, dependent, pensioner, sick leave), "marital status (no companion, with companion, widowed), "family income - minimum salaries" (mean/SD).

Among the lifestyle variables, we considered the following:In relation to habits and past illness were included: "smoking habit" (never smoked, current smoker, past smoker: ≤ 10 years, past smoker: 10+ years), "smoke load: pack years" (never, daily: <10years, 10+years, others), "cellular phone use" (never, daily: ≤ 10 years, daily: 10+years, others)., "past illnesses" (dengue, pneumonia, HHV8 infection: herpesvirus-8, asma, *H. pylori* infection, diabetes, tuberculosis, HCV infection: hepatitis C virus, malaria, HTLV infection, EBV infection: Epstein-Barr virus). For this last variable ("past illnesses"), more than one response was considered.

The occupational history was limited to their main occupations in the last 20 working years and all occupations with working time greater than 1 year were considered. It was decided to establish a cut-off point lasting one year or more greater than 1 year for the exposure time. due to the difficulty of making a causal link between the short exposure time and the development of non-Hodgkin's lymphoma. Inform Information on occupational exposure to chemical substances, physical and biological agents possibly associated with the risk of developing NHL such as solvents, paints, metals, pesticides, cleaning products, biological material, animal and viscera material, industrial radiation and solar radiation (never, <= 10 years, 10+ years) were self-reportedcollected. The participant responded about

which agents he was exposed to and for how long, in the last 20 years, for each reported occupation. Although the questionnaire contains this information, it was decided to present the results keeping the exposures grouped (any solvents, hydrocarbons solvents, pesticides, metals, visceral and animal material, and solar radiation), without specifying which solvents, metals or pesticides were reported. The description of the exposures reported by the study participants, which agents were contained in each group, can be seen at the bottom of Table 3

Statistical analysis

A descriptive analysis was performed to verify the profile of the studied population. The variables were categorized according to the cutoff points pre-established in the literature. To assess differences in the distributions of categorical variables between the comparison groups (cases and controls), Pearson's chi-square tests or Fisher's exact test (for cases where the variable was dichotomous and there were less than five individuals in a cell) were performed. Missing values were excluded from the analysis and the information was reported in tables 1 and 2. For continuous variables, normality was tested (Kolmogorov test). When the variable had a normal distribution, Student's t-test was used to compare the means; otherwise, the Mann-Whitney non-parametric test was used.

By unconditional logistic regression, multivariate models were estimated to analyse exposure to chemical, physical, and biological agents as well as the risk of NHL according to the cases "Total NHL", "B-cell lymphoma", "DLBCL", "Follicular lymphoma", and "T-cell lymphoma". The covariates used in the study were:" with the following: "Risk of NHL (any subtype), B-cell lymphoma, DLBCL, Follicular lymphoma, and T-cell lymphoma associated with exposure to chemical, physical, and biological agents was analysed by unconditional regression. The

multivariate regression model included the following covariates:By unconditional logistic regression, multivariate models were estimated to analyze exposure to chemical, physical, and biological agents as well as the risk of NHL according to the cases “Total NHL”, “B-cell lymphoma”, “DLBCL”, “Follicular lymphoma”, and “T-cell lymphoma”. The covariates used in the study were: gender, age, skin color, family income, place of residence, level of education, smoking, alcoholism, past illnesses, and also family history of hematological cancer, including the history of NHL (described in the “Data collection” section and in the footer of table 3). To select the covariates to be included in the final regression model, we followed a stepwise forward procedure testing first the covariates with a p<0.25 association in the univariate analyses of the covariates with NHL (any subtype)[15].To select the covariates that composed the adjustment to the multivariate models, bivariate analyzes of the covariates with the study outcome (NHL) were initially performed, considering a p-value < 0.250 [15]. For multivariate analysis, logistic regression models were used using the manual stepwise progressive (forward) methodology, where the occupational exposure of interest was tested and the adjustment of each model was evaluated by inserting each of the previously selected covariates. The Odds Ratio and its 95% confidence interval were calculated as the measure of association; the null hypothesis was rejected if the 95% confidence interval did not include unity.”

At the end, a 5% significance level was considered for the control of possible confounding assessed using the odds ratio (OR) and its respective 95% confidence interval (95% CI). The software used was SPSS 25.0.

The letters of informed consent, questionnaires, and all other correspondence with study participants were approved by the INCA Research Ethics Committee under registration number 135/11.

RESULTS

A total of 214 NHL cases (112 men and 102 women) and 452 controls (207 men and 245 women) were included in the study. Regarding the cases of NHL, the predominant histological type was B-cell lymphoma (185 cases; 86.0%) and T-cell lymphoma were 30 cases (14.0%).

There was no statistical difference in age ($p = 0.063$) between the two groups. Most individuals were older than 40 years, with mean age of cases (56 years; standard deviation, SD = 12.7 years) and controls (52.0 years; SD = 12.2). As can be seen, the broad age categorization, the sex, race and marital status did not affect the comparability of cases and controls. An overview of the demographic characteristics of the NHL case–control sets is in Table 1.

Table 1: Sociodemographic characteristics of the participants in a case-control study, RJ, Brazil, 2013–2016.

**Characteristic**	**Controls (n = 452)**		**Cases (n = 214)**		**p-value**
	n	%	n	%	
**Year of the interview**					0.058
2013	116	25.7	58	27.1	
2014	90	19.9	56	26.2	
2015	163	36.1	76	35.5	
2016	83	18.34	24	11.2	
**Sex**					0.135
Male	207	45.8	112	52.3	
Female	245	54.2	102	47.7	
**Age**					0.063
20 – 39	85	18.8	33	15.3	
40 – 59	235	52.0	99	46.1	
> 60	132	29.2	82	38.6	
**Region within the state of Rio de Janeiro**					**< 0.001**
Metropolitan	426	94.2	171	79.9	
Others	26	5.8	43	20.1	
**Time of residence (Rio de Janeiro State)**					0.671
< 20	267	59.5	103	57.8	
> 20	182	40.5	90	42.2	
No information	3		1		
**Education**					**< 0.001**
No degree of education or primary school	124	27.5	101	47.1	
High school	194	43.0	81	37.9	
University degree	133	29.5	32	15.0	
No information	1				
**Employment (actual)**					
Private institution (yes)	111	24.5	25	11.7	**< 0.001**
Retired (yes)	97	21.5	61	28.6	**0.042**
Self-employed (yes)	96	21.2	21	9.9	**0.001**
Public Institution (yes)	71	15.7	14	6.6	**0.001**
Not employed (yes)	63	13.9	25	11.6	0,422
Dependent (yes)	16	3.5	11	5.1	0,322
Pensioner (yes)	13	2.8	14	6.5	0.023
Sick leave (yes)	7	1.5	59	27.4	**< 0.001**
**Marital status**					0.075
No companion	125	27.7	73	34.2	
With companion	309	68.5	129	60.2	
Widowed	17	3.8	12	5.6	
No information	1		0		
**Family income (Minimum salaries)**					**< 0.001**
Mean (Std. Dev)		4.1 (5.9)		2.7 (3.6)	

Values correspond to the absolute frequencies of the variables.
The p-value is related to the comparison of variable proportions between NHL cases and controls, using the chi-square or Fisher test (for strata with absolute number < 5).
The p-value corresponds to the comparison of variable means between NHL cases and controls using the Mann-Whitney test
In bold, values with a statistically significant result (< 0.05). 1 minimum salarie in Brazil in 2016 was equal to 880 reais.(220 USD)
* Missing values cases = 1

Table 2 elucidates that there was no statistical difference in smoking status ($p = 0.074$) between the two groups, although the smoking burden was higher in the cases than in the controls (> 20 pack years: 26.3% vs 12.3%, $p < 0.001$). Current alcohol consumption (1 to 5 drinks per week) was lower in the cases than in the controls (26.8% vs 40.0%, $p = 0.003$). Use of cell phones and wireless phones was more frequent and more prolonged among the controls than the cases ($p < 0.001$). Regarding previous diseases, the cases of NHL presented a higher percentage of diabetes (15.1% vs 5.7%, $p < 0.001$) and diagnosis of Human T-lymphotropic virus infection (2.9% vs 0.2%, $p = 0.005$) than the controls.

Table 2: Habits and past illnesses of the participants in a case-control study, RJ, Brazil, 2013-2016.

**Characteristic**	**Controls (n = 452)**		**Cases (n = 214)**		**p-value**
	n	%	n	%	
**Smoking status**					0.074
Never smoked	283	65.5	117	57.1	
Current smoker	46	10.6	20	9.8	
Past smoker (< 10 years)	39	9.0	30	14.6	
Past smoker (10+ years)	64	14.9	38	18.5	
No information	20		9		
**Smoke load (Pack years)**					**< 0.001**
Never	283	65.7	117	57.1	
< 20	93	21.5	34	16.6	
> 20	53	12.3	54	26.3	
No information	23		9		
**Alcohol consumption (current)**					**0.003**
Non-drinker	204	46.9	120	60.6	
1 to 5 drinks per week	174	40.0	53	26.8	
> 5 drinks per week	57	13.1	25	12.6	
No information	17		16		
**Cellular use**					**< 0.001**
Never	22	4.9	27	13.5	
Daily (< 10 years)	144	32.1	50	24.0	
Daily (10^{+}years)	224	49.9	86	41.3	
Others	59	13.3	44	21.2	
No information	3		7		
**Wireless Phone use**					**< 0.001**
Never	152	34.0	106	52.2	
Daily (< 10 years)	61	13.6	25	12.2	
Daily (10^{+}years)	94	21.1	30	14.6	
Others	140	31.3	43	21.0	
No information	5		10		
**Past Illnesses**					
Dengue	145	32.2	68	33.0	0.841
Pneumonia	57	12.8	34	16.7	0.187
HHV8 infection (Herpesvirus-8)	53	11.8	34	16.7	0.097
Asma	45	10.1	14	6.8	0.183
H. pylori infection	38	8.4	15	7.3	0.610
Diabetes	26	5.7	31	15.2	**< 0.001**
Tuberculosis	11	2.7	2	1.0	0.246
HVC infection (Hepatitis C virus)	9	2.0	5	2.5	0.773
Malaria	1	0.2	3	1.4	0.094
HTLV infection	1	0.2	6	2.9	**0.005**
EBV infection (Epstein-Barr virus)	1	0.2	2	1.0	0.233

Values correspond to the absolute frequencies of the variables.
The p-value is related to the comparison of variable proportions between NHL cases and controls, using the chi-square or Fisher test (for strata with absolute number < 5).
The p-value corresponds to the comparison of variable means between NHL cases and controls using the Mann-Whitney test
In bold, values with a statistically significant result (< 0.05).
*Missing values: controls = 2; cases = 8

Table 3 provides the results from multivariate unconditional logistic regression. of occupational exposure to chemical, biological, and physical agents as well as NHL development and histological subtypes. The reference group consisted of individuals who were not occupationally exposed to these substances/agents.

Exposure to any solvents and pesticides was significantly associated with an increased risk of B- and T-cell lymphoma. We observed a positive dose-response relationship between years of exposure to these substances and increased risk for total NHL.

For occupational exposure to any solvents, hydrocarbons solvents and pesticides, the chance of developing total NHL was 2.12, 1.91 and 2.70 times greater, respectively, in the cases exposed for more than 10 years, compared to the controls (OR = 2.12 95%CI 1.30–3.48; OR = 1.91 95%CI 1.14–3.21; OR = 2.70 95%CI 1.01–7.21).

B-cell lymphoma was significantly associated with occupational exposure to any of those solvents (> 10 years: OR = 1.92; 95%CI 1.15–3.21) and hydrocarbon solvents (> 10 years: OR = 1.73; 95%CI 1.01–2.96).

Risk estimates were stronger for T-cell lymphomas, but wide confidence intervals suggest caution in interpreting them. The chance of developing T-cell lymphomas was 3.58 times greater in the cases exposed for more than 10 years compared to the controls (any solvents > 10 years: OR = 3.58 95%CI 1.22–10.48). Inserir p de tendência.

Occupational exposure to pesticides was significantly associated with an increased risk of total NHL, follicular lymphoma and T-cell lymphoma. We observed a dose-response association between years of exposure to these substances and increased risk of total NHL (pesticides>10 years: OR = 2.70 95%CI 1.01–7.21). Strong associations were found between the development of T-cell lymphoma and exposure to pesticides in the workplace. The

chance of developing T-cell lymphomas was 10.65 times greater in the cases exposed to pesticides for more than 10 years, compared to the controls (pesticides>10 years: OR = 10.65; 95%CI 2.35–48.24), The strongest association was observed with risk of T-cell lymphoma, although the small number of subjects suggests caution in the interpretation.but with a large confidence interval.

Work with animal and viscera materials increased 4-fold the risk of total NHL, B-cell lymphoma, and Diffuse large B-cell lymphoma (DLBCL) subtypes (> 10 years: OR = 4.19 95%CI 1.22–13.85; OR = 3.29 95%CI 1.06–10.21; OR 4.73 95%CI 1.37–16.73, respectively). The small number of subjects also suggests caution in the interpretation.

We observed that the chance of developing B-cell lymphoma was 2.47 times higher in the cases exposed to solar radiation for more than 10 years, compared to the controls (OR = 2.47 95%CI 1.21–5.04). Overall, the chance that those occupationally exposed for at least 10 years to solar radiation had NHL was more than double compared to the controls. Follicular lymphoma was significantly associated with occupational exposure to solar radiation (> 10 years: OR = 4.49 95%CI 1.40–14.36).

No statistically significant associations were found between some exposures at work (dyes, sanitizing products, biological material, and industrial radiation) and NHL.

Table 3: Occupational risk factors and development of Total NHL, B-cell lymphoma, DLBCL, follicular lymphoma and T-cell lymphoma in the participants in a hospital-based case-control study, RJ, Brazil, 2013-2016.

Risk Factors	Total NHL		B-cell lymphoma		DLBCL		Follicular lymphoma		T-cell lymphoma	
	Controls/ Cases	OR adjusted 95% CI	Controls/ Cases	OR adjusted 95% CI	Controls/ Cases	OR adjusted 95% CI	Controls/ Cases	OR adjusted 95% CI	Controls/ Cases	OR adjusted 95% CI
Chemical										
**Any solvents**										
Never	275/93	1.0	275/83	1.0	275/38	1.0	275/15	1.0	275/10	1.0
< 10 years	79/51	**1.70 1.04 to 2.79**[a]	79/43	1.51 0.9 to 2.55[e]	79/18	**1.94 1.01 to 3.73**[g]	79/12	**3.43 1.40 to 8.39**[j]	79/8	**3.42 1.17 to 9.99**[j]
10+ years	77/54	**2.12 1.30 to 3.48**[a]	77/47	**1.92 1.15 to 3.21**[e]	77/19	1.64 0.88 to 3.09[g]	77/11	**3.03 1.25 to 7.31**[j]	77/7	**3.58 1.22 to 10.48**[j]
**p trend**		< 0.001		< 0.001		0.031		0.028		0.023
**Dyes**	-	-					-	-		
Never	402/179	1.0	402/157	-	402/69	-	402/34	-	402/22	-
< 10 years	14/9	1.26 0.45 to 3.48[a]	14/7	-	14/3	-	14/1	-	14/2	-
10+ years	15/10	1.60 0.61 to 4.21[a]	15/9	-	15/3	-	15/3	-	15/1	-
**p trend**		0.208								
**Hydrocarbon solvents**		-								
Never	297/107	1.0	297/93	1.0	297/43	1.0	297/18	1.0	297/14	1.0
< 10 years	66/43	1.65 0.99 to 2.76[a]	66/38	1.57 0.91 to 2.71[e]	66/16	1.87 0.96 to 3.68[g]	66/10	**2.84 1.15 to 7.00**[k]	66/5	1.66 0.54 to 5.08[j]
10+ years	68/49	**1.91 1.14 to 3.21**[a]	68/42	**1.73 1.01 to 2.96**[e]	68/16	1.40 0.73 to 2.70[g]	68/10	**2.70 1.12 to 6.48**[k]	68/6	2.47 0.86 to 7.08[j]
**p trend**		< 0.001		< 0.001		0.056		0.053		0.169
**Metals**	-									
Never	403/179	1.0	403/158	-	403/69	-	403/35	-	403/21	1.0
< 10 years	13/10	1.92 0.65 to 5.72[b]	13/7	-	13/2	-	13/2	-	13/3	3.36 0.77 to 14.71[n]
10+ years	15/9	1.22 0.42 to 3.52[b]	15/8	-	15/4	-	15/1	-	15/1	1.47 0.17 to 12.54[n]
**p trend**		0.176								0.082

Risk Factors	Total NHL Controls/ Cases	Total NHL OR adjusted 95% CI	B-cell lymphoma Controls/ Cases	B-cell lymphoma OR adjusted 95% CI	DLBCL Controls/ Cases	DLBCL OR adjusted 95% CI	Follicular lymphoma Controls/ Cases	Follicular lymphoma OR adjusted 95% CI	T-cell lymphoma Controls/ Cases	T-cell lymphoma OR adjusted 95% CI
**Pesticides**										
Never	390/156	1.0	390/137	1.0	390/59	1.0	390/28	1.0	390/19	1.0
< 10 years	20/19	**2.41 1.13 to 5.13**[a]	20/16	2.15 1.0.96 to 4.84[f]	20/5	1.76 0.60 to 5.14[g]	20/8	**4.38 1.61 to 11.93**[j]	20/3	**4.04 1.04 to 15.69**[i]
10^{+}years	10/13	**2.70 1.01 to 7.21**[a]	10/10	2.38 0.84 to 6.77[f]	10/5	2.82 0.86 to 9.19[g]	10/2	3.05 0.56 to 16.78[j]	10/3	**10.65 2.35 to 48.24**[i]
**p trend**		**< 0.001**		0.001		0.035		< 0.001		**0.002**
**Sanitizing products**										
Never	242/95	1.0	242/82	1.0	242/37	1.0	242/16	1.0	242/13	-
< 10 years	66/40	1.15 0.65 to 2.02[c]	66/34	1.17 0.65 to 2.14[f]	66/15	1.73 0.86 to 3.52[i]	66/9	2.00 0.80 to 5.02[l]	66/6	-
10^{+}years	62/49	1.41 0.80 to 2.49[c]	62/43	1.47 0.81 to 2.66[f]	62/17	1.76 0.88 to 3.53[i]	62/11	2.21 0.90 to 5.41[l]	62/6	-
**p trend**		0.001		0.001		0.053		0.052		
Biological **Biological material**	-						-			
Never	403/187	1.0	403/164	-	403/71	-	403/37	-	403/23	-
< 10 years	19/7	0.35 0.10 to 1.25[b]	19/6	-	19/3	-	19/1	-	19/1	-
10^{+}years	9/4	0.78 0.20 to 2.96[b]	9/3	-	9/1	-	9/0	-	9/1	-
**p trend**		0.656								
**Animal and visceral material**										
Never	417/175	1.0	417/152	1.0	417/66	1.0	417/33	**1.0**	417/23	1.0
< 10 years	6/11	**4.16 1.33 to 13.06**[b]	6/10	**4.35 1.38 to 13.67**[e]	6/3	3.12 0.69 to 14.19[h]	6/4	**13.07 2.79 to 61.12**[j]	6/1	3.64 0.39 to 33.99[j]
10^{+}years	8/12	**4.19 1.22 to 13.85**[b]	8/10	**3.29 1.06 to 10.21**[e]	8/6	**4.73 1.37 to 16.73**[h]	8/1	4.27 0.47 to 38.48[j]	8/1	2.92 0.33 to 25.77[j]
**p trend**		**< 0.001**		**< 0.001**		**0.001**		0.156		0.212

365

Risk Factors	Total NHL		B-cell lymphoma		DLBCL		Follicular lymphoma		T-cell lymphoma	
	Controls/ Cases	OR adjusted 95% CI	Controls/ Cases	OR adjusted 95% CI	Controls/ Cases	OR adjusted 95% CI	Controls/ Cases	OR adjusted 95% CI	Controls/ Cases	OR adjusted 95% CI
Physical										
**Industrial radiation**										
Never	411/191	1.0	411/167	-	411/72	-	411/37	-	411/24	-
< 10 years	11/4	1.04 0.29 to 3.70[d]	11/4	-	11/2	-	11/1	-	11/0	-
10^{+}years	9/3	0.28 0.05 to 1.52[d]	9/2	-	9/1	-	9/0	-	9/1	-
**p trend**		0.525								
**Solar radiation (occupational)**										
Never	354/136	1.0	353/116	1.0	353/51	1.0	353/24	1.0	353/20	-
< 10 years	56/36	1.51 0.86 to 2.63[a]	56/32	**1.80 1.02 to 3.22[e]**	56/15	**2.13 1.02 to 4.47[h]**	56/9	**4.14 1.64 to 10.45[m]**	56/4	-
10^{+}years	21/26	1.91 0.93 to 3.90[a]	21/25	**2.47 1.21 to 5.04[e]**	21/9	2.23 0.90 to 5.58[h]	21/5	**4.49 1.40 to 14.36[m]**	21/1	-
**p trend**		< 0.001		**< 0.001**		0.003		**0.085**		

Total NHL: Total Non-Hodgkin's Lymphoma; DLBCL: Diffuse large B-cell lymphoma; OR: odds ratio; CI: confidence interval.
The data shows missing data.
Any solvents: turpentine, tar, asphalt, benzene, benzine, cleaners or degreaser, synthetic dye hardener, formaldehyde, gasoline, used motor oil, diesel oil, lubricating oil, crude oil, tar, kerosene, reducing agent (not specific), remover (not specs), thinner.
Hydrocarbon solvents: tar, benzene, gasoline, kerosene, thinner (not spec), remover (not spec), thinner.
Metals: chrome, iron, and lead
Pesticides: Pesticides, insecticide, rodenticide, ant killer
[a] OR adjusted for sex, age, place of residence, household income, birthplace, retired, diabetes, cell phone use, wireless phone use, alcohol consumption, HHV8 infection.
[b] OR adjusted for sex, age, place of residence, household income, birthplace, retired, diabetes, cell phone use, wireless phone use, alcohol consumption, HHV8 infection, smoking.
[c] OR adjusted for sex, age, place of residence, household income, birthplace, retired, diabetes, cell phone use, wireless phone use, alcohol consumption.
[d] OR adjusted for sex, age, place of residence, household income, birthplace, retired, diabetes, cell phone use, wireless phone use, HHV8 infection.
[e] OR adjusted for sex, age, place of residence, household income, birthplace, retired, diabetes, cell phone use.

[f] OR adjusted for sex, age, place of residence, household income, birthplace, retired, diabetes, cell phone use, wireless phone use.
[g] OR adjusted for sex, age, place of residence, place of birth.
[h] OR adjusted for sex, age, place of residence, household income, place of birth.
[i] OR adjusted for sex, age, place of birth.
[j] OR adjusted for sex, age, place of residence, diabetes, year of the interview, HHV8 infection.
[k] OR adjusted for sex, age, place of residence, diabetes, year of the interview.
[l] OR adjusted for sex, age, place of residence, year of the interview.
[m] OR adjusted for sex, age, place of residence, diabetes.
[n] OR adjusted for sex, age, retired, cell phone use, alcohol consumption, HHV8 infection.
The categories with models not estimated in the table refer to bivariate analyzes with p>0.25.

Ocupações que mais representam a exposição à exposição a materiais biológicos (Enfermeiro, técnico de enfermagem).

Ocupações que mais representam a exposição à radiação solar (comerciante, doméstica, do lar, eletricista, motorista, pedreiro, pintor, policial militar, segurança, vendedor).

DISCUSSION

In this study, we found an association between NHL and the following occupational exposures: total solvents, hydrocarbons, pesticides, sunlight and sunlight, when compared to those not occupationally exposed to these factors. These associations were maintained even after adjusting for variables that could confound them (described at the bottom of Table 3).

A significant upward trend with years of exposure was detected for solvents and hydrocarbon solvents (NHL any subtype), B-cell lymphoma, and T-cell lymphoma), pesticides (NHL (any subtype), B-cell lymphoma, DLBCL, and T-cell lymphoma, sunlight (NHL (any subtype) and DLBCL), and sunlight (B-cell lymphoma, DLBCL and follicular lymphoma. Não foram encontradas associações entre LNH em expostos a tintas, a metais, a produtos de limpeza a agentes infecciosos, a radiação industrial (any sub-types).

Most of the studied NHL cases were diagnosed as B-cell lymphoma, with a higher incidence of diffuse large B-cell lymphoma (DLBCL) (82; 38.1%) and follicular lymphoma (40; 18.6%). This corresponds to the incidences found in the literature for the different histological subtypes of NHL, which are the high-grade and aggressive subtypes [1,16].

Men exhibited a higher frequency of NHL cases. This demographic profile agrees with estimates of the Brazilian National Cancer Institute [6] and with the profile of cases described in the literature. The mean age of cases was 56 years old (SD = 12.7 years), with the largest portion of individuals over 45, in accordance with the InterLymph consortium [17–20]. It is noteworthy that the broad age categorization did not affect the comparability of cases and controls by gender and case-control status.

Both cases and controls reside in the metropolitan mesoregion of the Rio de Janeiro state. This data has also a limitation. As INCA is a reference hospital in cancer and one of the

only ones to offer comprehensive and free treatment for cancer (public health), patients from all over the state of Rio de Janeiro want to be treated at INCA. Therefore some individuals, from others regions, outside the metropolitan region, came to INCA. In addition, it is important to emphasize that most individuals stop working, retire, or take a leave of absence after discovering the disease, which lowers the family income. This could be one of the explanations for the lower income of the cases compared to the controls [21].

For follicular and marginal zone lymphoma, smoking is a risk factor [20,22]. The immunological effects observed in smokers, such as immunosuppression and increased leukocyte production, may be associated with the development of lymphomas [23]. In addition, cigarette smoke contains substances known to be carcinogens, including benzene and formaldehyde [24]. In a cohort study conducted in the United States with 1,926 cases of NHL, smoking (former smokers) was associated with the occurrence of NHL (histological type of T-cells) [25]. The present study found no statistical differences in smoking between the two groups, although the smoke load (pack years) was higher among the cases than the controls. It is noteworthy that many patients stop smoking after being diagnosed with cancer. Therefore, the answers given by patients when asked about being current smokers may reflect the condition of no longer smoking after the diagnosis of NHL.

No increase in alcoholic beverages consumption was observed among the cases after analyzing the information on the number of doses of alcoholic beverages consumed per week; therefore, the frequency found in the present study (61%) was lower than in other studies [26].

Diabetes had a statistically significant difference between the comparison groups (cases: 15.2%; controls: 5.7%, $p < 0.001$). This excess risk is different from that found in

observational studies, which investigated the association of diabetes and NHL [27]. In case-control studies, an association of diabetes with NHL was observed, without statistical significance (OR: 1.12; 95%CI 0.95–1.31), in other types of studies a risk was found for women (RR: 1.38, 95%CI 1.06–1.80 [28]. Types 1 and 2 diabetes can cause immunosuppression because of immunological dysfunction due to decreased neutrophil activity and alterations in cellular and humoral immunity. In addition, most patients with type 2 diabetes are obese, and it is well established in the literature that excess body fat increases the risk of various malignancies [27]. A limitation of the study related to this variable is that clinical anamnesis and laboratory tests were not performed on controls to confirm the presence or absence of diabetes. Since the result comes from the individual's self-report when asked whether or not they had diabetes.

Like the acquired human immunodeficiency virus (HIV), the Human T-cell lymphotropic virus (HTLV) is a lymphotropic retrovirus, with a predilection for CD4+ T lymphocytes. Transmission occurs through biological fluids such as blood, vaginal fluid, breast milk, and semen . An estimated 5 to 200 million people have been infected with HTLV worldwide [29], and the Brazilian Ministry of Health estimates that between 700,000 and 2 million are infected there, making Brazil the country with the highest number of cases [30] . In our study, 6 (2.9%) of the cases reported being HTLV virus carriers and only 1 (0.2%) of the controls was infected. Statistical analysis demonstrated a predisposition to develop NHL in individuals infected with HTLV (p-value = 0.005). Most HTLV carriers are asymptomatic and only about 5% of those infected will develop some type of complication. Several diseases associated with HTLV, including neurological, psychiatric, ophthalmological, dermatological, and lymph proliferative manifestations [30] . HTLV-1 is

the first oncogenic retrovirus in humans [31] and is considered the etiologic agent of adult T-cell leukemia/lymphoma (ATLL), which can have a broad clinical presentation, ranging from indolent to aggressive. Although the genetic material of HTLV-2 is 70% identical to HTLV-1, it has not yet been related to lymph proliferative diseases. A limitation of the study related to this variable is that laboratory tests were not performed on controls to confirm the presence or absence of HTLV.

Hepatitis C virus (HCV) is a hepatotropic RNA virus transmitted through blood, either by transfusions, sharing needles or biological accidents. Clinical manifestations include acute hepatitis, chronic hepatitis, liver cirrhosis, and autoimmune diseases. A recent meta-analysis performed in USA has shown that HCV is a significant risk factor for NHL, with an odds ratio of 2.6 compared to the non-HCV population [32]. Despite the results found, our study did not find a positive association between contamination by HCV and the risk of developing NHL, with only 5 (2.5%) of the patients being infected, in contrast to 9 (2%) of the controls.

Among the categories of solvents present in the occupational exposure questionnaire questions, benzene was one of the chemical agents most reported by study participants. In Brazil, according to Corrêa et al (2016) [27], 7,376,761 (8.5%) workers belong to occupational groups potentially exposed to benzene, while 770,212 workers are considered definitively exposed to benzene. Occupational exposure to benzene and other aromatic hydrocarbons and the possibility of developing NHL have been investigated in other works. An Italian study by Miligi et al. [28], which evaluated 1428 cases of NHL and 1530 controls in eight areas of Italy, through individual interviews and questionnaires, found a positive association for exposure to benzene (OR: 1.60; 95%CI 1.0–2.4) and aromatic hydrocarbons

(OR: 1.20; 95%CI 0.9–1.7) between the cases. A meta-analysis found in 22 studies of benzene exposure a 22% increased risk of NHL (OR: 1.22; 95%CI 1.02–1.47). When the estimate was calculated excluding the based studies only in the self-reported occupational history, with 6 studies remaining, the risk was approximately 2 times higher for NHL (OR: 2.12, 95%CI 1.11–4.02) [29]. Other aromatic hydrocarbon solvents (styrene, BTX, toluene, xylene, and others) have been associated with NHL [30–32]. The main mechanism of carcinogenicity is reported to be the genotoxic effect. It is worth mentioning that workers are rarely exposed to a single agent, since workplaces containing benzene, also expose them to a complex mixture of other aromatic hydrocarbons, such as toluene, xylene, ethylbenzene; as well as other hydrocarbons derived from petroleum during the complex processes arising from petroleum refining, the production of coke, oils and naphtha [33].

The wide presence of heavy metals in occupational, industrial, and agricultural environments leads to increased occupational exposure and concern about their health effects [34]. In the present study, no association was found between metal exposure and overall NHL or its subtypes, although a significant odds ratio was observed for overall NHL (OR= 1.92 CI: 0.65-5.72 for those exposed <= 10 years) and for follicular lymphoma (OR= 1.22 CI: 0.42-3.52 for those exposed >= 10 years), albeit without statistical significance. The same was observed for follicular lymphoma (OR= 3.36 CI: 0.77-14.71 for those exposed <=10 years and OR= 1.47 CI: 0.17-12.54 for those exposed >= 10 years), also without statistical significance. This result is consistent with a study conducted in Australia that similarly assessed the risk of developing NHL in individuals with occupational exposure to metal dust, which did not show a positive association[35]. but Kelly et al [36] conducted a study in Italy and Sweden that observed a positive association in women between heavy metal levels in the

blood and the risk of developing NHL. Further studies are needed to clarify this association found in this study.

The wide presence of heavy metals in occupational, industrial, and agricultural environments leads to increased occupational exposure and concern about their health effects [40]. In the present study, exposure to heavy metals increased five-fold the risk of developing T-cell lymphoma. This result is in line with a multicenter study by Kelly et al [41] conducted in Italy and Sweden that observed a positive association in women between heavy metal levels in the blood and the risk of developing NHL. Another study in Australia that assessed the risk of developing NHL in individuals with occupational exposure to metal dust did not demonstrate a positive association [42]. Several metals, such as lead and cadmium, are classified as carcinogens, and the hematopoietic system is one of the targets, as alterations in immune function were found [40]. Metal-induced carcinogenesis depends on the level and duration of exposure, in addition to the individual variation in the response of the organism [41]. Data in the literature are still inconsistent and scarce; nevertheless, the results of this study found a positive association between exposure to metals and the development of certain types of NHL. Subsequent studies are needed to clarify this association.

Case-control, cohort, and meta-analysis studies have explored the association between exposure to active ingredients of specific pesticides and the NHL development [37]. The IARC classified some pesticides as exhibiting carcinogenic potential after observing a positive association between pesticides exposure and NHL development. DDT, diazinon, glyphosate, and malathion have been classified as probably carcinogenic to humans (group 2A) [24]. Furthermore, 2,4-D, chlordane, heptachlor, hexachlorobenzene, lindane, mirex, and

pentachlorophenol were classified as possibly carcinogens to humans (group 2B) [24]. Glyphosate and 2,4-D are among the most used pesticides in Brazilian crops. Leon et al. [38] identified an association between NHL and occupational exposure to pesticides in cohorts from France (Agriculture and Cancer – AGRICAN); Norway (Cancer in the Norwegian Agricultural Population – CNAP); and the USA (Agriculture Health Study – AHS). For most of the chemical groups in the pesticides evaluated, no associations were observed in 33 active ingredients. However, increased total NHL risk was observed in farmers who used terbufos; CCL/SLL among those who used deltamethrin; and DLBCL among those who used glyphosate. It should be clarified that terbufos is an extremely toxic organophosphate insecticide; deltramethrin is an insecticide of the pyrethroid group, and glyphosate is an herbicide. All three are widely used in Brazil, including in unauthorized cultures.

The results of this study also showed the association of exposure to animal materials and viscera with an increased chance of developing NHL. These results corroborate the results found in the case-control study conducted in eight Canadian provinces by Fritschi et al. [39], who reported an increased risk between exposure to beef cattle and NHL; as well as by Tranah et al [40] who observed an increased risk of NHL in individuals who lived or worked with cattle (1.6, CI=1.0–2.5) and pigs (OR = 1.5; CI=1.1–2.1). With exposure to pigs for ≥ 5 years, the increased risk also occurred for NHL (OR = 1.8; CI=1.2–2.6) and for diffuse large-cell and immunoblastic large-cell lymphoma (DLCL) (OR = 2.0, CI=1.2-3.4). According to the authors, contact with infectious agents as a result of work with livestock or contact and handling of pork and beef increases the risk of NHL, as they can carry lymphomagenic infectious agents. Freeman et al. [41] analyzing data from the Agricultural Health Study, observed increased risk of NHL (RR = 1.6, 95%CI1.0–2.4) among those who

raised poultry and those who were veterinarians on poultry farms (RR = 12.2; 95%CI:1.6–96.3) [40].

In New Zealand, Mannetje et al. [7] observed a 2-fold higher risk of NHL in workers who handled meat, especially those who worked at meat processing plants, when compared to controls. Although the studies described found some association between exposure and NHL, this association was not for all investigated animals or for all NHL subtypes. Freeman et al. [41] for example found an association for raising poultry but not for sheep; Fritschi et al. [39] found an association between NHL and DLCL for occupational exposure to beef cattle, but not to any other animal.

Our study indicated that occupational exposure to solar radiation for at least 10 years doubled the chance of total NHL, B-cell lymphomas and DLBCL, compared to the controls. Lu et al. [12] in a meta-analysis with 10 case-control studies and one cohort, in the period between 1997 and 2012, observed a positive association between occupational sun exposure and NHL (OR = 1.15; 95%CI 1.05–1.23), but not for the common subtypes of non-Hodgkin lymphoma (T-cell and B-cell lymphoma, and chronic lymphocytic leukemia). Similarly, Karipidis et al. [42] observed that the group with cumulative occupational exposure and minimum exposure time of 5 years before diagnosis had a 1.46-fold higher chance of having NHL (95%CI 1.06–2.02) than the unexposed. Although the association between occupational sun exposure and NHL was mentioned in the studies described here, other studies have not found this association [43,44]; therefore, more research is needed.

The authors considered it important to evaluate the association between subtypes of non-Hodgkin lymphomas and occupational exposures, since few articles carried out this investigation. However, for some types of occupational exposures, such as biological

material, paints, metals and industrial radiation, as the number of observations was very small, it was not possible to perform statistical analysis by subtypes of non-Hodgkin lymphoma. Therefore, only the results of the main outcome, total NHL, were evaluated. Furthermore, no signs of effect modification were observed when measures of association were compared for the strata of each variable for total NHL and for the different subtypes of non-Hodgkin's lymphoma.

Men exhibited a higher frequency of NHL cases. This demographic profile agrees with estimates of the Brazilian National Cancer Institute [6]MENDELEY CITATION PLACEHOLDER 45 and with the profile of cases described in the literature. The mean age of cases was 56 years old (SD = 12.7 years), with the largest portion of individuals over 45, in accordance with the InterLymph consortium [17–20]MENDELEY CITATION PLACEHOLDER 46. It is noteworthy that the broad age categorization did not affect the comparability of cases and controls by gender and case-control status.

Both cases and controls reside in the metropolitan mesoregion of the Rio de Janeiro state. This data has also a limitation. As INCA is a reference hospital in cancer and one of the only ones to offer comprehensive and free treatment for cancer (public health), patients from all over the state of Rio de Janeiro want to be treated at INCA. ThereforeTherefore, some individuals, from others regions, outside the metropolitan region, came to INCA. In addition, it is important to emphasize that most individuals stop working, retire, or take a leave of absence after discovering the disease, which lowers the family income. This could be one of the explanations for the lower income of the cases compared to the controls [21]MENDELEY CITATION PLACEHOLDER 47.

For follicular and marginal zone lymphoma, smoking is a risk factor [20,22]~~MENDELEY CITATION PLACEHOLDER 48~~. The immunological effects observed in smokers, such as immunosuppression and increased leukocyte production, may be associated with the development of lymphomas [23]~~MENDELEY CITATION PLACEHOLDER 49~~. In addition, cigarette smoke contains substances known to be carcinogens, including benzene and formaldehyde [24]. In a cohort study conducted in the United States with 1,926 cases of NHL, smoking (former smokers) was associated with the occurrence of NHL (histological type of T-cells) [25]~~MENDELEY CITATION PLACEHOLDER 50~~. The present study found no statistical differences in smoking between the two groups. , although the smoke load (pack years) was higher among the cases than the controls. It is noteworthy that many patients stop smoking after being diagnosed with cancer. Therefore, the answers given by patients when asked about being current smokers may reflect the condition of no longer smoking after the diagnosis of NHL.

No increase in alcoholic beverages consumption was observed among the cases after analyzing the information on the number of doses of alcoholic beverages consumed per week; therefore, the frequency found in the present study (61%) was lower than in other studies [26]~~MENDELEY CITATION PLACEHOLDER 51~~.

Limitações do estudo

Os autores consideram os resultados apresentados como de extrema relevância considerando a história de uso intenso de agentes cancerígenos classificados no Grupo 1 da IARC no país, alguns destes já banidos em outros países. Deve-se pontuar igualmente, a pouca disponibilização de estudos analíticos do tipo caso-controle no Brasil que incluem os fatores de risco ocupacionais para câncer. Outra questão relevante é a possibilidade desses resultados

fornecerem ferramentas para a vigilância em saúde do trabalhador de estados e municípios no que diz respeito a exposição de trabalhadores aos agentes trabalhados aqui. Porém, reconhecemos que esses resultados devem ser vistos com cautela, considerando alguns problemas inerentes ao próprio desenho do estudo. Os controles foram selecionados a partir dos acompanhantes e visitantes dos pacientes com diagnóstico de cânceres sólidos e também na sala de espera do Banco de sangue do Hospital, onde também recebe em sua maioria visitante e acompanhante dos pacientes. Esse procedimento foi adotado para minimizar o efeito de um possível viés de seleção de controles. Dessa forma, os controles selecionados no Banco de sangue não diferiram tanto dos controles captados nas enfermarias do Hospital. Deve-se ressaltar também que o INCA não é um hemocentro.Os autores tentaram minimizar o efeito de um possível viés de seleção de controles ao fazer a captação dos acompanhantes dos pacientes internados. Como esse grupo foi majoritariamente formado por mulheres, foi utilizada outra estratégia de captação, onde no momento da visita de parentes, amigos, vizinhos de pacientes internados, estes são convidados rotineiramente a serem doadores de sangue. Ao aceitarem, foram convidados a fazerem parte da pesquisa. Não foram captados doadores de sangue que não estivessem ali para a visita a um dos pacientes internados. Dessa forma, os doadores não diferiram tanto dos controles captados nas enfermarias. É óbvio que os que aceitaram doar sangue podem diferir um pouco dos que não doaram, em relação ao estado de saúde, considerando os doadores como indivíduos mais saudáveis, continuaram a ser parentes, vizinhos e/ou amigos. Deve-se ressaltar também que o INCA não é uma Instituição de captação de sangue convencional, como um hemocentro.

Outra questão que deve ser abordada, como possível limitação para os achados são as múltiplas comparações trabalhadas aqui. Este é um estudo exploratório onde buscou-se

avaliar várias exposições em um único estudo. Por esse motivo, não se pôde avaliar detalhes importantes como ocupação, atividades econômicas e exposições de forma mais desagregada, limitando a observância dos efeitos de forma mais específica, também não foi possível investigar múltiplas exposições simultâneas.

Há de se considerar também como limitação o viés de memória inerente ao desenho do estudo. Nesse caso, em específico, mais acentuado pela aplicação de um questionário para captação do recordatório do histórico ocupacional, necessário para análise da associação entre trabalho e LNH, nesse estudo. Solicitou-se a cada paciente que relatasse todas as ocupações exercidas nos últimos 20 anos. Possivelmente os casos se esforçaram para lembrar mais de possíveis exposições do que os controles.

Considera-se ainda que o estudo não contou com nenhuma avaliação ambiental ou de avaliação clínica de casos e controles. A única ferramenta utilizada foi um instrumento de coleta de dados, cujas informações foram obtidas por meio de entrevista. Ou seja, temos apenas autorrelatos de exposições referidos pelos entrevistados. Numa tentativa de observar-se a exposição cumulativa, considerou-se apenas anos de exposição a determinados agentes cancerígenos. Não houve como medir a intensidade de cada exposição.

Limitations of the study

The authors consider the presented results to be of utmost relevance considering the history of intensive use of carcinogenic agents classified in Group 1 by IARC in the country, some of which have already been banned in other countries. It should also be noted that there is a scarcity of analytical case-control studies in Brazil that include occupational risk factors for cancer. Another important issue is the possibility of these results providing tools for

occupational health surveillance in states and municipalities regarding workers' exposure to the agents studied here. However, we acknowledge that these results should be viewed with caution, considering some problems inherent to the study design itself.

The controls were selected from the companions and visitors of patients diagnosed with solid cancers, as well as from the waiting room of the hospital's Blood Bank, which also mostly receives visitors and companions of patients. This procedure was adopted to minimize the potential selection bias of controls. Thus, the controls selected at the Blood Bank did not differ significantly from the controls recruited in the hospital wards. It should also be emphasized that INCA is not a blood center.

The authors attempted to minimize the potential selection bias of controls by recruiting companions of hospitalized patients. As this group was predominantly composed of women, another recruitment strategy was used. During the visits of relatives, friends, and neighbors of hospitalized patients, they were routinely invited to become blood donors. Upon acceptance, they were invited to participate in the research. Blood donors who were not there for the visit of a hospitalized patient were not recruited. Thus, the blood donors did not differ significantly from the controls recruited in the hospital wards. It is evident that those who agreed to donate blood may differ slightly from those who did not donate, in terms of their health status, considering that the donors are likely to be healthier individuals who are still relatives, neighbors, and/or friends. It should also be noted that INCA is not a conventional blood collection institution like a blood center.

Another issue that should be addressed as a potential limitation for the findings is the multiple comparisons involved in this study. This is an exploratory study that sought to evaluate various exposures in a single study. For this reason, important details such as occupation, economic activities, and exposures could not be assessed in a more disaggregated manner, limiting the observation of effects in a more specific way. It was also not possible to investigate multiple simultaneous exposures.

It should also be considered as a limitation the inherent memory bias in the study design. In this case, it was further accentuated by the administration of a questionnaire to capture the recall of occupational history, which was necessary for analyzing the association between work and NHL in this study. Each patient was asked to report all occupations held in the last 20 years. Cases may have made more effort to remember possible exposures compared to controls.

It is also worth noting that the study did not include any environmental assessment or clinical evaluation of cases and controls. The only tool used was a data collection instrument, and the information was obtained through interviews. In other words, we only have self-reported exposures mentioned by the interviewees. In an attempt to observe cumulative exposure, only years of exposure to certain carcinogenic agents were considered. There was no way to measure the intensity of each exposure.

At the end of the discussion, the authors should insert a sub-section on the study limitations: selection bias, multiple comparisons, recall bias due to the self-report of the occupational exposure, years of exposure as a poor surrogate of cumulative exposure, lack of exposure assessment by experts to define other exposure metrics, such as intensity and frequency of exposure, the generic definition of the exposure categories, possible confounders unaccounted for (socioeconomic status, agricultural work and exposure to sunlight). The effects of all the above on the risk estimates needs a thorough discussion.

It is important to emphasize that as the study was carried out in a National Institute of the Ministry of Health of Brazil, which is a reference for cancer, and is located in the state of Rio de Janeiro, which is a very populous state in Brazil, it is assumed that the sample included in the study is representative of the general population and its results can be extrapolated to Brazil and other countries with the same socio-demographic and occupational characteristics.

CONCLUSIONS

The data from the present study support the hypothesis that occupational exposure to solvents, hydrocarbons, pesticides, metals, visceral and animal material, and solar radiation is related to an increased chance of developing Non-Hodgkin's Lymphoma in adults. However, occupational exposure to paints, cleaning products, human biological materials, metals and industrial radiation was not statistically associated with this type of neoplasm. The results of this study corroborate other international studies that evaluated and found an association between some occupational exposures and NHL.

However, these results must be viewed with caution, since the results were presented both by the total number of cases and by subtypes of lymphomas, reducing the number of

observations in some cells during the crossing with exposure groups. This form of analysis generated measurements of great magnitude for some subtypes, but with wide confidence intervals. Therefore some without statistical significance, requiring is careful in the evaluations and conclusions. It should also be noted that the study was of an exploratory nature and that it allowed assessing which occupational risks were associated with NHL in Brazil. From this preliminary study we will be able to advance in specific exposures. In several developed countries or in countries with the same socio-demographic and occupational characteristics as Brazil, some agents evaluated here are used intensely, requiring constant monitoring and surveillance of these exposures, exposed workers, and diseases, including cancer, such as NHL. Thus, the results of the present study can contribute to and support strategies in public health and surveillance programs for workers, also will help the various institutions in the world involved in public health actions to design strategies for the prevention and control of work-related cancer.

Acknowledgments

Adriana Scheliga from the Oncology Service of the Brazilian National Cancer Institute (INCA); Employees of Hematology Service and National Tumor Bank (BNT) of the Brazilian National Cancer Institute (INCA).

Authors` contributions

MS and UO designed of the work (project); VIBC, SNF, CAA, PGO, LMV did the acquisition and analysis; VIBC and AOCS performed the statistical analyzes; MS, VIBC, UBO and RH did the interpretation of data; MS, VIBC, SNF, CAA, UBO and CGS have drafted the work or substantively revised it; MS, VIBC, SNF, CAA, PGO, LMV, AOCS, CGS and UBO have approved the submitted version.

Competing interests

The authors declare that they have no competing interests.

REFERENCES

1. Chihara D, Nastoupil LJ, Williams JN, Lee P, Koff JL, Flowers CR. New insights into the epidemiology of non-Hodgkin lymphoma and implications for therapy. Expert Rev Anticancer Ther. 2015;15: 531–544. doi:10.1586/14737140.2015.1023712
2. Vincent T. DeVita, Samuel Hellman SAR. Cancer: Principles and Practice of Oncology. 6th editio. Clinical Trials. 2018.
3. Morton LM, Wang SS, Devesa SS, Hartge P, Weisenburger DD, Linet MS. Lymphoma incidence patterns by WHO subtype in the United States, 1992-2001. Blood. 2006;107: 265–276. doi:10.1182/blood-2005-06-2508
4. Zhang Y, Dai Y, Zheng T, Ma S. Risk factors of non-Hodgkin's lymphoma. Expert Opin Med Diagn. 2011;5: 539–550. doi:10.1517/17530059.2011.618185
5. GLOBOCAN 2018. GLOBOCAN 2018: counting the toll of cancer. The Lancet. Elsevier Ltd; 2018. doi:10.1016/S0140-6736(18)32252-9
6. INCA. Instituto Nacional de Cancer José Alencar Gomes da Silva. Estimativa 2016 - Incidência de Câncer no Brasil. Ministério da Saúde Inst. Nac. Cancer José Alencar Gomes da Silva. 2016. Available: https://www.inca.gov.br/campanhas/dia-nacional-de-combate-ao-cancer/2015/estimativa-2016-incidencia-de-cancer-no-brasil
7. t Mannetje A, De Roos AJ, Boffetta P, Vermeulen R, Benke G, Fritschi L, et al. Occupation and risk of non-hodgkin lymphoma and its subtypes: A pooled analysis from the interlymph consortium. Environ Health Perspect. 2016;124: 396–405. doi:10.1289/ehp.1409294
8. Morton LM, Slager SL, Cerhan JR, Wang SS, Vajdic CM, Skibola CF, et al. Etiologic heterogeneity among non-hodgkin lymphoma subtypes: The interLymph non-hodgkin lymphoma subtypes project. J Natl Cancer Inst Monogr. 2014; 130–144. doi:10.1093/jncimonographs/lgu013
9. Boffetta P. I. Epidemiology of adult non-Hodgkin lymphoma. Annals of Oncology. 2011;22: iv27–iv31. doi:10.1093/annonc/mdr167
10. Eriksson M, Hardell L, Carlberg M, Åkerman M. Pesticide exposure as risk factor for non-Hodgkin lymphoma including histopathological subgroup analysis. Int J Cancer. 2008;123: 1657–1663. doi:10.1002/ijc.23589
11. Karunanayake CP, Dosman JA, Pahwa P. Non-hodgkin's lymphoma and work in agriculture: Results of a two case-control studies in Saskatchewan, Canada. Indian J Occup Environ Med. 2013;17: 114–121. doi:10.4103/0019-5278.130860

12. Lu D, Xu F, Hu K, Yin L, Duan H, Zhang J, et al. Occupational ultraviolet exposure and risk of non-Hodgkin's lymphomas: A meta-analysis. Oncotarget. 2017;8: 62358–62370. doi:10.18632/oncotarget.18140

13. Fritschi L, Benke G, Hughes AM, Kricker A, Vajdic CM, Grulich A, et al. Risk of non-Hodgkin lymphoma associated with occupational exposure to solvents, metals, organic dusts and PCBs (Australia). Cancer Causes Control. 2005;16: 599–607. doi:10.1007/s10552-004-7845-0

14. Cocco P, Satta G, Meloni F, Pilia I, Ahmed F, Becker N, et al. Occupational exposure to organic dust and risk of lymphoma subtypes in the EPILYMPH case–control study. Scand J Work Environ Health. 2021;47: 42–51. doi:10.5271/sjweh.3925

15. Boyle P, Levin B. World Cancer Report 2008. Cancer Control. 2008. doi:10.1016/j.cma.2010.02.010

16. Araújo LH de L, Victorino APO de S, Melo AC de, Assad DX, Lima DS, Alencar DR de, et al. Linfoma Não-Hodgkin de Alto Grau - Revisão da Literatura. Revista Brasileira de Cancerologia. 2008;54: 175–183. doi:10.32635/2176-9745.rbc.2008v54n2.1747

17. Cerhan JR, Kricker A, Paltiel O, Flowers CR, Wang SS, Monnereau A, et al. Medical history, lifestyle, family history, and occupational risk factors for Diffuse Large B-cell Lymphoma: The interLymph non-Hodgkin lymphoma subtypes project. J Natl Cancer Inst Monogr. 2014; 15–25. doi:10.1093/jncimonographs/lgu010

18. Linet MS, Vajdic CM, Morton LM, De Roos AJ, Skibola CF, Boffetta P, et al. Medical history, lifestyle, family history, and occupational risk factors for follicular lymphoma: The interlymph non-hodgkin lymphoma subtypes project. J Natl Cancer Inst Monogr. 2014; 26–40. doi:10.1093/jncimonographs/lgu006

19. Smedby KE, Sampson JN, Turner JJ, Slager SL, Maynadié M, Roman E, et al. Medical history, lifestyle, family history, and occupational risk factors for mantle cell lymphoma: The interlymph non-hodgkin lymphoma subtypes project. J Natl Cancer Inst Monogr. 2014; 76–86. doi:10.1093/jncimonographs/lgu007

20. Wang SS, Flowers CR, Kadin ME, Chang ET, Hughes AM, Ansell SM, et al. Medical history, lifestyle, family history, and occupational risk factors for peripheral T-cell lymphomas: The interlymph non-hodgkin lymphoma subtypes project. J Natl Cancer Inst Monogr. 2014; 66–75. doi:10.1093/jncimonographs/lgu012

21. INCA IN de CJAG da S. Direitos sociais da pessoa com câncer. 2020; 20.

22. Bracci PM, Benavente Y, Turner JJ, Paltiel O, Slager SL, Vajdic CM, et al. Medical history, lifestyle, family history, and occupational risk factors for marginal zone lymphoma: The interLymph non-Hodgkin lymphoma subtypes project. J Natl Cancer Inst Monogr. 2014; 52–65. doi:10.1093/jncimonographs/lgu011

23. Stämpfli MR, Anderson GP. How cigarette smoke skews immune responses to promote infection, lung disease and cancer. Nat Rev Immunol. 2009;9: 377–384. doi:10.1038/nri2530

24. Pearce N, Blair A, Vineis P, Ahrens W, Andersen A, Anto JM, et al. IARC monographs: 40 years of evaluating carcinogenic hazards to humans. Environ Health Perspect. 2015;123: 507–514. doi:10.1289/ehp.1409149

25. Diver WR, Patel A V., Thun MJ, Teras LR, Gapstur SM. The association between cigarette smoking and non-Hodgkin lymphoid neoplasms in a large US cohort study. Cancer Causes and Control. 2012;23: 1231–1240. doi:10.1007/s10552-012-0001-3

26. Luz LL. Non-Hodgkin's Lymphoma: Five-year Survival in a Historical Cohort of Patients who Underwent Stem Cell Transplantation in Brazil. Cancerologia Revista Brasileira de Cancerologia. 2014;60: 35–42.

27. Corrêa MJM, Santana VS. Exposição ocupacional ao benzeno no Brasil: Estimativas baseadas em uma matriz de exposição ocupacional. Cad Saude Publica. 2016;32: 1–11. doi:10.1590/0102-311X00129415

28. Miligi L, Costantini AS, Benvenuti A, Kriebel D, Bolejack V, Tumino R, et al. Occupational exposure to solvents and the risk of lymphomas. Epidemiology. 2006;17: 552–561. doi:10.1097/01.ede.0000231279.30988.4d

29. Steinmaus C, Smith AH, Jones RM, Smith MT. Meta-analysis of benzene exposure and non-Hodgkin lymphoma: Biases could mask an important association. Occup Environ Med. 2008;65: 371–378. doi:10.1136/oem.2007.036913

30. Bassig BA, Friesen MC, Vermeulen R, Shu XO, Purdue MP, Stewart PA, et al. Occupational exposure to benzene and non-hodgkin lymphoma in a population-based cohort: The Shanghai women's health study. Environ Health Perspect. 2015;123: 971–977. doi:10.1289/ehp.1408307

31. Cocco P, T'Mannetje A, Fadda D, Melis M, Becker N, De Sanjosé S, et al. Occupational exposure to solvents and risk of lymphoma subtypes: Results from the Epilymph case-control study. Occup Environ Med. 2010;67: 341–347. doi:10.1136/oem.2009.046839

32. Mehlman MA. Causal relationship between non-Hodgkin's lymphoma and exposure to benzene and benzene-containing solvents. Ann N Y Acad Sci. 2006;1076: 120–128. doi:10.1196/annals.1371.004

33. SEFAZ S da F do E da B. MANUAL DE TRIBUTAÇÃO: PETRÓLEO, GÁS NATURAL, NAFTA PETROQUÍMICA, COMBUSTÍVEIS E LUBRIFICANTES. 2016.

34. Deubler EL, Gapstur SM, Diver WR, Gaudet MM, Hodge JM, Stevens VL, et al. Erythrocyte levels of cadmium and lead and risk of B-cell non-Hodgkin lymphoma and multiple myeloma. Int J Cancer. 2020;147: 3110–3118. doi:10.1002/ijc.33136

35. Fritschi L, Benke G, Hughes AM, Kricker A, Vajdic CM, Grulich A, et al. Risk of non-Hodgkin lymphoma associated with occupational exposure to solvents, metals, organic dusts and PCBs (Australia). Cancer Causes and Control. 2005;16: 599–607. doi:10.1007/s10552-004-7845-0

36. Kelly RS, Lundh T, Porta M, Bergdahl IA, Palli D, Johansson AS, et al. Blood erythrocyte concentrations of cadmium and lead and the risk of B-cell non-Hodgkin's lymphoma and multiple myeloma: A nested case-control study. PLoS One. 2013;8. doi:10.1371/journal.pone.0081892

37. Schinasi L, Leon ME. Non-hodgkin lymphoma and occupational exposure to agricultural pesticide chemical groups and active ingredients: A systematic review and meta-analysis. Int J Environ Res Public Health. 2014;11: 4449–4527. doi:10.3390/ijerph110404449

38. Leon ME, Schinasi LH, Lebailly P, Beane Freeman LE, Nordby KC, Ferro G, et al. Pesticide use and risk of non-Hodgkin lymphoid malignancies in agricultural cohorts from France, Norway and the USA: A pooled analysis from the AGRICOH consortium. Int J Epidemiol. 2019;48: 1519–1535. doi:10.1093/ije/dyz017

39. Fritschi L, Johnson KC, Kliewer E V., Fry R. Animal-related occupations and the risk of leukemia, myeloma, and non-Hodgkin's lymphoma in Canada. Cancer Causes and Control. 2002;13: 563–571. doi:10.1023/A:1016331128897

40. Tranah GJ, Bracci PM, Holly EA. Domestic and farm-animal exposures and risk of non-Hodgkin's lymphoma in a population-based study in the San Francisco Bay Area. Cancer Epidemiology Biomarkers and Prevention. 2008;17: 2382–2387. doi:10.1158/1055-9965.EPI-08-0373

41. Beane Freeman LE, DeRoos AJ, Koutros S, Blair A, Ward MH, Alavanja M, et al. Poultry and livestock exposure and cancer risk among farmers in the agricultural health study. Cancer Causes and Control. 2012;23: 663–670. doi:10.1007/s10552-012-9921-1

42. Karipidis KK, Benke G, Sim MR, Kauppinen T, Kricker A, Hughes AM, et al. Occupational exposure to ionizing and non-ionizing radiation and risk of non-Hodgkin lymphoma. Int Arch Occup Environ Health. 2007;80: 663–670. doi:10.1007/s00420-007-0177-0

43. Tavani A, Bosetti C, Franceschi S, Talamini R, Negri E, La Vecchia C. Occupational exposure to ultraviolet radiation and risk of non-Hodgkin lymphoma. European Journal of Cancer Prevention. 2006;15: 453–457. doi:10.1097/00008469-200610000-00011

44. Boffetta P, van der Hel O, Kricker A, Nieters A, fe Sanjosé S, Maynadié M, et al. Exposure to ultraviolet radiation and risk of malignant lymphoma and multiple myeloma - A multicentre European case - Control study. Int J Epidemiol. 2008;37: 1080–1094. doi:10.1093/ije/dyn092

INVESTIGATION OF OCCUPATIONAL RISK FACTORS FOR THE DEVELOPMENT OF NON-HODGKIN LYMPHOMA IN ADULTS: A HOSPITAL-BASED CASE-CONTROL STUDY

Marcia Sarpa[1,2,*], Vanessa Índio do Brasil da Costa[1], Sâmila Natiane Ferreira[1,2], Carolina Ávila de Almeida[1,2], Paula Gabriela Sousa de Oliveira[1,2], Letícia Vargas de Mesquita[1,2], Arthur OC Schilithz[1], Claudio Gustavo Stefanoff[3], Rocio Hassan[4#], Ubirani Barros Otero[1].

[1]Coordination of Prevention and Surveillance, Brazilian National Cancer Institute. Rua Marquês de Pombal, 125 – Centro, Rio de Janeiro – Brazil. CEP: 20230-240. Phone: +55 (21) 3207-6089.

[2]Environmental Mutagenesis Laboratory, Federal University of Rio de Janeiro State (UNIRIO). Rua Frei Caneca, 94 – Centro, Rio de Janeiro – Brazil. CEP 20211-040. Phone: +55 (21) 2531-7998.

[3]Coordination of Clinical Research and Technological Incorporation, Brazilian National Cancer Institute. Rua André Cavalcanti 37, 2° andar – Bairro de Fátima, Rio de Janeiro, RJ – Brazil. CEP: 20231-050. Phone: +55 (21) 3207-6541.

[4]Oncovirology Laboratory. Bone Marrow Transplantation Center. Brazilian National Cancer Institute (INCA). Praça da Cruz Vermelha, 25 – Centro, Rio de Janeiro – Brazil. CEP: 20230-240. Phone: +55 (21) 3207-1000

[#]in memorium

***Corresponding Author:** Marcia Sarpa.

[1]Institution: Coordination of Prevention and Surveillance. Brazilian National Cancer Institute José Alencar Gomes da Silva. Adress: Rua Marquês de Pombal, 125, 5°andar – Centro, Rio de Janeiro – Brazil. Zipcode: 20230-240. Phone: +55(21)3207-6092. E-mails: mmello@inca.gov.br; marciasarpa@gmail.com

909

ABSTRACT

Non-Hodgkin's Lymphoma (NHL) is a malignancy of the lymphoid lineage of the hematopoietic system and has increased worldwide, especially in developed countries. Better diagnostic and recording techniques, longer life expectancy, and greater exposure to risk factors are hypotheses for this growing incidence curve. Occupational exposures to chemical, biological, and physical agents have also been associated with NHL development, but the results are still controversial. This study described the profile and the past illnesses of the target population, and estimated the association between chemical, biological, and physical risk factors from occupational exposure and the occurrence of NHL in Brazil. This hospital-based observational case-control study was developed at the Cancer Hospital I (HCI), Brazilian National Cancer Institute (INCA), Brazil, between 2013 and 2016. The 214 adult patients were diagnosed as incident cases of NHL through histopathological analysis. The control group included 452 visitors or companions of patients with non-hematologic neoplasia from the HCI/INCA. Socio demographic, clinical, and occupational exposure data were obtained through individual interviews with a standardized questionnaire. Clinical, laboratory, and histopathological data were obtained through medical records. Associations were found between risk of NHL *(any subtype), B-cell lymphoma, and T-cell lymphoma was elevated among the ever exposed to solvents* [give the Odds ratio and 95% confidence interval associated with ever exposure (=all the exposed)], *pesticides* [Odds ratio and 95% CI for the ever exposed], *meat and meat products* [instead of animal and visceral materials] [Odds ratio and 95% CI for the ever exposed], *and sunlight* [instead of solar radiation] [Odds ratio and 95% CI for ever exposed]. *A significant upward trend with years of exposure was detected for solvents and hydrocarbon solvents (NHL (any subtype) p-value for trend), B-cell lymphoma (p for trend), and T-*

Formatted: Strikethrough

Commented [UBO3]: We investigated the association between occupational exposures and risky lifestyle for NHL through a case-control study design with 214 adult patients and 452 population controls.

cell lymphoma (p for trend)), pesticides (NHL (any subtype), p for trend) , B-cell lymphoma (p for trend), DLBCL (p for trend), and T-cell lymphoma (p for trend), raw meat (NHL (any subtype) (p for trend) and DLBCL (p for trend), and sunlight (B-cell lymphoma (p for trend), DLBCL [? Not sure] *(p for trend), and follicular lymphoma* [if still significant after adjustment for agricultural work] *(p for trend).* The project was approved by the Research Ethics Committee of the INCA under registration number 135/11. Associations were found between risk of total NHL any subtypesand, any solvents (OR = 2.38; 95% CI: 1.42–4.00), hydrocarbon solvents (OR = 2.02; 95% CI: 1.17–3.49), pesticides (OR = 3.20; 95% CI: 1.15–8.90), animal and visceral material (OR = 4.67; 95% CI: 1.36–16.05) and solar radiation (OR = 2.22; 95% CI: 1.07–4.62). Furthermore, exposure to solvents and hydrocarbons solvents for more than 10 years increased the chance of developing B-cell and T-cell lymphoma. Exposure to metals, pesticides, animal and visceral material, and solar radiation increased the chance of T-cell lymphoma, B-cell and T-cell lymphoma, B-cell lymphoma and DLBCL, and B-cell lymphoma, respectively. The results of this study are in agreement with other international studies, can be extrapolated to other countries that have the same socio-demographic and occupational characteristics as Brazil and will support strategies for surveillance and control of work-related cancer in the world.

Keywords: Non-Hodgkin Lymphoma; occupational risk factors; lymphoid lineage; pesticides; solvents.

INTRODUCTION

Formatted: Strikethrough

Formatted: Strikethrough

Formatted: Strikethrough

Formatted: Strikethrough

Non-Hodgkin's lymphomas (NHLs) are a group of malignant diseases with more than 40 main subtypes, andwhich have distinct genetic, morphological, and clinical characteristics [1]. They originate from progenitor cells and mature B cells, from progenitor and mature T-cells or from natural killer cells (rarely), which vary in pathophysiology, histological appearance, clinical course, and response to therapy [2]. The most common subtypes are diffuse large B-cell and follicular lymphomas, together accounting for approximately 30 and 20% of all NHL cases, respectively [3,4]. Other less common subtypes are Burkitt lymphoma (1–5% of adult lymphoma cases); lymphoplasmacytic lymphoma (rare and indolent); chronic lymphocytic leukemia/small cell lymphocytic lymphoma; marginal zone lymphoma (5–10% of NHL cases); mantle cell lymphoma (2–4% of NHL cases); peripheral T-cell lymphoma, which the WHO defines as "natural killer" and mature T-cell neoplasms, and Mycoses fungoides/Sezary syndrome [1].

Formatted: Strikethrough

Formatted: Strikethrough

The highest incidence rates of NHL are found in industrialized countries in North America and Europe, in addition to Australia and New Zealand (> 7.0/100,000 thousand, in both men and women) [5]. Each year, an estimated 509,590 new cases of NHL (2.7% of total cancer) and around 250,000 deaths (2.4% of total deaths) occur worldwide. NHL is among the top ten most common types of cancer in Brazil, each year. For the triennium 2023-2025, an estimated 6,420 and 5,620 new cases will occur annually in Brazilian men and women, respectively. This corresponds to an estimated rate of 6.08 new cases per 100,000 men and 5.08 per 100,000 women. The highest incidence rates of NHL are observed in men in the Southeastern region (8.2/100 thousand) and in women in the Southern Region (7.3/100 thousand) [6].

The best-established risk factors for NHL are immunosuppression; infections, including HIV/AIDS virus, hepatitis C, Epstein Bar; and autoimmune diseases [7]. The proportion of NHL cases related to celiac disease, systemic lupus erythematosus (SLE), Sjogren's syndrome, and rheumatoid arthritis (RA), for example, is considered small, due to the low prevalence of these diseases in the general population [4,8,9]. Hereditary factors are also cited, but they are responsible for only a small portion of NHL cases [9]. Other factors for the development of NHL include smoking (follicular lymphoma) and occupation, especially occupational exposure to specific chemical, physical, and biological agents.

The chemical agents most consistently associated with NHL, mentioned in the literature, are solvents and pesticides [7,10,11]. Other occupational factors related to NHL include solar radiation [12]; ionizing radiation [11]; organic dust (grain, leather, fabric, and wood dusts) C(olocar essas referências aqui de forma numerada Cocco et al., 2021); and mineral and metal dust (Fritschi L et al., 2005); however, evidence of the association of these factors with NHL is innot yet consistent [7].

In a recent review, Micallef et al. [13], using the classification criteria of the The International Agency for Research on Cancer (IARC), classifies 87 occupational exposures in groups 1 and 2A [13]".listed 87 agents from groups 1 and 2A as occupational exposure (68 from group 1 and 19 from group 2A). Nine of these exposures were attributed were specifically associated with non-Hodgkin's lymphoma.to non-Hodgkin lymphomas, with only two considered to have sufficient evidence in humans.

Thus, the aim of the present study was to describe the socio-demographic and clinical data of patients treated at the Brazilian National Cancer Institute, in addition to assess whether there is an association between occupational exposures to chemical, physical and

Formatted: Highlight

Formatted: Strikethrough

Formatted: Strikethrough

Formatted: Body Text 2, Indent: First line: 0.5"

Formatted: Font: Not Italic

Formatted: Font: Not Italic

biological agents and the chance of developing NHL in these patients. Also, it is expected that the results of this study support the implementation of actions to prevent and control exposure to carcinogens in the work environment.

MATERIALS AND METHODS

Study design and population

This is a hospital-based case-control study, with patients residingthat reside in the state of Rio de Janeiro (RJ, Brazil) and were seen at the Brazilian National Cancer Institute (INCA).

It is noteworthy that 17.5 million people live in the 92 municipalities in the state of Rio de Janeiro (RJ, Brazil). Therefore, althoughdespite the case-control study was carried out in a single institute in the country, this institute is a reference hospital and is responsible for providing care to around 7.000 new enrollments patients per year. Also, it is important to emphasize that INCA is a national institute, which belongs to the Ministry of Health of the Federal Government of Brazil, is considered a reference hospital for the care of cancer patients in Brazil, being a High Complexity Assistance Centers in Oncology (CACON). INCA is a national institute, part of the Ministry of Health of the Federal Government of Brazil, responsible for providing care to around 7.000 new patients per year and and INCA is also responsible for formulating public policies on cancer in Brazil.

The sample size calculation was performed using the EpiInfo software version 3.3.2, using the calculation basis for case-control studies, for a confidence interval of 95%. Considering human exposure to the main carcinogens described in the literature associated with lymphomas, it was decided to calculate the sample size was determined using with the

Commented [UBO4]: The project was approved by the Research Ethics Committee of the INCA under registration number 135/11.

Formatted: Font: Not Italic

following parameters: α = 0.05; case control 2:1 ratio; prevalence of occupational hazards among controls of 5% (adopting a more conservative measure; OR = 2.5). Thus, 215 cases and 430 controls would be required to achieve a statistical power of 80%".

for a statistical power of 80%, it will be necessary 215 cases and 430 controls.

Inclusion criteria for the cases were: age between 25 and 75 years; residents of the state of Rio de Janeiro; Brazilian nationality; with a primary diagnosis of NHL confirmed by laboratory and histopathological tests. Exclusion criteria for the cases were: patients diagnosed with HIV were not included. Eligible controls were visitors or companions of patients at INCA/HCI, aged between 25 and 75 years and free from any type of cancer at the time of participating in the study".

Inclusion criteria for the control group were: visitors or companions of patients at INCA/HCI, aged between 25 and 75 years, without previous or current diagnosis of any type of cancer and not accompanying or visiting patients diagnosed with hematological cancer.

During the study, 274 cases and 557 controls matched to cases by gender and age-group (± 10 years) were invited to participate in the study. Of these, 10 cases (3.6%) refused to participate in the study and 50 cases (18.2%) did not meet the previously established eligibility criteria. Regarding controls, 105Regarding controls, 105 subjects (18.9%) did not meet the eligibility criteria or refused to participate in the study. As most visitors of patients diagnosed with non-haematological neoplasms at the hospital's outpatient clinics or wards were female, it was necessary to recruit male blood donors (N = 143) at the hospital Haemotherapy Service. A total of 214 NHL cases (112 men and 102 women) and 452 controls (207 men and 245 women) were included in the study. The The complete diagram with the initial and final number of study participants, including refusals, can be seen in Figure 1.

Formatted: Font: Not Italic, Font color: Auto

Formatted: Justified, Line spacing: Double

Formatted: Font: Not Italic

Formatted: Font: Not Italic

Formatted: Font: Not Italic

Formatted: Font: (Default) Times New Roman, 12 pt, Not Italic, English (United States)

Therefore, this study includes 214 incident patients between the ages of 25 and 75 years with NHL (International Classification of Disease – ICD10; C82-85) diagnosed between 2013 and 2016. Hospital-based cancer registries of cases were identified, and histopathological reports were examined to confirm the diagnosis of NHL, based on the WHO classification: Follicular non-Hodgkin's lymphoma (C82) N cases; Diffuse non-Hodgkin lymphoma (C83) N cases; Cutaneous and peripheral T-cell lymphomas (C84) N cases; Non-Hodgkin lymphoma of other and unspecified types (C85) N cases) [14]. Regarding the cases of NHL, the predominant histological type was B-cell lymphoma (185 cases; 86.0%) and T-cell lymphoma were 30 cases (14.0%).

The studied cases were approximately 2:1 matched, paired by sex and age with the control group. 452 controls without cancer history were selected from the companions and visitors of patients undergoing treatment at HCI/INCA for non-hematological neoplasms and among donors of biological materials with the same sociodemographic profile as the NHL cases. In this study, cases and controls were prospectively accumulated, that is, for each new case diagnosed and included in the study, two new controls were chosen and also included in the study (2:1). Thus avoiding the inclusion of prevalent cases, which would be a potential source of bias.

The letters of informed consent, questionnaires, and all other correspondence with study participants were approved by the INCA Research Ethics Committee under registration number 135/11.

Fig 1. Research participants flow diagram

Data collection

The individual interviews of the research participants were carried out by qualified and trained professionals from the Technical Area Environment, Work and Cancer of the

Formatted: Indent: First line: 0", Space After: 8 pt, Pattern: Clear

Formatted: Strikethrough

National Cancer Institute (INCA; Ministry of Health; Brazil). On the day of the medical appointment or some routine procedure at the hospital, the patients were invited to participate in the research by professionals from the INCA research group. Those patients who agreed to participate in the research signed the Free and Informed Consent Form (TCLE). Data collection was carried out through a standardized and pre-tested questionnaire with questions aimed at obtaining sociodemographic, clinical and occupational information.

In the same period, companions and visitors of patients diagnosed with non-hematological neoplasms undergoing medical care at the outpatient clinics or wards of the Cancer Hospital I (HCI) of the National Cancer Institute (INCA) were invited to participate in the research to compose the control group. In addition, there was a need to recruit donors of biological materials in the Hemotherapy Service (HC1/INCA), since most of the companions and visitors of patients diagnosed with non-hematological neoplasms undergoing medical care at the hospital's outpatient clinics or wards INCA HCI were female. Therefore, on recruitment days, donors of biological materials at the Hemotherapy Service (HC1/INCA) were also invited to participate in the research.

The main outcome of the study was total NHL, and secondary outcomes, such as subtypes B-cell lymphoma, DLBCL, T-cell lymphoma were also included in the study. Occupational exposures were considered as explanatory variables. In addition to these exposures, the literature mentions age, gender, race/ethnicity, family history, autoimmune diseases and some infections as risk factors for NHL.

Thus, in orderTo to characterize the study population and control possible confounding factors, the following information was collected on the*abstracted for analysis* subject's: demographic and clinic variables, including "year of interview" (2013, 2014, 2015

or 2016), “sex” (male, female), “age” (20-39; 40-59; >=60), “race” (white, no white), “region within the state of Rio de Janeiro” (metropolitan, others), “*years at the current residence*time of residence” (≤ 20, >20), “education” (no degree of education or primary school, high school, university degree), “employment actual”: yes (private institution, retired, self-employed, public institution, unnot employed, dependent, pensioner, sick leave), “marital status (no companion, with companion, widowed), “family income - minimum salaries” (mean/SD).

Among the lifestyle variables, we considered the following:In relation to habits and past illness were included: “smoking habit” (never smoked, current smoker, past smoker: ≤ 10 years, past smoker: 10+ years), “smoke load: pack years” (never, daily: <10years, 10+years, others), “cellular phone use” (never, daily: ≤ 10 years, daily: 10+years, others),, “past illnesses” (dengue, pneumonia, HHV8 infection: herpesvirus-8, asma, *H. pylori* infection, diabetes, tuberculosis, HCV infection: hepatitis C virus, malaria, HTLV infection, EBV infection: Epstein-Barr virus). For this last variable (“past illnesses”), more than one response was considered.

Formatted: Font: Not Italic

The occupational history was limited to their main occupations in the last 20 working years and all occupations with working time greater than 1 year were considered. It was decided to establish a cut-off point lasting one year or more greater than 1 year for the exposure time. due to the difficulty of making a causal link between the short exposure time and the development of non-Hodgkin's lymphoma. Inform Information on occupational exposure to chemical substances, physical and biological agents possibly associated with the risk of developing NHL such as solvents, paints, metals, pesticides, cleaning products, biological material, animal and viscera material, industrial radiation and solar radiation (never, <= 10 years, 10+ years) were self-reportedcollected. The participant responded about

Formatted: Font: Not Italic

Formatted: Font: Not Italic

which agents he was exposed to and for how long, in the last 20 years, for each reported occupation. Although the questionnaire contains this information, it was decided to present the results keeping the exposures grouped (any solvents, hydrocarbons solvents, pesticides, metals, visceral and animal material, and solar radiation), without specifying which solvents, metals or pesticides were reported. The description of the exposures reported by the study participants, which agents were contained in each group, can be seen at the bottom of Table 3

Statistical analysis

A descriptive analysis was performed to verify the profile of the studied population. The variables were categorized according to the cutoff points pre-established in the literature. To assess differences in the distributions of categorical variables between the comparison groups (cases and controls), Pearson's chi-square tests or Fisher's exact test (for cases where the variable was dichotomous and there were less than five individuals in a cell) were performed. Missing values were excluded from the analysis and the information was reported in tables 1 and 2. For continuous variables, normality was tested (Kolmogorov test). When the variable had a normal distribution, Student's t-test was used to compare the means; otherwise, the Mann-Whitney non-parametric test was used.

By unconditional logistic regression, multivariate models were estimated to analyse exposure to chemical, physical, and biological agents as well as the risk of NHL according to the cases "Total NHL", "B-cell lymphoma", "DLBCL", "Follicular lymphoma", and "T-cell lymphoma". The covariates used in the study were:" with the following: "Risk of NHL (any subtype), B-cell lymphoma, DLBCL, Follicular lymphoma, and T-cell lymphoma associated with exposure to chemical, physical, and biological agents was analysed by unconditional regression. The

Formatted: English (United States)

Formatted: Font: Not Italic

Formatted: Justified, Indent: First line: 0.49", Line spacing: Double

Formatted: Font: Not Italic

Formatted: Font: Not Italic

Formatted: Font: Not Italic

multivariate regression model included the following covariates:By unconditional logistic regression, multivariate models were estimated to analyze exposure to chemical, physical, and biological agents as well as the risk of NHL according to the cases “Total NHL”, “B-cell lymphoma”, “DLBCL”, “Follicular lymphoma”, and “T-cell lymphoma”. The covariates used in the study were: gender, age, skin color, family income, place of residence, level of education, smoking, alcoholism, past illnesses, and also family history of hematological cancer, including the history of NHL (described in the “Data collection” section and in the footer of table 3). To select the covariates to be included in the final regression model, we followed a stepwise forward procedure testing first the covariates with a p<0.25 association in the univariate analyses of the covariates with NHL (any subtype)[15].To select the covariates that composed the adjustment to the multivariate models, bivariate analyzes of the covariates with the study outcome (NHL) were initially performed, considering a p-value < 0.250 [15]. For multivariate analysis, logistic regression models were used using the manual stepwise progressive (forward) methodology, where the occupational exposure of interest was tested and the adjustment of each model was evaluated by inserting each of the previously selected covariates. The Odds Ratio and its 95% confidence interval were calculated as the measure of association; the null hypothesis was rejected if the 95% confidence interval did not include unity.”

At the end, a 5% significance level was considered for the control of possible confounding assessed using the odds ratio (OR) and its respective 95% confidence interval (95% CI). The software used was SPSS 25.0.

The letters of informed consent, questionnaires, and all other correspondence with study participants were approved by the INCA Research Ethics Committee under registration number 135/11.

Formatted: Font: Not Italic

Formatted: Font: Not Italic

Formatted: Normal, Justified, Don't adjust space between Latin and Asian text, Don't adjust space between Asian text and numbers

RESULTS

A total of 214 NHL cases (112 men and 102 women) and 452 controls (207 men and 245 women) were included in the study. Regarding the cases of NHL, the predominant histological type was B-cell lymphoma (185 cases; 86.0%) and T-cell lymphoma were 30 cases (14.0%).

There was no statistical difference in age (p = 0.063) between the two groups. Most individuals were older than 40 years, with mean age of cases (56 years; standard deviation, SD = 12.7 years) and controls (52.0 years; SD = 12.2). As can be seen, the broad age categorization, the sex, race and marital status did not affect the comparability of cases and controls. An overview of the demographic characteristics of the NHL case–control sets is in Table 1.

Formatted: Line spacing: 1.5 lines

Table 1: Sociodemographic characteristics of the participants in a case-control study, RJ, Brazil, 2013–2016.

Characteristic	Controls		Cases		p-value
-	(n = 452)		(n = 214)		-
-	n	%	n	%	-
**Year of the interview**	-	-	-	-	0.058
2013	116	25.7	58	27.1	-
2014	90	19.9	56	26.2	-
2015	163	36.1	76	35.5	-
2016	83	18.34	24	11.2	-
**Sex**	-	-	-	-	0.135
Male	207	45.8	112	52.3	-
Female	245	54.2	102	47.7	-
**Age**	-	-	-	-	0.063
20 – 39	85	18.8	33	15.3	-
40 – 59	235	52.0	99	46.1	-
≥ 60	132	29.2	82	38.6	-
**Region within the state of Rio de Janeiro**	-	-	-	-	**< 0.001**
Metropolitan	426	94.2	171	79.9	-
Others	26	5.8	43	20.1	-
**Time of residence (Rio de Janeiro State)**	-	-	-	-	0.671
≤ 20	267	59.5	103	57.8	-
> 20	182	40.5	90	42.2	-
No information	3		1		
**Education**	-	-	-	-	**< 0.001**
No degree of education or primary school	124	27.5	101	47.1	-
High school	194	43.0	81	37.9	-
University degree	133	29.5	32	15.0	-
No information	1				
**Employment (actual)**	-	-	-	-	-
Private institution (yes)	111	24.5	25	11.7	**< 0.001**
Retired (yes)	97	21.5	61	28.6	**0.042**
Self-employed (yes)	96	21.2	21	9.9	**0.001**
Public Institution (yes)	71	15.7	14	6.6	**0.001**
Not employed (yes)	63	13.9	25	11.6	0,422
Dependent (yes)	16	3.5	11	5.1	0,322
Pensioner (yes)	13	2.8	14	6.5	0.023
Sick leave (yes)	7	1.5	59	27.4	**< 0.001**
**Marital status**	-	-	-	-	0.075
No companion	125	27.7	73	34.2	-
With companion	309	68.5	129	60.2	-
Widowed	17	3.8	12	5.6	-
No information	1		0		
**Family income (Minimum salaries)**					**< 0.001**
Mean (Std. Dev)		4.1 (5.9)		2.7 (3.6)	

Values correspond to the absolute frequencies of the variables.
The p-value is related to the comparison of variable proportions between NHL cases and controls, using the chi-square or Fisher test (for strata with absolute number < 5).
The p-value corresponds to the comparison of variable means between NHL cases and controls using the Mann-Whitney test
In bold, values with a statistically significant result (< 0.05). 1 minimum salarie in Brazil in 2016 was equal to 880 reais.(220 USD)
* Missing values cases = 1

Formatted: Font color: Red

Formatted: Font color: Red

Formatted: Font color: Red

Formatted: Font color: Red

Formatted: Font color: Red

Table 2 elucidates that there was no statistical difference in smoking status (p = 0.074) between the two groups, although the smoking burden was higher in the cases than in the controls (> 20 pack years: 26.3% vs 12.3%, p < 0.001). Current alcohol consumption (1 to 5 drinks per week) was lower in the cases than in the controls (26.8% vs 40.0%, p = 0.003). Use of cell phones and wireless phones was more frequent and more prolonged among the controls than the cases (p < 0.001). Regarding previous diseases, the cases of NHL presented a higher percentage of diabetes (15.1% vs 5.7%, p < 0.001) and diagnosis of Human T-lymphotropic virus infection (2.9% vs 0.2%, p = 0.005) than the controls.

Table 2: Habits and past illnesses of the participants in a case-control study, RJ, Brazil, 2013-2016.

**Characteristic**	**Controls**			**Cases**		-	**p-value**
-	**(n = 452)**			**(n = 214)**		-	-
-	n	%	-	n	%	-	-
**Smoking status**							0.074
Never smoked	283	65.5	-	117	57.1		
Current smoker	46	10.6	-	20	9.8		
Past smoker (≤ 10 years)	39	9.0	-	30	14.6		
Past smoker (10+ years)	64	14.9	-	38	18.5		
No information	20			9			
**Smoke load (Pack years)**							**< 0.001**
Never	283	65.7	-	117	57.1		
≤ 20	93	21.5	-	34	16.6		
> 20	53	12.3	-	54	26.3		
No information	23			9			
**Alcohol consumption (current)**							**0.003**
Non-drinker	204	46.9	-	120	60.6		
1 to 5 drinks per week	174	40.0		53	26.8		
> 5 drinks per week	57	13.1		25	12.6		
No information	17			16			
**Cellular use**	-	-	-	-	-		**< 0.001**
Never	22	4.9	-	27	13.5		
Daily (≤ 10 years)	144	32.1	-	50	24.0		
Daily (10^{+}years)	224	49.9	-	86	41.3		
Others	59	13.3	-	44	21.2		
No information	3			7			
**Wireless Phone use**	-	-	-	-	-		**< 0.001**
Never	152	34.0	-	106	52.2		
Daily (< 10 years)	61	13.6	-	25	12.2		
Daily (10^{+}years)	94	21.1	-	30	14.6		
Others	140	31.3	-	43	21.0		
No information	5			10			
**Past Illnesses**							
Dengue	145	32.2	-	68	33.0		0.841
Pneumonia	57	12.8	-	34	16.7		0.187
HHV8 infection (Herpesvirus-8)	53	11.8	-	34	16.7		0.097
Asma	45	10.1	-	14	6.8		0.183
H. pylori infection	38	8.4	-	15	7.3		0.610
Diabetes	26	5.7	-	31	15.2		**< 0.001**
Tuberculosis	11	2.7	-	2	1.0		0.246
HVC infection (Hepatitis C virus)	9	2.0	-	5	2.5		0.773
Malaria	1	0.2	-	3	1.4		0.094
HTLV infection	1	0.2	-	6	2.9		**0.005**
EBV infection (Epstein-Barr virus)	1	0.2	-	2	1.0		0.233

Values correspond to the absolute frequencies of the variables.
The p-value is related to the comparison of variable proportions between NHL cases and controls, using the chi-square or Fisher test (for strata with absolute number < 5).
The p-value corresponds to the comparison of variable means between NHL cases and controls using the Mann Whitney test
In bold, values with a statistically significant result (< 0.05).
*Missing values: controls = 2; cases = 8

Table 3 provides the results from multivariate unconditional logistic regression. of occupational exposure to chemical, biological, and physical agents as well as NHL development and histological subtypes. The reference group consisted of individuals who were not occupationally exposed to these substances/agents.

Exposure to any solvents and pesticides was significantly associated with an increased risk of B- and T-cell lymphoma. We observed a positive dose-response relationship between years of exposure to these substances and increased risk for total NHL.

For occupational exposure to any solvents, hydrocarbons solvents and pesticides, the chance of developing total NHL was 2.12, 1.91 and 2.70 times greater, respectively, in the cases exposed for more than 10 years, compared to the controls (OR = 2.12 95%CI 1.30-3.48; OR = 1.91 95%CI 1.14-3.21; OR = 2.70 95%CI 1.01-7.21).

B-cell lymphoma was significantly associated with occupational exposure to any of those solvents (> 10 years: OR = 1.92; 95%CI 1.15-3.21) and hydrocarbon solvents (> 10 years: OR = 1.73; 95%CI 1.01-2.96).

Risk estimates were stronger for T-cell lymphomas, but wide confidence intervals suggest caution in interpreting them. The chance of developing T-cell lymphomas was 3.58 times greater in the cases exposed for more than 10 years compared to the controls (any solvents > 10 years: OR = 3.58 95%CI 1.22-10.48). Inserir p de tendência.

Occupational exposure to pesticides was significantly associated with an increased risk of total NHL, follicular lymphoma and T-cell lymphoma. We observed a dose-response association between years of exposure to these substances and increased risk of total NHL (pesticides>10 years: OR = 2.70 95%CI 1.01-7.21). Strong associations were found between the development of T-cell lymphoma and exposure to pesticides in the workplace. The

chance of developing T-cell lymphomas was 10.65 times greater in the cases exposed to pesticides for more than 10 years, compared to the controls (pesticides>10 years: OR = 10.65; 95%CI 2.35 48.24). The strongest association was observed with risk of T-cell lymphoma, although the small number of subjects suggests caution in the interpretation.but with a large confidence interval.

Formatted: Font: Not Italic

Work with animal and viscera materials increased 4-fold the risk of total NHL, B-cell lymphoma, and Diffuse large B-cell lymphoma (DLBCL) subtypes (> 10 years: OR = 4.19 95%CI 1.22 13.85; OR = 3.29 95%CI 1.06 10.21; OR 4.73 95%CI 1.37 16.73, respectively). The small number of subjects also suggests caution in the interpretation.

We observed that the chance of developing B-cell lymphoma was 2.47 times higher in the cases exposed to solar radiation for more than 10 years, compared to the controls (OR = 2.47 95%CI 1.21 5.04). Overall, the chance that those occupationally exposed for at least 10 years to solar radiation had NHL was more than double compared to the controls. Follicular lymphoma was significantly associated with occupational exposure to solar radiation (> 10 years: OR = 4.49 95%CI 1.40 14.36).

No statistically significant associations were found between some exposures at work (dyes, sanitizing products, biological material, and industrial radiation) and NHL.

Table 3: Occupational risk factors and development of Total NHL, B-cell lymphoma, DLBCL, follicular lymphoma and T-cell lymphoma in the participants in a hospital-based case-control study, RJ, Brazil, 2013-2016.

Risk Factors	Total NHL		B-cell lymphoma		DLBCL		Follicular lymphoma		T-cell lymphoma	
	Controls/ Cases	OR adjusted 95% CI	Controls/ Cases	OR adjusted 95% CI	Controls/ Cases	OR adjusted 95% CI	Controls/ Cases	OR adjusted 95% CI	Controls/ Cases	OR adjusted 95% CI
Chemical										
**Any solvents**										
Never	275/93	1.0	275/83	1.0	275/38	1.0	275/15	1.0	275/10	1.0
≤ 10 years	79/51	**1.70 1.04 to 2.79**[a]	79/43	1.51 0.9 to 2.55[e]	79/18	**1.94 1.01 to 3.73**[g]	79/12	**3.43 1.40 to 8.39**[i]	79/8	**3.42 1.17 to 9.99**[j]
10+ years	77/54	**2.12 1.30 to 3.48**[a]	77/47	**1.92 1.15 to 3.21**[e]	77/19	1.64 0.88 to 3.09[g]	77/11	**3.03 1.25 to 7.31**[i]	77/7	**3.58 1.22 to 10.48**[j]
**p trend**		< 0.001		< 0.001		0.031		0.028		0.023
**Dyes**	-	-					-	-		
Never	402/179	1.0	402/157	-	402/69	-	402/34	-	402/22	-
≤ 10 years	14/9	1.26 0.45 to 3.48[a]	14/7	-	14/3	-	14/1	-	14/2	-
10+ years	15/10	1.60 0.61 to 4.21[a]	15/9	-	15/3	-	15/3	-	15/1	-
**p trend**		0.208								
**Hydrocarbon solvents**		-								
Never	297/107	1.0	297/93	1.0	297/43	1.0	297/18	1.0	297/14	1.0
≤ 10 years	66/43	1.65 0.99 to 2.76[a]	66/38	1.57 0.91 to 2.71[e]	66/16	1.87 0.96 to 3.68[g]	66/10	**2.84 1.15 to 7.00**[k]	66/5	1.66 0.54 to 5.08[j]
10+ years	68/49	**1.91 1.14 to 3.21**[a]	68/42	**1.73 1.01 to 2.96**[e]	68/16	1.40 0.73 to 2.70[g]	68/10	**2.70 1.12 to 6.48**[k]	68/6	2.47 0.86 to 7.08[j]
**p trend**		< 0.001		< 0.001		0.056		0.053		0.169
**Metals**	-									
Never	403/179	1.0	403/158	-	403/69	-	403/35	-	403/21	1.0
≤ 10 years	13/10	1.92 0.65 to 5.72[b]	13/7	-	13/2	-	13/2	-	13/3	3.36 0.77 to 14.71[n]
10+ years	15/9	1.22 0.42 to 3.52[b]	15/8	-	15/4	-	15/1	-	15/1	1.47 0.17 to 12.54[n]
**p trend**		0.176								0.082

Formatted: Centered

Formatted: Centered

Formatted: Centered

Formatted: Centered

Formatted: Centered

Formatted: Centered

Risk Factors	Total NHL		B-cell lymphoma		DLBCL		Follicular lymphoma		T-cell lymphoma	
	Controls/ Cases	OR adjusted 95% CI	Controls/ Cases	OR adjusted 95% CI	Controls/ Cases	OR adjusted 95% CI	Controls/ Cases	OR adjusted 95% CI	Controls/ Cases	OR adjusted 95% CI
**Pesticides**										
Never	390/156	1.0	390/137	1.0	390/59	1.0	390/28	1.0	390/19	1.0
≤ 10 years	20/19	**2.41 1.13 to 5.13**[a]	20/16	2.15 1.0.96 to 4.84[f]	20/5	1.76 0.60 to 5.14[g]	20/8	**4.38 1.61 to 11.93**[j]	20/3	**4.04 1.04 to 15.69**[i]
10^{+} years	10/13	**2.70 1.01 to 7.21**[a]	10/10	2.38 0.84 to 6.77[f]	10/5	2.82 0.86 to 9.19[g]	10/2	3.05 0.56 to 16.78[j]	10/3	**10.65 2.35 to 48.24**[i]
**p trend**		**< 0.001**		0.001		0.035		< 0.001		**0.002**
**Sanitizing products**										
Never	242/95	1.0	242/82	1.0	242/37	1.0	242/16	1.0	242/13	-
≤ 10 years	66/40	1.15 0.65 to 2.02[c]	66/34	1.17 0.65 to 2.14[f]	66/15	1.73 0.86 to 3.52[i]	66/9	2.00 0.80 to 5.02[j]	66/6	-
10^{+} years	62/49	1.41 0.80 to 2.49[c]	62/43	1.47 0.81 to 2.66[f]	62/17	1.76 0.88 to 3.53[i]	62/11	2.21 0.90 to 5.41[j]	62/6	-
**p trend**		0.001		0.001		0.053		0.052		
Biological **Biological material**	-						-			
Never	403/187	1.0	403/164	-	403/71	-	403/37	-	403/23	-
≤ 10 years	19/7	0.35 0.10 to 1.25[b]	19/6	-	19/3	-	19/1	-	19/1	-
10^{+} years	9/4	0.78 0.20 to 2.96[b]	9/3	-	9/1	-	9/0	-	9/1	-
**p trend**		0.656								
**Animal and visceral material**										
Never	417/175	1.0	417/152	1.0	417/66	1.0	417/33	**1.0**	417/23	1.0
≤ 10 years	6/11	**4.16 1.33 to 13.06**[b]	6/10	**4.35 1.38 to 13.67**[e]	6/3	3.12 0.69 to 14.19[h]	6/4	**13.07 2.79 to 61.12**[j]	6/1	3.64 0.39 to 33.99[j]
10^{+} years	8/12	**4.19 1.22 to 13.85**[b]	8/10	**3.29 1.06 to 10.21**[e]	8/6	**4.73 1.37 to 16.73**[h]	8/1	4.27 0.47 to 38.48[j]	8/1	2.92 0.33 to 25.77[j]
**p trend**		**< 0.001**		**< 0.001**		**0.001**		0.156		0.212

Formatted Table

1244

Risk Factors	Total NHL		B-cell lymphoma		DLBCL		Follicular lymphoma		T-cell lymphoma	
	Controls/ Cases	OR adjusted 95% CI	Controls/ Cases	OR adjusted 95% CI	Controls/ Cases	OR adjusted 95% CI	Controls/ Cases	OR adjusted 95% CI	Controls/ Cases	OR adjusted 95% CI
Physical										
**Industrial radiation**										
Never	411/191	1.0	411/167	-	411/72	-	411/37	-	411/24	-
≤ 10 years	11/4	1.04 0.29 to 3.70[d]	11/4	-	11/2	-	11/1	-	11/0	-
10⁺years	9/3	0.28 0.05 to 1.52[d]	9/2	-	9/1	-	9/0	-	9/1	-
**p trend**		0.525								
**Solar radiation (occupational)**										
Never	354/136	1.0	353/116	1.0	353/51	1.0	353/24	1.0	353/20	-
≤ 10 years	56/36	1.51 0.86 to 2.63[a]	56/32	**1.80 1.02 to 3.22**[e]	56/15	**2.13 1.02 to 4.47**[h]	56/9	**4.14 1.64 to 10.45**[m]	56/4	-
10⁺years	21/26	1.91 0.93 to 3.90[a]	21/25	**2.47 1.21 to 5.04**[e]	21/9	2.23 0.90 to 5.58[h]	21/5	**4.49 1.40 to 14.36**[m]	21/1	-
**p trend**		< 0.001		**< 0.001**		0.003		**0.085**		

Total NHL: Total Non-Hodgkin's Lymphoma; DLBCL: Diffuse large B-cell lymphoma; OR: odds ratio; CI: confidence interval.
The data shows missing data.
Any solvents: turpentine, tar, asphalt, benzene, benzine, cleaners or degreaser, synthetic dye hardener, formaldehyde, gasoline, used motor oil, diesel oil, lubricating oil, crude oil, tar, kerosene, reducing agent (not specific), remover (not specs), thinner.
Hydrocarbon solvents: tar, benzene, gasoline, kerosene, thinner (not spec), remover (not spec), thinner.
Metals: chrome, iron, and lead
Pesticides: Pesticides, insecticide, rodenticide, ant killer
[a] OR adjusted for sex, age, place of residence, household income, birthplace, retired, diabetes, cell phone use, wireless phone use, alcohol consumption, HHV8 infection.
[b] OR adjusted for sex, age, place of residence, household income, birthplace, retired, diabetes, cell phone use, wireless phone use, alcohol consumption, HHV8 infection, smoking.
[c] OR adjusted for sex, age, place of residence, household income, birthplace, retired, diabetes, cell phone use, wireless phone use, alcohol consumption.
[d] OR adjusted for sex, age, place of residence, household income, birthplace, retired, diabetes, cell phone use, wireless phone use, HHV8 infection.
[e] OR adjusted for sex, age, place of residence, household income, birthplace, retired, diabetes, cell phone use.

[f] OR adjusted for sex, age, place of residence, household income, birthplace, retired, diabetes, cell phone use, wireless phone use.

[g] OR adjusted for sex, age, place of residence, place of birth.

[h] OR adjusted for sex, age, place of residence, household income, place of birth.

[i] OR adjusted for sex, age, place of birth.

[j] OR adjusted for sex, age, place of residence, diabetes, year of the interview, HHV8 infection.

[k] OR adjusted for sex, age, place of residence, diabetes, year of the interview.

[l] OR adjusted for sex, age, place of residence, year of the interview.

[m] OR adjusted for sex, age, place of residence, diabetes.

[n] OR adjusted for sex, age, retired, cell phone use, alcohol consumption, HHV8 infection.

The categories with models not estimated in the table refer to bivariate analyzes with $p>0.25$.

Ocupações que mais representam a exposição à exposição a materiais biológicos (Enfermeiro, técnico de enfermagem).

Ocupações que mais representam a exposição à radiação solar (comerciante, doméstica, do lar, eletricista, motorista, pedreiro, pintor, policial militar, segurança, vendedor).

Formatted: Portuguese (Brazil)

Formatted: Portuguese (Brazil)

DISCUSSION

Nesse estudo, encontramos associação entre LNH e as seguintes exposições ocupacionais: solventes totais, hidrocarbonetos, agrotóxicos, sunlight e sunlight, quando comparados àqueles não ocupacionalmente expostos a esses fatores. Essas associações foram mantidas mesmo após ajuste por variáveis que poderiam confundi-las (descritas no rodapé da Tabela 3).

A significant upward trend with years of exposure was detected for solvents and hydrocarbon solvents (NHL any subtype), B-cell lymphoma, and T-cell lymphoma), pesticides (NHL (any subtype), B-cell lymphoma, DLBCL, and T-cell lymphoma, sunlight (NHL (any subtype) and DLBCL), and sunlight (B-cell lymphoma, DLBCL and follicular lymphoma. Não foram encontradas associações entre LNH em expostos a tintas, a metais, a produtos de limpeza a agentes infecciosos, a radiação industrial (any sub-types).

Most of the studied NHL cases were diagnosed as B-cell lymphoma, with a higher incidence of diffuse large B-cell lymphoma (DLBCL) (82; 38.1%) and follicular lymphoma (40; 18.6%). This corresponds to the incidences found in the literature for the different histological subtypes of NHL, which are the high-grade and aggressive subtypes [1,16].

Men exhibited a higher frequency of NHL cases. This demographic profile agrees with estimates of the Brazilian National Cancer Institute [6] and with the profile of cases described in the literature. The mean age of cases was 56 years old (SD = 12.7 years), with the largest portion of individuals over 45, in accordance with the InterLymph consortium [17–20]. It is noteworthy that the broad age categorization did not affect the comparability of cases and controls by gender and case-control status.

Both cases and controls reside in the metropolitan mesoregion of the Rio de Janeiro state. This data has also a limitation. As INCA is a reference hospital in cancer and one of the only ones to offer comprehensive and free treatment for cancer (public health), patients from all over the state of Rio de Janeiro want to be treated at INCA. Therefore some individuals, from others regions, outside the metropolitan region, came to INCA. In addition, it is important to emphasize that most individuals stop working, retire, or take a leave of absence after discovering the disease, which lowers the family income. This could be one of the explanations for the lower income of the cases compared to the controls [21].

For follicular and marginal zone lymphoma, smoking is a risk factor [20,22]. The immunological effects observed in smokers, such as immunosuppression and increased leukocyte production, may be associated with the development of lymphomas [23]. In addition, cigarette smoke contains substances known to be carcinogens, including benzene and formaldehyde [24]. In a cohort study conducted in the United States with 1,926 cases of NHL, smoking (former smokers) was associated with the occurrence of NHL (histological type of T-cells) [25]. The present study found no statistical differences in smoking between the two groups, although the smoke load (pack-years) was higher among the cases than the controls. It is noteworthy that many patients stop smoking after being diagnosed with cancer. Therefore, the answers given by patients when asked about being current smokers may reflect the condition of no longer smoking after the diagnosis of NHL.

No increase in alcoholic beverages consumption was observed among the cases after analyzing the information on the number of doses of alcoholic beverages consumed per week; therefore, the frequency found in the present study (61%) was lower than in other studies [26].

Diabetes had a statistically significant difference between the comparison groups (cases: 15.2%; controls: 5.7%, p < 0.001). This excess risk is different from that found in observational studies, which investigated the association of diabetes and NHL [27]. In case-control studies, an association of diabetes with NHL was observed, without statistical significance (OR: 1.12; 95%CI 0.95–1.31), in other types of studies a risk was found for women (RR: 1.38, 95%CI 1.06–1.80 [28]. Types 1 and 2 diabetes can cause immunosuppression because of immunological dysfunction due to decreased neutrophil activity and alterations in cellular and humoral immunity. In addition, most patients with type 2 diabetes are obese, and it is well established in the literature that excess body fat increases the risk of various malignancies [27]. A limitation of the study related to this variable is that clinical anamnesis and laboratory tests were not performed on controls to confirm the presence or absence of diabetes. Since the result comes from the individual's self-report when asked whether or not they had diabetes.

Formatted: Strikethrough

Like the acquired human immunodeficiency virus (HIV), the Human T-cell lymphotropic virus (HTLV) is a lymphotropic retrovirus, with a predilection for CD4+ T lymphocytes. Transmission occurs through biological fluids such as blood, vaginal fluid, breast milk, and semen . An estimated 5 to 200 million people have been infected with HTLV worldwide [29], and the Brazilian Ministry of Health estimates that between 700,000 and 2 million are infected there, making Brazil the country with the highest number of cases [30] . In our study, 6 (2.9%) of the cases reported being HTLV virus carriers and only 1 (0.2%) of the controls was infected. Statistical analysis demonstrated a predisposition to develop NHL in individuals infected with HTLV (p-value = 0.005). Most HTLV carriers are asymptomatic and only about 5% of those infected will develop some type of complication.

Several diseases associated with HTLV, including neurological, psychiatric, ophthalmological, dermatological, and lymph proliferative manifestations [30] . HTLV-1 is the first oncogenic retrovirus in humans [31] and is considered the etiologic agent of adult T-cell leukemia/lymphoma (ATLL), which can have a broad clinical presentation, ranging from indolent to aggressive. Although the genetic material of HTLV-2 is 70% identical to HTLV-1, it has not yet been related to lymph proliferative diseases. A limitation of the study related to this variable is that laboratory tests were not performed on controls to confirm the presence or absence of HTLV.

Hepatitis C virus (HCV) is a hepatotropic RNA virus transmitted through blood, either by transfusions, sharing needles or biological accidents. Clinical manifestations include acute hepatitis, chronic hepatitis, liver cirrhosis, and autoimmune diseases. A recent meta-analysis performed in USA has shown that HCV is a significant risk factor for NHL, with an odds ratio of 2.6 compared to the non-HCV population [32]. Despite the results found, our study did not find a positive association between contamination by HCV and the risk of developing NHL, with only 5 (2.5%) of the patients being infected, in contrast to 9 (2%) of the controls.

Formatted: Body Text

Among the categories of solvents present in the occupational exposure questionnaire questions, benzene was one of the chemical agents most reported by study participants. In Brazil, according to Corrêa et al (2016) [33], 7,376,761 (8.5%) workers belong to occupational groups potentially exposed to benzene, while 770,212 workers are considered definitively exposed to benzene. Occupational exposure to benzene and other aromatic hydrocarbons and the possibility of developing NHL have been investigated in other works. An Italian study by Miligi et al. [34], which evaluated 1428 cases of NHL and 1530 controls

in eight areas of Italy, through individual interviews and questionnaires, found a positive association for exposure to benzene (OR: 1.60; 95%CI 1.0–2.4) and aromatic hydrocarbons (OR: 1.20; 95%CI 0.9–1.7) between the cases. A meta-analysis found in 22 studies of benzene exposure a 22% increased risk of NHL (OR: 1.22; 95%CI 1.02–1.47). When the estimate was calculated excluding the based studies only in the self-reported occupational history, with 6 studies remaining, the risk was approximately 2 times higher for NHL (OR: 2.12, 95%CI 1.11–4.02) [35]. Other aromatic hydrocarbon solvents (styrene, BTX, toluene, xylene, and others) have been associated with NHL [36–38]. The main mechanism of carcinogenicity is reported to be the genotoxic effect. It is worth mentioning that workers are rarely exposed to a single agent, since workplaces containing benzene, also expose them to a complex mixture of other aromatic hydrocarbons, such as toluene, xylene, ethylbenzene; as well as other hydrocarbons derived from petroleum during the complex processes arising from petroleum refining, the production of coke, oils and naphtha [39].

The wide presence of heavy metals in occupational, industrial, and agricultural environments leads to increased occupational exposure and concern about their health effects [40]. In the present study, não foi encontrada associação entre exposição a metais e LNH global ou por subtipos, embora tenha-se observado uma magnitude de chance importante para lNH global (OR= 1,92 CI:0.65-5.72 para expostos <= 10 anos) e por linfoma folicular (OR= 1,22 CI:0.42-3.52 para expostos >= 10 anos), porém sem significância estatística. O mesmo foi observado para linfoma folicular (OR= 3.36 CI: 0.77-14.71 para expostos <=10 anos e OR=1.47 CI:0.17-12.54 para expostos >= 10 anos), também sem significância estatística. Este resultado é consistente com um estudo realizado na Australia que igualmente avaliou o risco de desenvolvimento de NHL in individuals with occupational exposure to metal dust

did not demonstrate a positive association [42]. but Kelly et al [41] conducted in Italy and Sweden that observed a positive association in women between heavy metal levels in the blood and the risk of developing NHL. Subsequent studies are needed to clarify this association.a associação encontrada neste estudo.

The wide presence of heavy metals in occupational, industrial, and agricultural environments leads to increased occupational exposure and concern about their health effects [40]. In the present study, exposure to heavy metals increased five-fold the risk of developing T-cell lymphoma. This result is in line with a multicenter study by Kelly et al [41] conducted in Italy and Sweden that observed a positive association in women between heavy metal levels in the blood and the risk of developing NHL. Another study in Australia that assessed the risk of developing NHL in individuals with occupational exposure to metal dust did not demonstrate a positive association [42]. Several metals, such as lead and cadmium, are classified as carcinogens, and the hematopoietic system is one of the targets, as alterations in immune function were found [40]. Metal-induced carcinogenesis depends on the level and duration of exposure, in addition to the individual variation in the response of the organism [41]. Data in the literature are still inconsistent and scarce; nevertheless, the results of this study found a positive association between exposure to metals and the development of certain types of NHL. Subsequent studies are needed to clarify this association.

Case-control, cohort, and meta-analysis studies have explored the association between exposure to active ingredients of specific pesticides and the NHL development [43]. The IARC classified some pesticides as exhibiting carcinogenic potential after observing a positive association between pesticides exposure and NHL development. DDT, diazinon,

glyphosate, and malathion have been classified as probably carcinogenic to humans (group 2A) [24]. Furthermore, 2,4-D, chlordane, heptachlor, hexachlorobenzene, lindane, mirex, and pentachlorophenol were classified as possibly carcinogens to humans (group 2B) [24]. Glyphosate and 2,4-D are among the most used pesticides in Brazilian crops. Leon et al. [44] identified an association between NHL and occupational exposure to pesticides in cohorts from France (Agriculture and Cancer – AGRICAN); Norway (Cancer in the Norwegian Agricultural Population – CNAP); and the USA (Agriculture Health Study – AHS). For most of the chemical groups in the pesticides evaluated, no associations were observed in 33 active ingredients. However, increased total NHL risk was observed in farmers who used terbufos; CCL/SLL among those who used deltamethrin; and DLBCL among those who used glyphosate. It should be clarified that terbufos is an extremely toxic organophosphate insecticide; deltramethrin is an insecticide of the pyrethroid group, and glyphosate is an herbicide. All three are widely used in Brazil, including in unauthorized cultures.

The results of this study also showed the association of exposure to animal materials and viscera with an increased chance of developing NHL. These results corroborate the results found in the case-control study conducted in eight Canadian provinces by Fritschi et al. [45], who reported an increased risk between exposure to beef cattle and NHL; as well as by Tranah et al [46] who observed an increased risk of NHL in individuals who lived or worked with cattle (1.6, CI=1.0–2.5) and pigs (OR = 1.5; CI=1.1–2.1). With exposure to pigs for ≥ 5 years, the increased risk also occurred for NHL (OR = 1.8; CI=1.2–2.6) and for diffuse large-cell and immunoblastic large-cell lymphoma (DLCL) (OR = 2.0, CI=1.2-3.4). According to the authors, contact with infectious agents as a result of work with livestock or contact and handling of pork and beef increases the risk of NHL, as they can carry

~~lymphomagenic infectious agents. Freeman et al. [47] analyzing data from the Agricultural Health Study, observed increased risk of NHL (RR = 1.6, 95%CI1.0–2.4) among those who raised poultry and those who were veterinarians on poultry farms (RR = 12.2; 95%CI:1.6–96.3) [46].~~

~~In New Zealand, Mannetje et al. [7] observed a 2-fold higher risk of NHL in workers who handled meat, especially those who worked at meat processing plants, when compared to controls. Although the studies described found some association between exposure and NHL, this association was not for all investigated animals or for all NHL subtypes. Freeman et al. [47] for example found an association for raising poultry but not for sheep; Fritschi et al. [45] found an association between NHL and DLCL for occupational exposure to beef cattle, but not to any other animal.~~

~~Our study indicated that occupational exposure to solar radiation for at least 10 years doubled the chance of total NHL, B-cell lymphomas and DLBCL, compared to the controls. Lu et al. [12] in a meta-analysis with 10 case-control studies and one cohort, in the period between 1997 and 2012, observed a positive association between occupational sun exposure and NHL (OR = 1.15; 95%CI 1.05–1.23), but not for the common subtypes of non-Hodgkin lymphoma (T-cell and B-cell lymphoma, and chronic lymphocytic leukemia). Similarly, Karipidis et al. [48] observed that the group with cumulative occupational exposure and minimum exposure time of 5 years before diagnosis had a 1.46-fold higher chance of having NHL (95%CI 1.06–2.02) than the unexposed. Although the association between occupational sun exposure and NHL was mentioned in the studies described here, other studies have not found this association [49,50]; therefore, more research is needed.~~

The authors considered it important to evaluate the association between subtypes of non-Hodgkin lymphomas and occupational exposures, since few articles carried out this investigation. However, for some types of occupational exposures, such as biological material, paints, metals and industrial radiation, as the number of observations was very small, it was not possible to perform statistical analysis by subtypes of non-Hodgkin lymphoma. Therefore, only the results of the main outcome, total NHL, were evaluated. Furthermore, no signs of effect modification were observed when measures of association were compared for the strata of each variable for total NHL and for the different subtypes of non-Hodgkin's lymphoma.

Formatted: Font: (Default) Times New Roman, 12 pt

Formatted: Justified, Line spacing: Double

Men exhibited a higher frequency of NHL cases. This demographic profile agrees with estimates of the Brazilian National Cancer Institute [6] and with the profile of cases described in the literature. The mean age of cases was 56 years old (SD = 12.7 years), with the largest portion of individuals over 45, in accordance with the InterLymph consortium [17–20]. It is noteworthy that the broad age categorization did not affect the comparability of cases and controls by gender and case-control status.

Both cases and controls reside in the metropolitan mesoregion of the Rio de Janeiro state. This data has also a limitation. As INCA is a reference hospital in cancer and one of the only ones to offer comprehensive and free treatment for cancer (public health), patients from all over the state of Rio de Janeiro want to be treated at INCA. Therefore some individuals, from others regions, outside the metropolitan region, came to INCA. In addition, it is important to emphasize that most individuals stop working, retire, or take a leave of absence after discovering the disease, which lowers the family income. This could be one of the explanations for the lower income of the cases compared to the controls [21].

For follicular and marginal zone lymphoma, smoking is a risk factor [20,22]. The immunological effects observed in smokers, such as immunosuppression and increased leukocyte production, may be associated with the development of lymphomas [23]. In addition, cigarette smoke contains substances known to be carcinogens, including benzene and formaldehyde [24]. In a cohort study conducted in the United States with 1,926 cases of NHL, smoking (former smokers) was associated with the occurrence of NHL (histological type of T-cells) [25]. The present study found no statistical differences in smoking between the two groups. , although the smoke load (pack years) was higher among the cases than the controls. It is noteworthy that many patients stop smoking after being diagnosed with cancer. Therefore, the answers given by patients when asked about being current smokers may reflect the condition of no longer smoking after the diagnosis of NHL.

No increase in alcoholic beverages consumption was observed among the cases after analyzing the information on the number of doses of alcoholic beverages consumed per week; therefore, the frequency found in the present study (61%) was lower than in other studies [26].

Limitações do estudo

Os autores consideram os resultados apresentados como de extrema relevância considerando a história de uso intenso de agentes cancerígenos classificados no Grupo 1 da IARC no país, alguns destes já banidos em outros países. Deve-se pontuar igualmente, a pouca disponibilização de estudos analíticos do tipo caso-controle no Brasil que incluem os fatores de risco ocupacionais para câncer. Outra questão relevante é a possibilidade desses resultados fornecerem ferramentas para a vigilância em saúde do trabalhador de estados e municípios no que diz respeito a exposição de trabalhadores aos agentes trabalhados aqui. Porém,

Formatted: Portuguese (Brazil)

Formatted: Portuguese (Brazil)

Field Code Changed

Formatted: Font: Portuguese (Brazil)

Formatted: Font: (Default) Times New Roman, 12 pt, Portuguese (Brazil)

Formatted: Font: Portuguese (Brazil)

Formatted: Font: (Default) Times New Roman, 12 pt

Formatted: Justified, Line spacing: Double

reconhecemos que esses resultados devem ser vistos com cautela, considerando alguns problemas inerentes ao próprio desenho do estudo. Os controles foram selecionados a partir dos acompanhantes e visitantes dos pacientes com diagnóstico de cânceres sólidos e também na sala de espera do Banco de sangue do Hospital, onde também recebe em sua maioria visitante e acompanhante dos pacientes. Esse procedimento foi adotado para minimizar o efeito de um possível viés de seleção de controles. Dessa forma, os controles selecionados no Banco de sangue não diferiram tanto dos controles captados nas enfermarias do Hospital. Deve-se ressaltar também que o INCA não é um hemocentro.Os autores tentaram minimizar o efeito de um possível viés de seleção de controles ao fazer a captação dos acompanhantes dos pacientes internados. Como esse grupo foi majoritariamente formado por mulheres, foi utilizada outra estratégia de captação, onde no momento da visita de parentes, amigos, vizinhos de pacientes internados, estes são convidados rotineiramente a serem doadores de sangue. Ao aceitarem, foram convidados a fazerem parte da pesquisa. Não foram captados doadores de sangue que não estivessem ali para a visita a um dos pacientes internados. Dessa forma, os doadores não diferiram tanto dos controles captados nas enfermarias. É óbvio que os que aceitaram doar sangue podem diferir um pouco dos que não doaram, em relação ao estado de saúde, considerando os doadores como indivíduos mais saudáveis, continuaram a ser parentes, vizinhos e/ou amigos. Deve-se ressaltar também que o INCA não é uma Instituição de captação de sangue convencional, como um hemocentro.

Outra questão que deve ser abordada, como possível limitação para os achados são as múltiplas comparações trabalhadas aqui. Este é um estudo exploratório onde buscou-se avaliar várias exposições em um único estudo. Por esse motivo, não se pôde avaliar detalhes importantes como ocupação, atividades econômicas e exposições de forma mais desagregada,

limitando a observância dos efeitos de forma mais específica, também não foi possível investigar múltiplas exposições simultâneas.

Há de se considerar também como limitação o viés de memória inerente ao desenho do estudo. Nesse caso, em específico, mais acentuado pela aplicação de um questionário para captação do recordatório do histórico ocupacional, necessário para análise da associação entre trabalho e LNH, nesse estudo. Solicitou-se a cada paciente que relatasse todas as ocupações exercidas nos últimos 20 anos. Possivelmente os casos se esforçaram para lembrar mais de possíveis exposições do que os controles.

Considera-se ainda que o estudo não contou com nenhuma avaliação ambiental ou de avaliação clínica de casos e controles. A única ferramenta utilizada foi um instrumento de coleta de dados, cujas informações foram obtidas por meio de entrevista. Ou seja, temos apenas autorrelatos de exposições referidos pelos entrevistados. Numa tentativa de observar-se a exposição cumulativa, considerou-se apenas anos de exposição a determinados agentes cancerígenos. Não houve como medir a intensidade de cada exposição.

At the end of the discussion, the authors should insert a sub-section on the study limitations: selection bias, multiple comparisons, recall bias due to the self-report of the occupational exposure, years of exposure as a poor surrogate of cumulative exposure, lack of exposure assessment by experts to define other exposure metrics, such as intensity and frequency of exposure, the generic definition of the exposure categories, possible confounders unaccounted for (socioeconomic status, agricultural work and exposure to sunlight). The effects of all the above on the risk estimates needs a thorough discussion.

Formatted: Font: (Default) Times New Roman, 12 pt, English (United States)

Formatted: English (United States)

Formatted: English (United Kingdom)

It is important to emphasize that as the study was carried out in a National Institute of the Ministry of Health of Brazil, which is a reference for cancer, and is located in the state of Rio de Janeiro, which is a very populous state in Brazil, it is assumed that the sample included in the study is representative of the general population and its results can be extrapolated to Brazil and other countries with the same socio-demographic and occupational characteristics.

CONCLUSIONS

The data from the present study support the hypothesis that occupational exposure to solvents, hydrocarbons, pesticides, metals, visceral and animal material, and solar radiation is related to an increased chance of developing Non-Hodgkin's Lymphoma in adults. However, occupational exposure to paints, cleaning products, human biological materials, metals and industrial radiation was not statistically associated with this type of neoplasm. The results of this study corroborate other international studies that evaluated and found an association between some occupational exposures and NHL.

However, these results must be viewed with caution, since the results were presented both by the total number of cases and by subtypes of lymphomas, reducing the number of observations in some cells during the crossing with exposure groups. This form of analysis generated measurements of great magnitude for some subtypes, but with wide confidence intervals. Therefore some without statistical significance, requiring is careful in the evaluations and conclusions. It should also be noted that the study was of an exploratory nature and that it allowed assessing which occupational risks were associated with NHL in Brazil. From this preliminary study we will be able to advance in specific exposures. In

several developed countries or in countries with the same socio-demographic and occupational characteristics as Brazil, some agents evaluated here are used intensely, requiring constant monitoring and surveillance of these exposures, exposed workers, and diseases, including cancer, such as NHL. Thus, the results of the present study can contribute to and support strategies in public health and surveillance programs for workers, also will help the various institutions in the world involved in public health actions to design strategies for the prevention and control of work-related cancer.

Acknowledgments

Adriana Scheliga from the Oncology Service of the Brazilian National Cancer Institute (INCA); Employees of Hematology Service and National Tumor Bank (BNT) of the Brazilian National Cancer Institute (INCA).

Authors` contributions

MS and UO designed of the work (project); VIBC, SNF, CAA, PGO, LMV did the acquisition and analysis; VIBC and AOCS performed the statistical analyzes; MS, VIBC, UBO and RH did the interpretation of data; MS, VIBC, SNF, CAA, UBO and CGS have drafted the work or substantively revised it; MS, VIBC, SNF, CAA, PGO, LMV, AOCS, CGS and UBO have approved the submitted version.

Competing interests

The authors declare that they have no competing interests.

REFERENCES

1. Chihara D, Nastoupil LJ, Williams JN, Lee P, Koff JL, Flowers CR. New insights into the epidemiology of non-Hodgkin lymphoma and implications for therapy. Expert Rev Anticancer Ther. 2015;15: 531–544. doi:10.1586/14737140.2015.1023712
2. Vincent T. DeVita, Samuel Hellman SAR. Cancer: Principles and Practice of

Oncology. 6th editio. Clinical Trials. 2018.

3. Morton LM, Wang SS, Devesa SS, Hartge P, Weisenburger DD, Linet MS. Lymphoma incidence patterns by WHO subtype in the United States, 1992-2001. Blood. 2006;107: 265–276. doi:10.1182/blood-2005-06-2508

4. Zhang Y, Dai Y, Zheng T, Ma S. Risk factors of non-Hodgkin's lymphoma. Expert Opin Med Diagn. 2011;5: 539–550. doi:10.1517/17530059.2011.618185

5. GLOBOCAN 2018. GLOBOCAN 2018: counting the toll of cancer. Lancet. Elsevier Ltd; 2018. doi:10.1016/S0140-6736(18)32252-9

6. INCA. Instituto Nacional de Cancer. Estimativa 2023 - Incidência de Câncer no Brasil. Ministério da Saúde Instituto Nacional de Câncer. 2023. Available: https://www.inca.gov.br/publicacoes/livros/estimativa-2023-incidencia-de-cancer-no-brasil

7. t Mannetje A, De Roos AJ, Boffetta P, Vermeulen R, Benke G, Fritschi L, et al. Occupation and risk of non-hodgkin lymphoma and its subtypes: A pooled analysis from the interlymph consortium. Environ Health Perspect. 2016;124: 396–405. doi:10.1289/ehp.1409294

8. Morton LM, Slager SL, Cerhan JR, Wang SS, Vajdic CM, Skibola CF, et al. Etiologic heterogeneity among non-hodgkin lymphoma subtypes: The interLymph non-hodgkin lymphoma subtypes project. J Natl Cancer Inst - Monogr. 2014; 130–144. doi:10.1093/jncimonographs/lgu013

9. Boffetta P. I. Epidemiology of adult non-Hodgkin lymphoma. Ann Oncol. 2011;22: iv27–iv31. doi:10.1093/annonc/mdr167

10. Eriksson M, Hardell L, Carlberg M, Åkerman M. Pesticide exposure as risk factor for non-Hodgkin lymphoma including histopathological subgroup analysis. Int J Cancer. 2008;123: 1657–1663. doi:10.1002/ijc.23589

11. Karunanayake CP, Dosman JA, Pahwa P. Non-hodgkin's lymphoma and work in agriculture: Results of a two case-control studies in Saskatchewan, Canada. Indian J Occup Environ Med. 2013;17: 114–121. doi:10.4103/0019-5278.130860

12. Lu D, Xu F, Hu K, Yin L, Duan H, Zhang J, et al. Occupational ultraviolet exposure and risk of non-Hodgkin's lymphomas: A meta-analysis. Oncotarget. 2017;8: 62358–62370. doi:10.18632/oncotarget.18140

Formatted: Font: Portuguese (Brazil)

Formatted: Font: (Default) +Body (Calibri), 11 pt, Portuguese (Brazil)

Formatted: Font: Portuguese (Brazil)

13. Marant Micallef C, Shield KD, Baldi I, Charbotel B, Fervers B, Gilg Soit Ilg A, et al. Occupational exposures and cancer: A review of agents and relative risk estimates. Occup Environ Med. 2018;75: 604–614. doi:10.1136/oemed-2017-104858

14. Boyle P, Levin B. World Cancer Report 2008. Cancer Control. 2008. doi:10.1016/j.cma.2010.02.010

15. Hosmer JR, David W.; Lemeshow, Stanley; Sturdivant RX. Applied logistic regression. 3rd ed. John Wiley & Sons, editor. 2013.

16. Araújo LH de L, Victorino APO de S, Melo AC de, Assad DX, Lima DS, Alencar DR de, et al. Linfoma Não-Hodgkin de Alto Grau - Revisão da Literatura. Rev Bras Cancerol. 2008;54: 175–183. doi:10.32635/2176-9745.rbc.2008v54n2.1747

17. Cerhan JR, Kricker A, Paltiel O, Flowers CR, Wang SS, Monnereau A, et al. Medical history, lifestyle, family history, and occupational risk factors for Diffuse Large B-cell Lymphoma: The interLymph non-Hodgkin lymphoma subtypes project. J Natl Cancer Inst - Monogr. 2014; 15–25. doi:10.1093/jncimonographs/lgu010

18. Linet MS, Vajdic CM, Morton LM, De Roos AJ, Skibola CF, Boffetta P, et al. Medical history, lifestyle, family history, and occupational risk factors for follicular lymphoma: The interlymph non-hodgkin lymphoma subtypes project. J Natl Cancer Inst - Monogr. 2014; 26–40. doi:10.1093/jncimonographs/lgu006

19. Smedby KE, Sampson JN, Turner JJ, Slager SL, Maynadié M, Roman E, et al. Medical history, lifestyle, family history, and occupational risk factors for mantle cell lymphoma: The interlymph non-hodgkin lymphoma subtypes project. J Natl Cancer Inst - Monogr. 2014; 76–86. doi:10.1093/jncimonographs/lgu007

20. Wang SS, Flowers CR, Kadin ME, Chang ET, Hughes AM, Ansell SM, et al. Medical history, lifestyle, family history, and occupational risk factors for peripheral T-cell lymphomas: The interlymph non-hodgkin lymphoma subtypes project. J Natl Cancer Inst - Monogr. 2014; 66–75. doi:10.1093/jncimonographs/lgu012

21. INCA IN de CJAG da S. Direitos sociais da pessoa com câncer. 2020; 20. Available: http://www1.inca.gov.br/inca/Arquivos/Titulos/DireitosDireitosdoPacientecomcancer2.pdf.pdf

22. Bracci PM, Benavente Y, Turner JJ, Paltiel O, Slager SL, Vajdic CM, et al. Medical history, lifestyle, family history, and occupational risk factors for marginal zone

Formatted: Font: Portuguese (Brazil)

Formatted: Font: Portuguese (Brazil)

Formatted: Font: Portuguese (Brazil)

lymphoma: The interLymph non-Hodgkin lymphoma subtypes project. J Natl Cancer Inst - Monogr. 2014; 52–65. doi:10.1093/jncimonographs/lgu011

23. Stämpfli MR, Anderson GP. How cigarette smoke skews immune responses to promote infection, lung disease and cancer. Nat Rev Immunol. 2009;9: 377–384. doi:10.1038/nri2530

24. Pearce N, Blair A, Vineis P, Ahrens W, Andersen A, Anto JM, et al. IARC monographs: 40 years of evaluating carcinogenic hazards to humans. Environ Health Perspect. 2015;123: 507–514. doi:10.1289/ehp.1409149

25. Diver WR, Patel A V., Thun MJ, Teras LR, Gapstur SM. The association between cigarette smoking and non-Hodgkin lymphoid neoplasms in a large US cohort study. Cancer Causes Control. 2012;23: 1231–1240. doi:10.1007/s10552-012-0001-3

26. Luz LL. Non-Hodgkin's Lymphoma: Five-year Survival in a Historical Cohort of Patients who Underwent Stem Cell Transplantation in Brazil. Cancerol Rev Bras Cancerol. 2014;60: 35–42. Available: http://www.inca.gov.br/rbc/n_60/v01/pdf/06-artigo-non-hodgkins-lymphoma-five-year-survival-in-a-historical-cohort-of-patients-who-underwent-stem-cell-transplantation-in-brazil.pdf

27. Mitri J, Castillo J, Pittas AG. Diabetes and risk of non-hodgkin's lymphoma: A meta-analysis of observational studies. Diabetes Care. 2008;31: 2391–2397. doi:10.2337/dc08-1034

28. Castillo JJ, Mull N, Reagan JL, Nemr S, Mitri J. Increased incidence of non-Hodgkin lymphoma, leukemia, and myeloma in patients with diabetes mellitus type 2: A meta-analysis of observational studies. Blood. 2012;119: 4845–4850. doi:10.1182/blood-2011-06-362830

29. Ratner L. Predicting the future: Adult T-cell leukemia. Blood. 2020;135: 2013–2014. doi:10.1182/BLOOD.2020005552

30. Da Silva Garcia IF, Hennington ÉA. HTLV: A stigmatizing infection? Cad Saude Publica. 2019;35: 1–14. doi:10.1590/0102-311X00005419

31. Murakami D, Miyashita K, Koyama T, Omori H, Miyamoto Y, Sawatsubashi M, et al. Clinicopathological analysis of sinonasal malignant lymphoma in an HTLV-1 endemic area in Japan -special focus on primary sinonasal diffuse large B-cell lymphoma-. J Clin Exp Hematop. 2019;59: 101–107. doi:10.3960/jslrt.18008

32. Alkrekshi A, Kassem A, Park C, Tse W. Risk of Non-Hodgkin's Lymphoma in HCV Patients in the United States Between 2013 and 2020: A Population-Based Study. Clin Lymphoma, Myeloma Leuk. 2021;21: e832–e838. doi:10.1016/j.clml.2021.06.014

33. Corrêa MJM, Santana VS. Exposição ocupacional ao benzeno no Brasil: Estimativas baseadas em uma matriz de exposição ocupacional. Cad Saude Publica. 2016;32: 1–11. doi:10.1590/0102-311X00129415

34. Miligi L, Costantini AS, Benvenuti A, Kriebel D, Bolejack V, Tumino R, et al. Occupational exposure to solvents and the risk of lymphomas. Epidemiology. 2006;17: 552–561. doi:10.1097/01.ede.0000231279.30988.4d

35. Steinmaus C, Smith AH, Jones RM, Smith MT. Meta-analysis of benzene exposure and non-Hodgkin lymphoma: Biases could mask an important association. Occup Environ Med. 2008;65: 371–378. doi:10.1136/oem.2007.036913

36. Bassig BA, Friesen MC, Vermeulen R, Shu XO, Purdue MP, Stewart PA, et al. Occupational exposure to benzene and non-hodgkin lymphoma in a population-based cohort: The Shanghai women's health study. Environ Health Perspect. 2015;123: 971–977. doi:10.1289/ehp.1408307

37. Cocco P, T'Mannetje A, Fadda D, Melis M, Becker N, De Sanjosé S, et al. Occupational exposure to solvents and risk of lymphoma subtypes: Results from the Epilymph case-control study. Occup Environ Med. 2010;67: 341–347. doi:10.1136/oem.2009.046839

38. Mehlman MA. Causal relationship between non-Hodgkin's lymphoma and exposure to benzene and benzene-containing solvents. Ann N Y Acad Sci. 2006;1076: 120–128. doi:10.1196/annals.1371.004

39. SEFAZ S da F do E da B. MANUAL DE TRIBUTAÇÃO: PETRÓLEO, GÁS NATURAL, NAFTA PETROQUÍMICA, COMBUSTÍVEIS E LUBRIFICANTES. 2016. Available: http://intranet.sefaz.ba.gov.br/scripts/gestao/comunicacao/combustivel_modulo_viii.pdf

40. Deubler EL, Gapstur SM, Diver WR, Gaudet MM, Hodge JM, Stevens VL, et al. Erythrocyte levels of cadmium and lead and risk of B-cell non-Hodgkin lymphoma and multiple myeloma. Int J Cancer. 2020;147: 3110–3118. doi:10.1002/ijc.33136

Formatted: Font: Portuguese (Brazil)

Formatted: Font: Portuguese (Brazil)

41. Kelly RS, Lundh T, Porta M, Bergdahl IA, Palli D, Johansson AS, et al. Blood erythrocyte concentrations of cadmium and lead and the risk of B-cell non-Hodgkin's lymphoma and multiple myeloma: A nested case-control study. PLoS One. 2013;8. doi:10.1371/journal.pone.0081892
42. Fritschi L, Benke G, Hughes AM, Kricker A, Vajdic CM, Grulich A, et al. Risk of non-Hodgkin lymphoma associated with occupational exposure to solvents, metals, organic dusts and PCBs (Australia). Cancer Causes Control. 2005;16: 599–607. doi:10.1007/s10552-004-7845-0
43. Schinasi L, Leon ME. Non-hodgkin lymphoma and occupational exposure to agricultural pesticide chemical groups and active ingredients: A systematic review and meta-analysis. Int J Environ Res Public Health. 2014;11: 4449–4527. doi:10.3390/ijerph110404449
44. Leon ME, Schinasi LH, Lebailly P, Beane Freeman LE, Nordby KC, Ferro G, et al. Pesticide use and risk of non-Hodgkin lymphoid malignancies in agricultural cohorts from France, Norway and the USA: A pooled analysis from the AGRICOH consortium. Int J Epidemiol. 2019;48: 1519–1535. doi:10.1093/ije/dyz017
45. Fritschi L, Johnson KC, Kliewer E V., Fry R. Animal-related occupations and the risk of leukemia, myeloma, and non-Hodgkin's lymphoma in Canada. Cancer Causes Control. 2002;13: 563–571. doi:10.1023/A:1016331128897
46. Tranah GJ, Bracci PM, Holly EA. Domestic and farm-animal exposures and risk of non-Hodgkin's lymphoma in a population-based study in the San Francisco Bay Area. Cancer Epidemiol Biomarkers Prev. 2008;17: 2382–2387. doi:10.1158/1055-9965.EPI-08-0373
47. Beane Freeman LE, DeRoos AJ, Koutros S, Blair A, Ward MH, Alavanja M, et al. Poultry and livestock exposure and cancer risk among farmers in the agricultural health study. Cancer Causes Control. 2012;23: 663–670. doi:10.1007/s10552-012-9921-1
48. Karipidis KK, Benke G, Sim MR, Kauppinen T, Kricker A, Hughes AM, et al. Occupational exposure to ionizing and non-ionizing radiation and risk of non-Hodgkin lymphoma. Int Arch Occup Environ Health. 2007;80: 663–670. doi:10.1007/s00420-007-0177-0

49. Tavani A, Bosetti C, Franceschi S, Talamini R, Negri E, La Vecchia C. Occupational exposure to ultraviolet radiation and risk of non-Hodgkin lymphoma. Eur J Cancer Prev. 2006;15: 453–457. doi:10.1097/00008469-200610000-00011

50. Boffetta P, van der Hel O, Kricker A, Nieters A, fe Sanjosé S, Maynadié M, et al. Exposure to ultraviolet radiation and risk of malignant lymphoma and multiple myeloma - A multicentre European case - Control study. Int J Epidemiol. 2008;37: 1080–1094. doi:10.1093/ije/dyn092

Reply to reviewer – PLOS ONE – Second round - April 2023 – June 2023

This reviewer appreciates the authors' effort to revise the previous version. However, this new version still requires some additional effort. The first and major point is the inclusion of blood donors among the controls to balance the gender structure with the cases. Blood donors, by definition, are healthier than the general population and, therefore, their inclusion makes any attempt of exploring the association with the health history severely and irreparably biased. Nonetheless, the paper has some good points too, which can be saved provided that the authors keep complying with the reviewers' comments and suggestions.

Answer:

The controls were selected from the companions and visitors of patients diagnosed with solid cancers and also from the waiting room of the Hospital's blood bank, which also receives mostly visitors and patients' companions. This procedure was adopted to minimize the effect of a possible control selection bias. In this way, the controls selected at the Blood Bank did not differ from the controls collected in the Hospital wards, as they are also visitors or companions of the patients. It should also be noted that INCA is not a blood center. In order to not cause problems in the interpretation, the term "biological material donors" was replaced by "blood bank controls".

One suggestion would be to omit the analysis of the past health history and focus on the analysis of occupational risk factors. The adjustments by alcohol and smoking can at least partially fix the selection bias related to the blood donors' healthier status. If the authors wish to maintain the analyses on past illnesses, they should limit them to the 309 controls recruited among the patients' companions and visitors.

In any case, the selection bias introduced by using blood donors as controls needs to be openly acknowledged and discussed among the limitations of this study.

The discussion should start with the main results, which are those related to the occupational risk factors. Lifestyle variables were only evaluated in the univariate analysis and smoking, and alcohol only considered as covariates in the regression models. Therefore, a short comment would be enough.

Ok.

Consistently with the bias generated by using blood donors as controls, all the results and discussion on diabetes, HTLV and HCV should be omitted. Those on diabetes and perhaps HCV might be briefly commented upon in the case the authors repeated the analysis after excluding the 143 blood donors. Also, rerun the logistic regressions that included diabetes and HHV8 infection as covariates.

Parts of the diabetes, HTLV, and HCV discussions were omitted as directed.

The results on metals seem also contradictory; a short comparison with the literature findings would be enough.

Ok

From Table 2, it seems that the cases had a lower socio-economic status, but the list of covariates in the footnotes of table 3 do not include any specific adjustment. This might have contributed to generate some possibly spurious excess, such as that with exposure to sunlight. This reviewer suggests including education in the regression models, as listed on line 193 under the subheading on statistical methods as a surrogate for socio-economic status. Also, include occupation as a farmer as a covariate in the logistic regression predicting risk of NHL & subtypes associated with exposure to sunlight

All potential confounding factors of the theoretical model were tested, as well as the level of education was used in the first part of the analysis with the selection criterion (Wald p-value < 0.025). In the multivariate analysis, other socioeconomic variables quantified the variability of response variables, such as place of residence and family income. Only three cases self-declared one of the occupations as a farmer and two as cattle ranchers, therefore, it is not possible to carry out this specific analysis.

At the end of the discussion, the authors should insert a sub-section on the study limitations: selection bias, multiple comparisons, recall bias due to the self-report of the occupational exposure, years of exposure as a poor surrogate of cumulative exposure, lack of exposure assessment by experts to define other exposure metrics, such as intensity and frequency of exposure, the generic definition of the exposure categories, possible confounders unaccounted for (socioeconomic status, agricultural work and exposure to sunlight). The effects of all the above on the risk estimates needs a thorough discussion.

Done.

The text requires an in-depth revision of the style, grammar, and typos. This reviewer went through this type of editing as well for the abstract, introduction, methods and results, but the list in the attached document, though long, might not be exhaustive.

The final version of the article after all the revisions suggested by the reviewers will be sent to the translator.

PLOS One

pone-d-22-29162r1

Investigation of occupational risk factors for the development of non-Hodgkin lymphoma in adults: a hospital-based case-control study.

Additional list of editings.

Abstract

The abstract is too long, includes unnecessary information, and omits important results. For instance, the long sentence in lines 38-45 can be replaced with "*We explored the association of NHL risk occupational and lifestyle exposures* [and health history, if the authors wish to pursue their analysis using only companion or visitor controls] *with a case-control study design in 214 adult patients and 452 population controls in Brazil*."

Ok.

Remove the sentence about ethical approval in lines 47-49 and paste it at the end of the Methods section.

Ok. Removed

The abstract should report the odds ratio and 95% confidence interval associated with ever exposure to the occupational exposures showing a significant association, and the respective *p*-value of the test for trend. The *p*-value of the test for trend should also be reported in Table 3. The lines 49-56 in the abstract should be replaced with the following: "*risk of NHL (any subtype), B-cell lymphoma, and T-cell lymphoma was elevated among the ever exposed to solvents* [give the Odds ratio and 95% confidence interval associated with ever exposure (=all the exposed)], *pesticides* [Odds ratio and 95% CI for the ever exposed], *meat and meat products* [instead of animal and visceral materials] [Odds ratio and 95% CI for the ever exposed], *and sunlight* [instead of solar radiation] [Odds ratio and 95% CI for ever exposed]. *A significant upward trend with years of exposure was detected for solvents and hydrocarbon solvents (NHL (any subtype) p-value for trend), B-cell lymphoma (p for trend), and T-cell lymphoma (p for trend)), pesticides (NHL (any subtype), p for trend) , B-cell lymphoma (p for trend), DLBCL (p for trend), and T-cell lymphoma (p for trend), raw meat (NHL (any subtype) (p for trend) and DLBCL (p for trend), and sunlight (B-cell lymphoma (p for trend), DLBCL* [? Not sure] *(p for trend), and follicular lymphoma* [if still significant after adjustment for agricultural work] *(p for trend)"*.

Ok. It was done.

The results for metals are not worth reporting in the abstract as they are non-significant and show an inverse trend with years of exposure.

Ok.

Drop the sentence in lines 57-58 from "…*can be extrapolated to other countries*" through "…*Brazil*" and, in the following sentence, on line 58, delete "…*will*".

Ok

Introduction

On line 67, replace "*which*" with "*and*".

Ok

Lines 71-72. In all case series this reviewer is aware of, CLL/SLL is among the most frequent mature B-cell subtypes, sometimes the most frequent. It might be less so in South America,or Asia, but certainly is not definable as a "less common" subtype". A lesser frequency might be due to underdiagnosis because of the silent clinical course of most cases. Just omit mentioning CLL here.

Ok

Lines 96 – 98; include the two citations reported here with the name of the first author and year in the list of references, renumber the references progressively in order of citation, and replace name and year with the corresponding numbers throughout the manuscript.

Ok

Line 98: replace "*not yet consistent*" with "*inconsistent*".

Ok

Line 99-101: Marant Micallef only summarized the IARC Working Groups' conclusions, without making any further elaboration. So, this reviewer would suggest replacing the whole sentence with the following "*The International Agency for Research on Cancer classifies 87 occupational exposures in groups 1 and 2A [13]*".

Ok

Line 102: replace "*were attributed to non-Hodgkin's lymphomas*" with "*were specifically associated with non-Hodgkin's lymphoma*".

Ok

Methods

Line 113: replace "*that reside*" with "*residing*".

Ok

Line 114; delete "…*and were*…".

Ok

Line 117: replace "*despite*" with "*although*".

Ok

Lines 118-121: delete from "…*is a reference hospital*…" through "…*Government of Brazil,*"

Ok

Line 123: delete the sentence "…*and INCA is also responsible*…"; insert the following after "…*(CANCON)*": "*INCA is a national institute, part of the Ministry of Health of the Federal Government of Brazil, responsible for providing care to around 7.000 new patients per year and*…".

Ok

Line 128: delete "…*it was decided to calculate*".

Ok

Line 128: insert "…*was determined using*…" between "…*sample size*" and "…*with the following*".

Ok

Line 129: insert "*case-control*" between "*2:1*" and "*ratio*".

Ok

Line 130: delete the closing bracket.

Ok

Lines 130-131: replace "…*for a statistical power of 80%, it will be necessary 215 cases and 430 controls*" with "…*215 cases and 430 controls would be required to achieve a statistical power of 80%*".

Ok

Lines 134-138: replace the sentence currently in lines 134-138 with the following: "*Eligible controls were visitors or companions of patients at INCA/HCI, aged between 25 and 75 years and free from any type of cancer at the time of participating in the study*".

Ok

Line 139: insert the following after "…*557 controls*": "*matched to cases by gender and age-group (± 10 years)*"

Ok

Lines 141: delete "…*previously established*…". Replace "*Regarding controls, 105 subjects (18.9%)*…" with "…*One -hundred-five candidates as controls (18.9%)*…".

Ok

Line 142: In case the authors wish to pursue in their analysis of health history, they should explain here the reason for selecting blood donors and indicate their number (N = 143). This reviewer would suggest including a sentence such as: "*As most visitors of patients diagnosed with non-haematological neoplasms at the hospital's outpatient clinics or wards were female, it was necessary to recruit male blood donors (N = 143) at the hospital Haemotherapy Service*." If the authors decide of omitting the analysis of health history, just delete the whole sentence.

Ok

Lines 149-151: report here the number of cases for each NHL subgroup.

Ok

Lines 152-159: delete the whole paragraph.

Ok

Lines 160-162: move the Ethics note to the end of the Methods section.

Ok

Line 167: delete "*…individual*".

Ok

Lines 178-183. Delete these sentences [see note about line 142].

Ok

Lines 184-188: Delete the whole sentence as it is repetitive.

Ok

Line 189: delete "*Thus, in order…*"

Ok

Line 190: replace "*…collected on the subject's*" with "*…abstracted for analysis*".

Ok

Line 192: delete "*race (white, non white)*". [following my previous review, race was excluded as a covariate]

Ok

Line 193: replace "*time of residence*" with "*years at the current residence*".

Ok

Line 195: replace "*not employed*" with "*unemployed*".

Ok

Line 196-197: Table 1 does not show what was the unit for average income.

Ok

Line 198 and the below paragraph: See the comments on line 142. Also replace "*In relation to habits and past illness were included.*." with "*Among the lifestyle variables, we considered the following:…*".

Ok

Lines 201-204: delete from "*…past illnesses…*" through the end of the sentence.

Ok

Line 205: replace "*…their…*" with "*…the…*"

Ok

Line 206: replace "*working time greater than 1 year*" with "*lasting one year or more*".

Ok

Line 206-209: delete the unnecessary explanation for cutting short-term employments.

Ok

Line 212: "biological material, animal and viscera material". Please, explain what the biological materials would be or whether the authors meant "infectious agents". Also, the animal and viscera material, as this reviewer understand, would be the meat aerosol in abattoirs and butcheries. If so, the definition should be raw meat droplets and aerosols. Some information on what occupation were related to such exposures should be included in the Table 3 footnote.

The question asked through the questionnaire is very broad. You are asked about exposure to biological materials in the work environment (eg such as blood, urine or other exposures).

Line 213: replace "*...collected...*" with "*…self-reported…*".

Ok

Lines 232-235: replace "*By unconditional logistic regression, multivariate models were estimated to analyse exposure to chemical, physical, and biological agents as well as the risk of NHL according to the cases "Total NHL", "B-cell lymphoma", "DLBCL", "Follicular lymphom, and "T-cell lymphoma*". *The covariates used in the study were*:" with the following: "*Risk of NHL (any subtype), B-cell lymphoma, DLBCL, Follicular lymphoma, and T-cell lymphoma associated with exposure to chemical, physical, and biological agents was analysed by unconditional regression. The multivariate regression model included the following covariates:*"

Ok

Line 236: delete "*past illnesses*" and "*also*".

Ok

Lines 238-245: Replace the whole sentence with the following: "*To select the covariates to be included in the final regression model, we followed a stepwise forward procedure testing first the covariates with a p<0.25 association in the univariate analyses of the covariates with NHL (any subtype)[15]*."

Ok

Drop diabetes and HHV8 infection from the covariates in the logistic regression model.

Parts of the diabetes, HTLV, and HCV discussions were omitted as directed.

Lines 245-247 "replace the whole sentence with the following: "*The Odds Ratio and its 95% confidence interval were calculated as the measure of association; the null hypothesis was rejected if the 95% confidence interval did not include unity*."

Ok

Results

The data reported in the first sentence should be moved to the Methods: the number of participants by gender would fit in line 160, before the flow diagram in figure 1; the number of cases of each NHL subtype would fit in lines 234-235. No supplementary material is needed.

Ok

Line 257-259: delete this sentence.

Ok

Table 1. Replace "*time of residence*" with "*years of residence*". Please show what was the unit for family income.

Ok

Lines 280-282: Delete the sentence on past illnesses and the corresponding results from table 2.

Excluded.

Lines 291-293: delete from "…of occupational…" through "…*histological subtypes.*"

Ok

Line 294: insert "*any of*" between "*exposed to*" and "*those substances*..".

Ok

Line 297: Risk increased also for several other subtypes and other exposures. Please. report here the p-value for the tests for trend.

Trend p-value values are described in table 3.

Lines 298-310. Move these sentences from line 296, before the sentence on tests for trend, and report in the text the risks associated with ever exposure (i.e. independent on years of exposure).

Ok

Lines 317-321: move the result on T-cell lymphoma and pesticides before reporting about the tests for trend (line 315). Also, make it shorter as the small number of cases and controls suggests caution. Something like: "*the strongest association was observed with risk of T-cell lymphoma, although the small number of subjects suggests caution in the interpretation*".

Ok

The same suggestion is valid in reporting the results for raw meat aerosols and exposure to sunlight.

Ok

Table 3: Insert risk for ever exposure (all the exposed one or more years independent on years of exposure) for each occupational risk factor. Calculate and report the *p*-value for each test for trend. It is unclear why some results are missing and others with smaller number and presumably more unstable estimates are shown instead (see for instance B-cell lymphoma and dyes, metals, biological agents, and ionizing [and not industrial] radiation or T-cell lymphoma and sanitizing products vs T-cell lymphoma and metals. In the footnote, insert what occupations were more frequently represented among the exposto biological materials and sunlight.

Occupations that most represent exposure to biological materials: Nurse, nursing technician.

Occupations that represent the most exposure to solar radiation: tradesman, housemaid, housewife, electrician, driver, mason, painter, military police, security, salesman.
