# Supplementary material for: Investigation of occupational risk factors for the development of non-Hodgkin’s lymphoma in adults: A hospital-based case-control study
Source: PLoS One. 2024 Feb 26;19(2):e0297140. doi: 10.1371/journal.pone.0297140 (PMC10896545; doi:10.1371/journal.pone.0297140)
Supplement: Attachment — Submitted filename: Response to Reviewers_Engl_PlosOne_NHL_17_March_2023_VF.docx [file pone.0297140.s001.docx]

PONE-D-22-29162

INVESTIGATION OF OCCUPATIONAL RISK FACTORS FOR THE DEVELOPMENT OF NON-HODGKIN LYMPHOMA IN ADULTS: A HOSPITAL-BASED CASE-CONTROL STUDY

PLOS ONE

**Journal requirements:**

When submitting your revision, we need you to address these additional requirements.

1.  Please ensure that your manuscript meets PLOS ONE's style requirements, including those for file naming. The PLOS ONE style templates can be found at

<https://journals.plos.org/plosone/s/file?id=wjVg/PLOSOne_formatting_sample_main_body.pdf>  and

<https://journals.plos.org/plosone/s/file?id=ba62/PLOSOne_formatting_sample_title_authors_affiliations.pdf>

2. Please provide additional details regarding participant consent. In the ethics statement in the Methods and online submission information, please ensure that you have specified what type you obtained (for instance, written or verbal, and if verbal, how it was documented and witnessed). If your study included minors, state whether you obtained consent from parents or guardians. If the need for consent was waived by the ethics committee, please include this information.

**Answer: All participants/volunteers in the case-control study signed a free and informed consent form, agreeing to participate in the research before answering the questionnaires.**

**The study did not include minors.**

3. We note that you have stated that you will provide repository information for your data at acceptance. Should your manuscript be accepted for publication, we will hold it until you provide the relevant accession numbers or DOIs necessary to access your data. If you wish to make changes to your Data Availability statement, please describe these changes in your cover letter and we will update your Data Availability statement to reflect the information you provide.

**Answer: We can provide all data and study information requested by PlosOne. We have the applied questionnaires, the research ethics committee approval term, the informed consent form and all the analyzes carried out.**

4. We note that you have included the phrase “data not shown” in your manuscript. Unfortunately, this does not meet our data sharing requirements. PLOS does not permit references to inaccessible data. We require that authors provide all relevant data within the paper, Supporting Information files, or in an acceptable, public repository. Please add a citation to support this phrase or upload the data that corresponds with these findings to a stable repository (such as Figshare or Dryad) and provide and URLs, DOIs, or accession numbers that may be used to access these data. Or, if the data are not a core part of the research being presented in your study, we ask that you remove the phrase that refers to these data.

**Answer: We made a mistake when writing “data not shown”, because in reality this data was presented in the manuscript. We can send you an excel spreadsheet with all the information on the histological types of lymphomas included in the study if you wish.**

**RESPONSE TO REVIEWERS**

**5. Review Comments to the Author**

Please use the space provided to explain your answers to the questions above. You may also include additional comments for the author, including concerns about dual publication, research ethics, or publication ethics. (Please upload your review as an attachment if it exceeds 20,000 characters)

**Reviewer #1**: The paper is well written. However, unfortunately, there are major biases in the inclusion of cases and controls. Since controls differ largely from cases, it is very hard to draw conclusions from this study.

More cases reside in other regions than controls (20% vs. 5.7%, p < 0.001). The cases had a low
socioeconomic status with a lower educational level (47.2% vs 27.3%, p < 0.001) and family
income (mean minimum wage: 2,763.5 BRL vs 4,303.6 BRL, p > 0.001) than the controls.
The cases included more retirees (28.4% vs 21.4, p = 0.046), pensioners (6.5% vs 2.9%, p =
0.025), and those who received financial assistance from the government (27.4% vs 1.5%, p
222 < 0.001) than in the controls.

**Answer: We appreciate the review and suggestions given by this reviewer, which were extremely important to improve the article.**

**The study carried out was a hospital-based case-control and the basic principle of selection of controls in this type of study is that they need to represent the base population from which the cases came. (Rothman K.J et al. Modern Epidemiology, page 137, paragraph 4). Although cases and controls in this study are different in some social and economic aspects, visitors and/or companions of patients generally come from the same population of origin of the cases and, therefore, have the same chance of being included among the cases and seeking care at the selected hospital if they were to develop cancer. This is justifiable because INCA is a reference institution for cancer treatment in the state of Rio de Janeiro and in Brazil, since most municipalities in the state of Rio de Janeiro do not have hospitals for cancer treatment. In this way, cancer patients within the public health system of Rio de Janeiro, Brazil, are referred to the National Cancer Institute.**

**Furthermore, the data in Table 1 were reviewed and there are no statistically significant differences for the main variables between the control and case groups. Place of residence and family income were considered for adjustment in the multivariate models with the aim of adjusting the model by controlling the effect of these variables individually.**

**The limitation regarding these two variables in the discussion was also described. In both groups, individuals reside in the metropolitan mesoregion of the state of Rio de Janeiro. This data has a limitation, because many individuals can omit the correct place of residence.**

**Regarding the *employment status variables* (retired, pensioners and those who received financial aid from the government), it is known that many patients diagnosed with cancer and undergoing cancer treatment in Brazil stop working, retire, or become pensioners and receive financial aid from the government.**

**It is noteworthy that the use of multivariate analysis models proposes to control and weigh all these differences, whether they are collected through medical records or through direct questions asked using forms.**

**Reviewer #2**:

1) In the last 45 years a number of case-control studies have investigated in detail the risk factors of non-Hodgkin Lymphoma (NHL). In 2001, the U.S. National Cancer Institute promoted the InterLymph Consortium between the research groups conducting studies worldwide. Great achievements have followed in terms of the identification of environmental, behavioural, clinical, and genetic conditions predisposing to the appearance of NHL. The interdisciplinary collaboration within InterLymph has allowed to define specific risk patterns for individual Lymphoma subtypes, although some confusion still persists because of the difficulty in matching the progress in the classification, diagnosis, and treatment of these diseases with the standardization and statistical power requirements of Epidemiology. Unfortunately, studies from various parts of the world, such as Asia, Africa, Middle East, and South America, are not part of InterLymph, because of not matching the requirements for participation or simply because there are no studies out there. Therefore, this reviewer welcomes this Brazilian case-control study and hopes the authors will continue their efforts and do their best to join the InterLymph Consortium. This is a relatively small size study, which would benefit of a more detailed description of the methods and the classification scheme adopted to define the disease entity. There is discussion of the limitations, and the conclusion overinflates the positive findings.

**Answer: Thanks for the review and suggestions given by this reviewer. We believe that they were fundamental for the clarification of the work and for the improvement of the article.**

**As requested, we inserted a paragraph in the conclusion, with the aim of reducing the weight of the positive findings.**

**It is also important to emphasize that the study was exploratory in nature and that it is one of the first studies in Brazil with this objective. The study made it possible to assess which occupational risks were associated with NHL in Brazil. From this preliminary study we will be able to advance and evaluate specific/detailed expositions. It should also be noted that this article presents the first results of this study, in a more global way, and that we intend to better address occupations, economic activities and the development of non-Hodgkin's lymphoma in another article.**

We put in the conclusions that *“the results were presented by the total number of cases, but also by subtypes of lymphomas, reducing the number of observations in some cells during the crossing with exposure groups, which generated measurements of great magnitude for some subtypes, but with wide confidence intervals, and some without statistical significance, requiring parsimony in evaluations and conclusions”.*

**We also include a more detailed description of the methods and classification scheme adopted.**

2) On page 6, row 137, after the sentence ending with “...the diagnosis of NHL.” Please explain whether the diagnosis was based on the WHO classification, and, if so, which revision, or was it the International Classification of Diseases -10th revision. The C82-85 codes reported on page 6, row 135, should indicate that they refer to the ICD-10.

**Answer: The patients (cases) eligible for the present study were: patients diagnosed with a primary tumor of non-Hodgkin's Lymphoma (NHL), with histological confirmation by immunophenotyping, according to the anatomical classifications of the international code of diseases (ICD-10), as described below: Follicular non-Hodgkin's lymphoma (C82); Diffuse non-Hodgkin's lymphoma (C83); Cutaneous and peripheral T-cell lymphomas (C84); Non-Hodgkin's lymphoma of other and unspecified types (C85) (WHO, 2008). Patients diagnosed with HIV were not eligible for the study, as this syndrome is associated with the development of histological subtypes of NHL (CHIU et al., 2003). The diagnosis was also based on the 3rd edition of the World Health Organization - Classification of Hematolymphoid Tumors.**

**Requests for inclusions in the text of the article were answered.**

3) The matching criteria should also described. On page 6, row 137, the authors wrote “The studied cases were paired by sex and age group with 455 healthy individuals (controls)”. First, the correct term would be “approximately 2:1 matched”. Second, within what age range was matching acceptable? Were they 5-year or 10-year age-groups? From Table 1, seems that it was 20–year, which would be quite large. However, based on the small difference in the mean age between cases and controls reported on page 9, rows 212-214, the matching was effective in making cases and controls comparable by age. So, please, explain in the discussion that the broad age categorization did not affect the comparability of cases and controls by gender and case-control status.

**Answer: Cases and controls were matched for age and sex. In this study, the matching age range acceptable was plus or minus 10 years (25 -34; 35 - 44, 45 - 54; 55 - 64; 65 - 75). Due to the better use of the statistical power applied to multiple comparisons combined with the cutoff points pre-established in the literature, the categories of “20-39”, “40-59” and “> 60 years” were considered in the analyses. To verify that the categorization did not affect the comparability of cases and controls, note that the difference observed between groups was not significant.**

4) Third, on rows # 141-142, the authors wrote that “...for each new case diagnosed and included in the study, a new control was chosen...”. Well, the controls were twice as the cases, so at least two controls has to be matched to each case. Fourth, the 455 controls were presumably “healthy” not “all healthy”, as only those with a history of cancer were excluded.

**Answer: The reviewer's requests were considered in the text.**

5) The source of the data is unclear. The rows 167-181 on page 7 describe a list of data that were “collected. A questionnaire and interviews are mentioned but was it a structured interview with a standardized questionnaire? Was the questionnaire administered in person by trained nurses/interviewers or self-administered? Was it a consent form signed before the interview?

**Answer: The individual interviews of the research participants were carried out by qualified and trained professionals from the Environment, Work and Cancer Technical Area of the National Cancer Institute (INCA). Data collection was performed after signing the Free and Informed Consent Form (TCLE). A standardized and pre-tested questionnaire was used with questions aimed at obtaining sociodemographic, clinical and occupational information.**

**On the day of the medical consultation or some routine procedure at the hospital, the patients were invited to participate in the research by professionals from the INCA research group and those who agreed to participate in the research signed the Free and Informed Consent Form (TCLE).**

**In the same period, depending on who entered the hospital, companions and visitors of patients diagnosed with non-hematological neoplasms undergoing medical care at the outpatient clinics or wards of Cancer Hospital I (HCI) of the National Cancer Institute INCA were invited to participate in the survey to compose the group control".**

**In addition, there was a need to recruit donors of biological materials at the Hemotherapy Service (HC1/INCA), because most of the companions and visitors of patients diagnosed with non-hematological neoplasms undergoing medical care at the outpatient clinics or wards of the HCI hospital in INCA were female. Therefore, on recruitment days, donors of biological materials at the Hemotherapy Service (HC1/INCA) were also invited to participate in the research.**

**The Informed Consent Term was signed before the interview and the questionnaire was applied by a health professional from INCA.**

6) Please note that, on page 6 row 150, the number of participating controls is 577, while it is 557 in the flow diagram in figure 1. In the diagram and in the text the refusals to participation are not indicated. While aware of the higher participation rate of hospital controls vs population controls, this reviewer does not believe that every one of the eligible controls accepted. The 20 missing in the count (3.5%) are proportionally identical to the refusals among the cases (which, by the way, is 3.6 not 3.5%).

**Answer: 557 individuals were invited to participate in the control group of the study. However, 102 (18.9%) did not meet the inclusion criteria or refused to participate in the study, as shown in the diagram (Figure 1). As the individuals in the control group were visitors to the wards of patients with non-hematological cancers, the possibility of selection bias was reduced.**

7) The occupational information was limited to the main occupation in the last 20 years lasting one year or more. As this article mainly focuses on occupational risk factors, this reviewer would have expected a more detailed work history. Those unaware of the Brazilian labour market situation, as this reviewer is, cannot judge whether a substantial part of short-term, low-paying and possibly highly exposed jobs were excluded from study.

**Answer: In the occupational history, all occupations with working time greater than 1 year were considered. The research group chose to establish a cut-off point greater than 1 year for exposure time due to the difficulty in making a causal link between short exposure time and the development of non-Hodgkin's lymphoma. The questionnaire contained closed questions about occupational exposures that were self-reported.**

**The vast majority of our participants were over 40 years of age. In Brazil, job turnover 20-30 years ago was less common than today. People valued staying at work, stability. Thus, it is believed that there would be very few reports of less than 1 year of work in our study.**

**In Brazil, the best-known exposures related to the development of lymphomas are the use of pesticides in agriculture and benzene at fuel retail stations, mechanic workshops or refineries. In Brazil, the predominant agricultural model is still family farming. The rural worker, normally, starts his working life in agriculture and remains in it in his youth and in his adult life. Those exposed to benzene mostly work in large refining or fuel distribution companies, in the formal market, which reduces the common turnover observed in informal jobs.**

8) Also, who did assess the exposures? Where these self-reported by the study participants? Was an expert occupational physician/industrial hygienist involved in the exposure assessment? Were jobs and industry coded in a standard format? Was a job-exposure matrix applied to the occupation and industry codes of study subjects? Apparently not, as the generic definitions of solvents, paints, metals, pesticides, cleaning products, biological material, animal and viscera material, industrial radiation and solar radiation seem to suggest. The current standards of occupational epidemiology research are far ahead in respect to this stage. Please, describe this as a limitation in interpreting the findings from this study.

**Answer: The exposures were evaluated by professionals (epidemiologists and toxicologists) who work in the Technical Area Environment, Work and Cancer of the National Cancer Institute.**

**Data related to occupations, economic activities and exposure of cases and controls were collected through a structured individual questionnaire, which contained a detailed occupational history recall. The participant responded about which agents he was exposed to and for how long, in the last 20 years, for each reported occupation.**

**Reports of exposures were recorded from the questionnaire that retrieved the occupational history of each participant, through closed questions, such as: “During occupation X, were you exposed to benzene? (Yes/No)". And so it went on for all the other exhibitions.**

**The authors chose to present the results by lymphoma subtype and keep the exposures grouped (solvents, hydrocarbons, pesticides, metals, visceral and animal material, and solar radiation), without specifying which solvents were reported, which metals or pesticides, etc.**

**Afterwards, specialist professionals from the Environment, Work and Cancer Technical Area, trained in the construction of occupational exposure matrices, which are part of the Carex-Brazil Project (carcinogen exposure-Brazil), worked on these exposure variables that were disaggregated and grouped into groups alike. The description of the exposures reported by the study participants, which were contained in each group, can be seen at the bottom of Table 3 and in the following text:**

Any solvents: turpentine, tar, asphalt, benzene, benzine, cleaners or degreaser, synthetic dye hardener, formaldehyde, gasoline, used motor oil, diesel oil, lubricating oil, crude oil, tar, kerosene, reducing agent (not specific), remover (not specs), thinner.

Hydrocarbon solvents: tar, benzene, gasoline, kerosene, thinner (not spec), remover (not spec), thinner.

Metals: chrome, iron, and lead

Pesticides: Pesticides, insecticide, rodenticide, ant killer

**This information and explanations were placed in the Methods part of the article.**

9) Besides, a little background of what occupations contributed to the exposure to solvents and metals might help the interpretation. Likewise, the prevalent type of crops and some information on the best selling pesticides (insecticides, herbicides and fungicides mainly) in the study area, and not just overall Brazil, might also be helpful.

**Answer: The database analyzed so far has the results of the history of occupations within economic activities, that is, by large groups of economic activities, such as: (a) for solvents = extractive industries, civil construction, water, sewage , waste management and decontamination activities; (b) metals = water, sewage, waste management and decontamination activities, civil construction, manufacturing industry and extractive industry (CNAE: national classification of economic activity, 2017; Brazilian institute of geography and statistics. available at: http:/ /www.cnae.ibge.gov.br/.). The study participant informed/reported his occupation and the project researchers classified the economic activity based on the National Classification of Economic Activity (CNAE).**

**The most predominant types of crops in the state of Rio de Janeiro are vegetables, legumes, tubers and citrus fruits. The most used and sold pesticides in the study area in the year 2017 were glyphosate, paraquat, deltamethrin, picloram, malathion, imidacloprid, lambda-cyhalothrin etc.**

10) On page 7, rows 198-199, the authors wrote: “By unconditional logistic regression, multivariate models were estimated to analyze exposure...” This is an awkward sentence. The authors refer to the use of unconditional logistic regression to estimate the Odds Ratio for specific disease entities associated with the exposures under scrutiny. However, the miracle of multivariate analysis is to adjust the risk estimate of the covariate of interest by the concurrent effect of other covariates. Usually age, gender, and education are included by default. Other covariates might be relevant, such as income, marital status, and residence whether in metropolitan Rio de Janeiro or elsewhere. So, please, list what covariates were included in the logistic model and whether a forward or backwards stepwise procedure was adopted to select them. It is the personal opinion of this reviewer that, in the omics era, race categories based on the colour of the skin do not make any sense and are not surrogate of anything. Therefore, he would suggest to omit it from Table 1 , from page 9, rows 214-215, and from page 8 rows 304-311, or to justify the use of this otherwise useless category.

**Answer: The covariates used in the study were: gender, age, skin color, family income, place of residence, level of education, smoking, alcoholism, past illnesses, and also family history of hematological cancer, including history of NHL (described in the “Data collection” section and in the footer of table 3). To select the covariates that composed the adjustment to the multivariate models, bivariate analyzes of the covariates with the study outcome (NHL) were initially performed, considering a p-value < 0.250 (Hosmer and Lemeshow, 2000). For multivariate analysis, logistic regression models were used using the manual stepwise forward methodology, where the occupational exposure of interest was tested and the adjustment of each model was evaluated by entry each of the previously selected covariates.**

**The general adjustment of the models was performed by testing the hypothesis that the initial model would present an adjustment to the data with the likelihood function, and that it reflects the probability that the estimated model represents the analyzed data set. For this test, the likelihood (-2LL), a chi-square distribution (n –p) corresponding to the degrees of freedom is assumed, in which 'n' represents the number of respondents and 'q' the number of model parameters.**

**Wald statistics and the change in the OR of the exposure of interest when including the potential confounder in the model were also considered. Afterwards, those variables with the best result in the Wald statistic, which caused the most modification in the OR of interest and which reduced the -2LL of the model, were selected for the final model.**

**The text of the methodology referring to this session was modified.**

**The variable skin color/race was removed as suggested by the reviewer.**

11) Table 2 indicates that three controls and one case had a diagnosis of myelodysplasia. Myelodysplastic syndromes were so defined for the first time by the French-American-British Working Group in 1976, and considered as a precursor stage for acute myeloid leukaemia. This seems odd, as the authors declared to have excluded subjects with any form of cancer or visiting parents affected by haematological diseases from the control group. Please, justify the inclusion of the myelodysplasia cases or rerun the analysis after excluding them.

**Answer: Statistical analyzes were performed again excluding the 3 controls and 1 case of myelodysplasia.**

12) Cases of follicular lymphoma were 40 and those of T-cell lymphoma were 26. Why Table 3 shows results for the second and DLBCL, but not for follicular lymphoma? Because of the recent observation of the elevated risk of follicular associated with use of glyphosate, and the note in the discussion about glyphosate being one of the most used pesticides in Brazil, perhaps and analysis of the 40 cases of follicular lymphoma in relation to pesticide use might be interesting.

**Answer: The requested analyzes were carried out and included in table 3.**

13) When presenting the results, the authors gave too much emphasis to the positive associations with risk of T cell lymphoma, which were generated by small numbers. An introductory sentence such as “Risk estimates were stronger for T-cell lymphomas, but the wide confidence intervals suggest caution in interpreting them.” In particular, the 5-fold excess risk of T-cell lymphomas associated with exposure to metals suffers from three drawbacks: first the generic definition of the risk factors, second the extremely wide confidence interval, and third the inverse dose-response trend with years of exposure. Likewise, the almost 17-fold excess risk of T-cell lymphoma associated with generic exposure to pesticides seems to be mainly due to the wild ups and downs in the risk estimates generated by the small number of the exposed for 10 years or more.

**Answer: The results were reviewed with insertion of the introductory phrase suggested by the reviewer. In the conclusion, a sentence was also inserted that reduces the weight initially given to results with positive associations of broad magnitude, but with wide confidence intervals. With the review of the results, the magnitudes of the measures also changed and some were reduced.**

**Regarding the inverse trend of dose-response with years of exposure, we did not observe this. Only a reduction in the magnitude of exposure was observed over periods greater than 10 years for some subtypes and exposures, but the OR was still >1 and, in several crosses results, were statistically significant.**

14) As it concerns the sanitizing products, this reviewer is puzzled by not finding a T-cell lymphoma risk estimate in Table 3, associated with the and 7 cases exposed for less or more than 10 years.

**Answer: In the first analysis performed, the bivariate analysis, no association was found between exposure to sanitizing agents and T-cell lymphoma (p< 0.250), therefore, multivariate analyzes were not performed. This methodology was applied for all exposures and histological types of NHL. The following information has been included in the footnote to Table 3:**

***“Exposure models were not estimated for bivariate analyzes with p>0.25.”***

15) This reviewer also suspects that the association with exposure to solar radiation was mostly, if not entirely, driven by farmers. Were there any non-farmers exposed to solar radiation (fishermen for instance or any other open-air occupations)?

**Answer: The percentage of controls shows a difference in frequency distribution when compared to the percentage of non-Hodgkin's lymphoma cases in general.**

**It can be observed in the first subcolumn (“Controls/Cases”) of each subtype in Table 3 that as the exposure time increases, the cases of lymphoma for all subtypes increase (example below), except for the T-cell subtype.**

| **Solar radiation** | **Control** | | **Case (Total NHL)** | | **Total** | |
| --- | --- | --- | --- | --- | --- | --- |
|  | **N** | **%** | **N** | **%** | **N** | **%** |
| **No exposure** | **353** | **72,2** | **136** | **27,8** | **489** | **100,0** |
| **1 to 10 years** | **56** | **60,9** | **36** | **39,1** | **92** | **100,0** |
| **11 to 20 years** | **21** | **44,7** | **26** | **55,3** | **47** | **100,0** |
| **Total** | **430** | **68,5** | **198** | **31,5** | **628** | **100,0** |

16) The first part of the discussion illustrates the results of lifestyle (smoking and alcohol), and clinical variables (diabetes, viral infections) that were generated by univariate n-by-two tables and not by the multivariate analysis. There are also several drawbacks in the way these were classified; for instance the cut-off for being classified as an alcohol drinker was ridiculous (one drink per week). Considering the U-shaped curve of its relationship of alcohol-related cancer and other diseases, perhaps a categorization of drinkers as occasional and regular would have been more meaningful. Besides, there is no distinction between type 1 and Type 2 diabetes, which is mostly a feature of the metabolic syndrome.

**Answer: A category present in the questionnaire regarding the number of weekly doses of alcoholic beverage per study participant was included in the table.**

**As for the distinction between type 1 and type 2 diabetes, unfortunately we do not have this data. It is a limitation of the study.**

17) Viral diseases, including HTLV infection, were apparently self-reported (there is no mention of serological testing). However, while there is no discussion about the more numerous self-reported cases of HCV, a disproportionately long paragraph is dedicated to discuss the HTLV finding. Both would be worth a short mention with proper caution because of the small study size, the small number of positive answers, and the crude testing of the difference.

**Answer: Request granted and the text has been rewritten. The patients' HTLV cases were checked from the medical records, but the limitation of the study is that the controls did not undergo serological tests.**

18) A long discussion on benzene follows without explaining whether benzene and the other aromatic hydrocarbons were part of the category of hydrocarbon solvents. In fact, such a definition would include also the widely used aliphatic hydrocarbons (such as n-hexane) and the less refined petroleum-derived solvents, such as mineral spirit, gasoline, and naphta. Not to mention the weak finding on metals and T-cell lymphoma or that on solar radiation.

**Answer: Benzene was one of the most reported hydrocarbons by study participants within the solvent category group. There have also been reports of benzene, formaldehyde, gasoline, motor oil, lubricating oil, kerosene, etc. We agree that the discussion cannot have a broad focus only on occupational exposure to benzene, since most workers who are exposed to benzene are also exposed to other agents such as xylene, toluene, ethylbenzene (present in gasoline), different types of oils, greases and other solvents.**

**It should be noted that in the HPA group there is also benzo(a)pyrene (reported by the participants as pitch, asphalt, etc.), used in the production of coke, in the exhaust of diesel engines, gasoline engines, etc., as well as in untreated oils.**

Any solvents: turpentine, tar, asphalt, benzene, benzine, cleaners or degreaser, synthetic dye hardener, formaldehyde, gasoline, used motor oil, diesel oil, lubricating oil, crude oil, tar, kerosene, reducing agent (not specific), remover (not specs), thinner.

Hydrocarbon solvents: tar, benzene, gasoline, kerosene, thinner (not spec), remover (not spec), thinner.

**The discussion text has been rewritten.**

19) The whole discussion needs to be reshaped and shortened. The limitations (small study size, occupational history limited to the longest most recent job, reliance on self reported exposures, generic definition of the relevant occupational risk factor, multiple comparisons) need to be properly highlighted and discussed.

**Answer: Some specific excerpts from the discussion requested by reviewers have been rewritten and redrafted. If it is still necessary, we can make new changes and accept new suggestions from reviewers.**

20) The conclusion, instead, overemphasize the observed positive associations.

**Answer: The conclusion has been restated in order not to overemphasize the observed positive associations.**

**Minor points**

Introduction, page 3, rows 81-83: “NHL is among the top ten most common types of cancer in Brazil, each year, for the triennium 2020-2022. An estimated 6,580 and 5,450 new cases occur annually in Brazilian men and women”.

The triennium 2020-2022 is not over yet. So, the authors might rephrase this sentence. This reviewer suggests something like “...common types of cancer in Brazil, each year. For the triennium 2020-2022, an estimated 6,580 and 5,450 new cases will occur”.

**Answer: Request answered**

Introduction, page 4, row 84: this is a “rate” not a “risk”. Please, use the correct term.

**Answer: Request answered**

Introduction, page 4, rows 97-100: “Other occupational factors related to NHL include solar radiation (12); ionizing radiation (11); organic dust (grain, fabric, and wood dusts); and mineral and metal dust; however, evidence of the association of these factors with NHL is not yet consistent (7).”

Well, these factors have been investigated, but the evidence for their association with an increase in risk is not simply “not consistent”: it does not exist. For instance, the reference # 12 found an inverse association between exposure to solar radiation and NHL risk. Note that reference # 11 refers to agricultural work not ionizing radiation; a range of suitable citations or a review (for instance Schmitz-Feuerhake I et al. Ann Hematol. 2022;101(2):243-250) might better serve the purpose. Also, a citation might accompany the mention of organic dust (Cocco P et al. Scand J Work Environ Health 2021;47(1):42-51) and mineral and metal dust (Fritschi L et al. Cancer Causes Control. 2005;16(5):599-607).

**Answer: Regarding reference number 12, we understand that the authors found a positive association between NHL and occupational exposure to solar radiation, but not between the subtypes. Here's a summary:**

“Occupational sun exposure was positively associated with the risk of NHL 1.14 (95% confidence intervals: 1.05, 1.23; *I^2^*=25.4% p for heterogeneity =0.202) in Caucasian population. Common subtypes of non-Hodgkin lymphoma and ultraviolet exposure had negative results. The pooled odds ratios was 1.16, (95%confidence intervals: 0.90, 1.50) for T-cell non-Hodgkin lymphoma; 0.79, (95%confidence intervals: 0.61, 1.02) for B-cell non-Hodgkin lymphoma; 1.13, (95%confidence intervals: 0.96, 1.34) for chronic lymphocytic leukemia; 1.25, (95%confidence intervals: 0.95, 1.64) for males; 1.49, (95%confidence intervals: 0.99, 2.25) for females.”

**Regarding ionizing radiation, the Schmitz-Feuerhake results can be seen at: [https://www.ncbi.nlm.nih.gov/pmc/articles/PMC8742808/table/Tab3/?report=objectonly] where it presents a series of studies with RR >1 considering occupational exposure and ionizing radiation in different occupations.**

**With regard to dust, a citation of the work by Cocco and Fritchi was inserted, as suggested.**

Methods, page 5, rows 118-119. The comparison with Portugal is unnecessary. Please, delete this sentence.

**Answer: Sentence deleted**

Methods, page 5, row 129. This reviewer is unaware of the need of establishing the sampling error in calculating the statistical power of a case control study. Please make a reference or omit mentioning the sampling error.

**Answer: Request answered**

Methods, page 5, row 132. “OR = 2,5”. Please, use the period to separate the decimal.

**Answer: Request answered**

Page 6, methods, rows 144-154. Please move these two paragraphs immediately after the statistical power paragraph and before indicating the final number of cases included in this study. Please, replace the terms “That way...” with “Therefore...”.

**Answer: Request answered**

Page 6, row 144: the verbal form “were” should be included after “...for [the] cases...”

**Answer: Request answered**

Page 7, row 174. What is the point of mentioning the minimum salaries? Wouldn’t the family income be enough? How was the amount of 5BRL selected for the cut-off? Does it make any sense showing also the mean income in Table 1?

**Answer: In Brazil, the variable "number of minimum salaries" is used to classify individuals into the categories: poor, middle and upper class. There is no cut-off in the amount of 5 Brazilian NMW (National Minimum Wage). This is the number referring to 5 minimum wage. In order not to cause confusion, this value was replaced by the average family income and we put this information in table 1.**

Page 7, row 176. “cellular use” perhaps refers to cellular phone. Please, rephrase.

**Answer: Request answered**

Page 8, row 195. “Missing values were excluded from the analysis”. Table 1 should indicate how many missing values were there for each variable of interest.

**Answer: The “no information”/missing values information was placed in tables 1 and 2.**

Page 8, Results, row 209. “...and T-cell lymphoma (30; 14.0%)”. As it is written seems the authors are saying that T-cell lymphomas were also prevalent. The sentence should be written as it follows “...and T-cell lymphomas were 30 (14.0%).”

**Answer: Request answered**

Page 11, rows 234-235: “We also found a difference in the use of cell and wireless phones (p > 0.001)” and Table 2. The sentence is formulated ambiguously. In fact, the point is that controls used cell phones and wireless phones more than the cases, which might be a consequence of the lower income and lower education of the cases in respect to the controls. Therefore, the phrasing should be explicit about the fact that “use of cell phones and wireless phones was more frequent and more prolonged among the controls than the cases”.

**Answer: Request answered**

Page 13, row 254, same page, row 272: use the terms “years of exposure” instead of “exposure time”.

**Answer: Request answered**

Page 18, row 303: delete the phrase “by histological subtype, and these data are”

**Answer: Request answered**

Page 18, rows 304-311. Delete the whole sentences.

**Answer: Request answered**

Page 18, rows 314-315: “Among the NHL cases, some patients resided in other regions of Rio de Janeiro.” Unclear statement. Does it mean that a proportion of the cases larger than controls resided in regions other than metropolitan RJ?

**Answer: Around 80% of the cases resided in the metropolitan region of the state of Rio de Janeiro and part the same populational base. It is important to note that the other municipalities in Rio de Janeiro are very close to the metropolitan area, since the state of Rio de Janeiro is a small state in terms of geographic area. This variable, place of residence, was controlled in the multivariate analysis.**

Page 19, row 319. Delete the colloquial term “your” and replace it with the article “the”.

**Answer: Request answered**

Page 19, rows 321-327. Smoking is not a strong risk factor for lymphoma, apart from a weak association with risk of follicular lymphoma. Besides, the case-control difference in the proportion of smokers was not the result of the multivariate analysis but of a simple 2x2 table, and current smokers prevailed among the controls. This reviewer suggest the authors to stick to their findings and avoid discussing meaningless findings.

**Answer: The initial objective of the study was to evaluate occupational variables and lymphomas. We may have overestimated the association between the NHL and smoking, because when we look at the “smoke load”, we see that cases smoke more than controls.**

Please, format the list of references in a consistent way following the journal guidelines.

**Answer: The list of references was formatted following the journal's guidelines.**

**Unfortunately, due to the Christmas break and the end of the year, as well as the summer vacation in Brazil, we had little time to answer reviewers' questions. Therefore, we are available to answer questions that have not been clarified.**

**Thanks again for the opportunity and the reviewers' review!**
