# Supplementary material for: Investigation of occupational risk factors for the development of non-Hodgkin’s lymphoma in adults: A hospital-based case-control study
Source: PLoS One. 2024 Feb 26;19(2):e0297140. doi: 10.1371/journal.pone.0297140 (PMC10896545; doi:10.1371/journal.pone.0297140)
Supplement: Attachment — Submitted filename: Brazil paper review 2.docx [file pone.0297140.s002.docx]

This reviewer appreciates the authors’ effort to revise the previous version. However, this new version still requires some additional effort. The first and major point is the inclusion of blood donors among the controls to balance the gender structure with the cases. Blood donors, by definition, are healthier than the general population and, therefore, their inclusion makes any attempt of exploring the association with the health history severely and irreparably biased. Nonetheless, the paper has some good points too, which can be saved provided that the authors keep complying with the reviewers’ comments and suggestions.

One suggestion would be to omit the analysis of the past health history and focus on the analysis of occupational risk factors. The adjustments by alcohol and smoking can at least partially fix the selection bias related to the blood donors’ healthier status. If the authors wish to maintain the analyses on past illnesses, they should limit them to the 309 controls recruited among the patients’ companions and visitors.

In any case, the selection bias introduced by using blood donors as controls needs to be openly acknowledged and discussed among the limitations of this study.

The discussion should start with the main results, which are those related to the occupational risk factors. Lifestyle variables were only evaluated in the univariate analysis and smoking, and alcohol only considered as covariates in the regression models. Therefore, a short comment would be enough.

Consistently with the bias generated by using blood donors as controls, all the results and discussion on diabetes, HTLV and HCV should be omitted. Those on diabetes and perhaps HCV might be briefly commented upon in the case the authors repeated the analysis after excluding the 143 blood donors. Also, rerun the logistic regressions that included diabetes and HHV8 infection as covariates. The results on metals seem also contradictory; a short comparison with the literature findings would be enough.

From Table 2, it seems that the cases had a lower socio-economic status, but the list of covariates in the footnotes of table 3 do not include any specific adjustment. This might have contributed to generate some possibly spurious excess, such as that with exposure to sunlight. This reviewer suggests including education in the regression models, as listed on line 193 under the subheading on statistical methods as a surrogate for socio-economic status. Also, include occupation as a farmer as a covariate in the logistic regression predicting risk of NHL & subtypes associated with exposure to sunlight.

At the end of the discussion, the authors should insert a sub-section on the study limitations: selection bias, multiple comparisons, recall bias due to the self-report of the occupational exposure, years of exposure as a poor surrogate of cumulative exposure, lack of exposure assessment by experts to define other exposure metrics, such as intensity and frequency of exposure, the generic definition of the exposure categories, possible confounders unaccounted for (socioeconomic status, agricultural work and exposure to sunlight). The effects of all the above on the risk estimates needs a thorough discussion.

The text requires an in-depth revision of the style, grammar, and typos. This reviewer went through this type of editing as well for the abstract, introduction, methods and results, but the list in the attached document, though long, might not be exhaustive.

PLOS One

pone-d-22-29162r1
Investigation of occupational risk factors for the development of non-Hodgkin lymphoma in adults: a hospital-based case-control study.

Additional list of editings.

Abstract

The abstract is too long, includes unnecessary information, and omits important results. For instance, the long sentence in lines 38-45 can be replaced with “*We explored the association of NHL risk occupational and lifestyle exposures* [and health history, if the authors wish to pursue their analysis using only companion or visitor controls] *with a case-control study design in 214 adult patients and 452 population controls in Brazil*.”

Remove the sentence about ethical approval in lines 47-49 and paste it at the end of the Methods section.

The abstract should report the odds ratio and 95% confidence interval associated with ever exposure to the occupational exposures showing a significant association, and the respective *p*-value of the test for trend. The *p*-value of the test for trend should also be reported in Table 3. The lines 49-56 in the abstract should be replaced with the following: “*risk of NHL (any subtype), B-cell lymphoma, and T-cell lymphoma was elevated among the ever exposed to solvents* [give the Odds ratio and 95% confidence interval associated with ever exposure (=all the exposed)]*, pesticides* [Odds ratio and 95% CI for the ever exposed]*, meat and meat products* [instead of animal and visceral materials] [Odds ratio and 95% CI for the ever exposed]*, and sunlight* [instead of solar radiation] [Odds ratio and 95% CI for ever exposed]*. A significant upward trend with years of exposure was detected for solvents and hydrocarbon solvents (NHL (any subtype) p-value for trend), B-cell lymphoma (p for trend), and T-cell lymphoma* *(p for trend)), pesticides (NHL (any subtype), p for trend) , B-cell lymphoma (p for trend), DLBCL (p for trend), and T-cell lymphoma (p for trend), raw meat (NHL (any subtype) (p for trend) and DLBCL (p for trend), and sunlight (B-cell lymphoma (p for trend), DLBCL* [? Not sure] *(p for trend), and follicular lymphoma* [if still significant after adjustment for agricultural work] *(p for trend)”.* The results for metals are not worth reporting in the abstract as they are non-significant and show an inverse trend with years of exposure.

Drop the sentence in lines 57-58 from “…*can be extrapolated to other countries*” through “…*Brazil*” and, in the following sentence, on line 58, delete “…*will*”.

Introduction

On line 67, replace “*which*” with “*and*”.

Lines 71-72. In all case series this reviewer is aware of, CLL/SLL is among the most frequent mature B-cell subtypes, sometimes the most frequent. It might be less so in South America, or Asia, but certainly is not definable as a “less common” subtype”. A lesser frequency might be due to underdiagnosis because of the silent clinical course of most cases. Just omit mentioning CLL here.

Lines 96 – 98; include the two citations reported here with the name of the first author and year in the list of references, renumber the references progressively in order of citation, and replace name and year with the corresponding numbers throughout the manuscript.

Line 98: replace “*not yet consistent*” with “*inconsistent*”.

Line 99-101: Marant Micallef only summarized the IARC Working Groups’ conclusions, without making any further elaboration. So, this reviewer would suggest replacing the whole sentence with the following “*The International Agency for Research on Cancer classifies 87 occupational exposures in groups 1 and 2A [13]*”.

Line 102: replace “*were attributed to non-Hodgkin’s lymphomas*” with “*were specifically associated with non-Hodgkin’s lymphoma*”.

Methods

Line 113: replace “*that reside*” with “*residing*”.

Line 114; delete “…*and were*…”.

Line 117: replace “*despite*” with “*although*”.

Lines 118-121: delete from “…*is a reference hospital*…” through “…*Government of Brazil*,”

Line 123: delete the sentence “…*and INCA is also responsible*…”; insert the following after “…(*CANCON)*”: “*INCA is a national institute, part of the Ministry of Health of the Federal Government of Brazil, responsible for providing care to around 7.000 new patients per year and…*“.

Line 128: delete “…*it was decided to calculate*”.

Line 128: insert “…*was determined using*…” between “…*sample size*” and “…*with the following*”.

Line 129: insert “*case-control*” between “*2:1*” and “*ratio*”.

Line 130: delete the closing bracket.

Lines 130-131: replace “…*for a statistical power of 80%, it will be necessary 215 cases and 430 controls*” with “…*215 cases and 430 controls would be required to achieve a statistical power of 80%*”.

Lines 134-138: replace the sentence currently in lines 134-138 with the following: “*Eligible controls were visitors or companions of patients at INCA/HCI, aged between 25 and 75 years and free from any type of cancer at the time of participating in the study*”.

Line 139: insert the following after “…*557 controls*”: “*matched to cases by gender and age-group (± 10 years)*”

Lines 141: delete “…*previously established*…”. Replace “*Regarding controls, 105 subjects (18.9%)*…” with “…*One -hundred-five candidates as controls (18.9%)*…”.

Line 142: In case the authors wish to pursue in their analysis of health history, they should explain here the reason for selecting blood donors and indicate their number (N = 143). This reviewer would suggest including a sentence such as: “*As most visitors of patients diagnosed with non-haematological neoplasms at the hospital's outpatient clinics or wards were female, it was necessary to recruit male blood donors (N = 143) at the hospital Haemotherapy Service*.” If the authors decide of omitting the analysis of health history, just delete the whole sentence.

Lines 149-151: report here the number of cases for each NHL subgroup.

Lines 152-159: delete the whole paragraph.

Lines 160-162: move the Ethics note to the end of the Methods section.

Line 167: delete “…*individual*”.

Lines 178-183. Delete these sentences [see note about line 142].

Lines 184-188: Delete the whole sentence as it is repetitive.

Line 189: delete “*Thus, in order*…”

Line 190: replace “…*collected on the subject’s*” with “…*abstracted for analysis*”.

Line 192: delete “*race (white, non white)*”. [following my previous review, race was excluded as a covariate]

Line 193: replace “*time of residence*” with “*years at the current residence*”.

Line 195: replace “*not employed*” with “*unemployed*”.

Line 196-197: Table 1 does not show what was the unit for average income.

Line 198 and the below paragraph: See the comments on line 142. Also replace “*In relation to habits and past illness were included*..” with “*Among the lifestyle variables, we considered the following*:…”.

Lines 201-204: delete from “…*past illnesses*…” through the end of the sentence.

Line 205: replace “…*their*…” with “…*the*…”

Line 206: replace “*working time greater than 1 year*” with “*lasting one year or more*”.

Line 206-209: delete the unnecessary explanation for cutting short-term employments.

Line 212: “biological material, animal and viscera material”. Please, explain what the biological materials would be or whether the authors meant “infectious agents”. Also, the animal and viscera material, as this reviewer understand, would be the meat aerosol in abattoirs and butcheries. If so, the definition should be raw meat droplets and aerosols. Some information on what occupation were related to such exposures should be included in the Table 3 footnote.

Line 213: replace “...*collected…*” with “…*self-reported…*”.

Lines 232-235: replace “*By unconditional logistic regression, multivariate models were estimated to analyse* *exposure to chemical, physical, and biological agents as well as the risk of NHL according to the cases “Total NHL”, “B-cell lymphoma”, “DLBCL”, “Follicular lymphoma”, and “T-cell lymphoma*”. *The covariates used in the study were*:” with the following: “*Risk of NHL (any subtype), B-cell lymphoma, DLBCL, Follicular lymphoma, and T-cell lymphoma associated with exposure to chemical, physical, and biological agents was analysed by unconditional regression*. *The multivariate regression model included the following covariates:*”

Line 236: delete “*past illnesses*” and “*also*”.

Lines 238-245: Replace the whole sentence with the following: “*To select the covariates to be included in the final regression model, we followed a stepwise forward procedure testing first the covariates with a p<0.25 association in the univariate analyses of the covariates with NHL (any subtype)[15]*.”

Drop diabetes and HHV8 infection from the covariates in the logistic regression model.

Lines 245-247 “replace the whole sentence with the following: “*The Odds Ratio and its 95% confidence interval were calculated as the measure of association; the null hypothesis was rejected if the 95% confidence interval did not include unity*.”

Results

The data reported in the first sentence should be moved to the Methods: the number of participants by gender would fit in line 160, before the flow diagram in figure 1; the number of cases of each NHL subtype would fit in lines 234-235. No supplementary material is needed.

Line 257-259: delete this sentence.

Table 1. Replace “*time of residence*” with “*years of residence*”. Please show what was the unit for family income.

Lines 280-282: Delete the sentence on past illnesses and the corresponding results from table 2.

Lines 291-293: delete from “…of occupational…” through “…*histological subtypes.*”

Line 294: insert ”*any of*” between “*exposed to*” and “*those substances*..”.

Line 297: Risk increased also for several other subtypes and other exposures. Please. report here the p-value for the tests for trend.

Lines 298-310. Move these sentences from line 296, before the sentence on tests for trend, and report in the text the risks associated with ever exposure (i.e. independent on years of exposure).

Lines 317-321: move the result on T-cell lymphoma and pesticides before reporting about the tests for trend (line 315). Also, make it shorter as the small number of cases and controls suggests caution. Something like: “*the strongest association was observed with risk of T-cell lymphoma, although the small number of subjects suggests caution in the interpretation*”.

The same suggestion is valid in reporting the results for raw meat aerosols and exposure to sunlight.

Table 3: Insert risk for ever exposure (all the exposed one or more years independent on years of exposure) for each occupational risk factor. Calculate and report the *p*-value for each test for trend. It is unclear why some results are missing and others with smaller number and presumably more unstable estimates are shown instead (see for instance B-cell lymphoma and dyes, metals, biological agents, and ionizing [and not industrial] radiation or T-cell lymphoma and sanitizing products vs T-cell lymphoma and metals. In the footnote, insert what occupations were more frequently represented among the exposed to biological materials and sunlight.
